# Comparative genomics of sirenians reveals evolution of filaggrin and caspase-14 upon adaptation of the epidermis to aquatic life

**DOI:** 10.1038/s41598-024-60099-2

**Published:** 2024-04-23

**Authors:** Julia Steinbinder, Attila Placido Sachslehner, Karin Brigit Holthaus, Leopold Eckhart

**Affiliations:** Department of Dermatology, Medical University of Vienna, Vienna, Austria

**Keywords:** Differentiation, Molecular evolution, Evolutionary biology

## Abstract

The mammalian epidermis has evolved to protect the body in a dry environment. Genes of the epidermal differentiation complex (EDC), such as *FLG* (filaggrin), are implicated in the barrier function of the epidermis. Here, we investigated the molecular evolution of the EDC in sirenians (manatees and dugong), which have adapted to fully aquatic life, in comparison to the EDC of terrestrial mammals and aquatic mammals of the clade Cetacea (whales and dolphins). We show that the main subtypes of EDC genes are conserved or even duplicated, like late cornified envelope (LCE) genes of the dugong, whereas specific EDC genes have undergone inactivating mutations in sirenians. *FLG* contains premature stop codons in the dugong, and the ortholog of human CASP14 (caspase-14), which proteolytically processes filaggrin, is pseudogenized in the same species. As *FLG* and *CASP14* have also been lost in whales, these mutations represent convergent evolution of skin barrier genes in different lineages of aquatic mammals. In contrast to the dugong, the manatee has retained functional *FLG* and *CASP14* genes. *FLG2* (filaggrin 2) is truncated in both species of sirenians investigated. We conclude that the land-to-water transition of sirenians was associated with modifications of the epidermal barrier at the molecular level.

## Introduction

**Figure 1:**
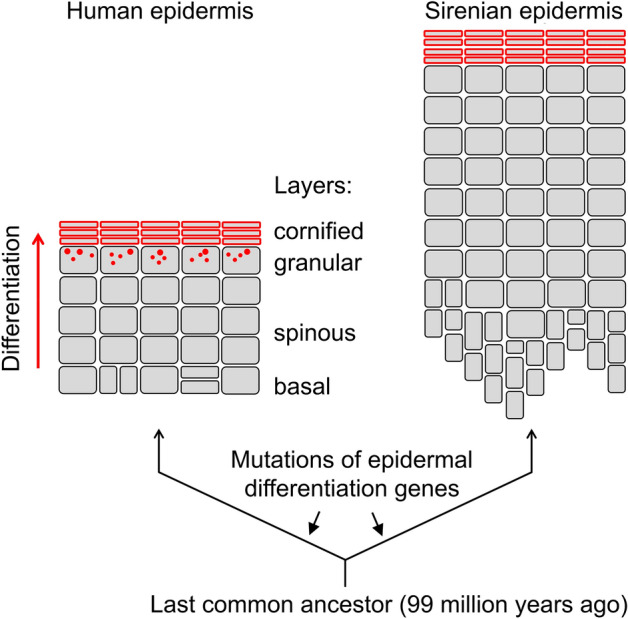
Keratinocyte differentiation and epidermal structure in humans and sirenians. The structure of the epidermis is schematically depicted. Cells are shown as squares with rounded corners. Red borders indicate the cornified envelope, consisting of cross-linked proteins. Red dots indicate keratohyalin granules in the granular layer of human epidermis. Differentiation of keratinocytes leads to the passive movement of cells from the inner to the outer layers and involves cornification, leading to flattening and death of keratinocytes at the surface of the skin.

Life on land depends on the protection against excessive loss of water from the body in a dry environment^1,2^. The control of water loss is mediated in part by the outermost, epithelial compartment of the skin, the epidermis. Within this stratified epithelium, keratinocytes proliferate in the basal layer and differentiate during their movement towards the skin surface while passing through the suprabasal layers (Fig. 1). Keratinocyte differentiation involves the accumulation of cytoskeletal proteins and enzymes that are required for establishing the barrier against the environment which mainly resides in the granular layer and the cornified layer of the epidermis. The transition of keratinocytes from the granular to the cornified layer is associated with programmed cell death and cross-linking of proteins to form a mechanically and chemically resilient protein envelope to which lipids are attached^3–6^. The integrity of cornified cell envelopes is essential for the barrier function of the epidermis^7,8^.

A cluster of genes encoding protein components of the cornified envelope of epidermal keratinocytes is known as the epidermal differentiation complex (EDC). The EDC is located on human chromosome 1q21.3, and homologous gene clusters have been identified in other mammalian species^9–12^ as well as non-mammalian tetrapods^13–18^. The EDC is bordered by genes of the S100A family, which are considered the evolutionarily ancestors of other EDC gene types^13,19^. Genes in the core region of the EDC can be classified into peptidoglycan recognition protein genes, single-coding-exon EDC (SEDC) genes and S100 fused-type protein (SFTP) genes. Peptidoglycan recognition protein 3 (*PGLYRP3*) and *PGLYRP4* genes have antimicrobial functions^20^ and differ substantially from other EDC genes^13^. SEDCs contain one protein-coding exon and one non-coding exon, whereas SFTPs comprise one non-coding and two protein-coding exons^13^. Gene families, such as small proline-rich proteins (*SPRRs*) and late-cornified envelope (*LCE*) genes, are crucial for the formation of the cornified envelope and belong to the SEDCs. Loricrin, involucrin, keratinocyte proline rich protein (*KPRP*), KPRP N-terminal and LCE C-terminal like protein (*KPLCE*), which was formerly known as *LEP7*, *XP32* or *C1orf68* (chromosome 1 open reading frame 68), proline rich 9 (*PRR9*) and late cornified envelope like proline rich 1 (*LELP1*) are SEDCs that exist as single-copy genes in the human genome.

SFTPs contain an S100 domain at their N-terminus and a long, sequence repeat-rich domain at the C-terminus^9,10^. Additionally, a short C-terminal sequence motif is conserved in most SFTPs^21^. Seven SFTP genes, i.e. cornulin (*CRNN*), filaggrin (*FLG*), filaggrin 2 (*FLG2*), hornerin (*HRNR*), repetin (*RPTN*), trichohyalin (*TCHH*) and trichohyalin-like1 (*TCHHL1*), are present in the human EDC. The best characterized genes among the latter are *FLG* and *TCHH*. FLG contributes to keratin filament aggregation in the epidermis, hydration of the stratum corneum and UV protection of the skin^22^. On histological sections, FLG forms, together with other proteins, basophilic keratohyalin granules in late differentiated but not yet cornified keratinocytes which form the granular layer of the epidermis. Mutations of the *FLG* gene are linked to skin barrier diseases, such as ichthyosis vulgaris and atopic dermatitis^23^. TCHH interacts with keratins and is expressed in the inner root sheath of the hair follicle, the tongue filiform papillae and the nail isthmus^24^.

Two clades of mammals have adapted to a fully aquatic lifestyle, cetaceans and sirenians. The former comprise whales, dolphins and porpoises and, together with artiodactyls, form the clade Cetartiodactyla within the superorder Laurasiatheria. Sirenians comprise manatees and dugongs and belong to the superorder of Afrotheria, with proboscideans (elephants) being their closest extant relatives^25^. Land-dwelling ancestors of cetaceans and sirenians independently underwent the evolutionary transition to life in the sea.

The skin of sirenians differs histologically from that of terrestrial mammals and shows some similarities to that of cetaceans, as it contains a subcutaneous fat layer called blubber and lacks sweat glands, and the epidermis is thicker than that of terrestrial mammals^26,27^ (Fig. 1). Furthermore, the epidermis lacks a granular layer and contains a thickened cornified layer of incompletely characterized structure in sirenians and cetaceans^27–29^. Specialized epithelial structures, namely vibrissae and keratinized pads that replace incisors have evolved as an adaptation of sirenians to feeding on seagrass^30^.

The genes encoding many epidermal proteins have been studied in detail in cetaceans, but only very incompletely in sirenians. Among EDC genes, *LOR*, *IVL*, *SPRR*s and *CRCT1* have been conserved in cetaceans, whereas *KPRP*, *KPLCE* and *LCE*s with the exception of *LCE7A* are absent in all cetaceans and *PRR9* and *LELP1* have been lost in subclades of cetaceans^11^. Keratins forming the cytoskeleton in the suprabasal epidermis of land-dwelling mammals, i.e. KRT1, KRT2, KRT9 and KRT10, are not conserved in cetaceans and they are also inactivated by mutations in the manatee^31,32^. Additional genes with functions in the epidermis were lost in cetaceans^33,34^.

In the present study, we analyzed the EDC of two species of sirenians in comparison to their homologs in humans and other mammals. We report that the coding sequence of the important skin barrier gene *FLG* is truncated and the FLG-processing protease, caspase-14, is inactivated by mutations in the dugong. However, we also demonstrate that most other EDC genes are conserved in sirenians and encode functional proteins, indicating roles of EDC genes that are not associated with the barrier to a dry environment.

## Results

### Identification of the EDC in the genomes of sirenians

**Figure 2:**
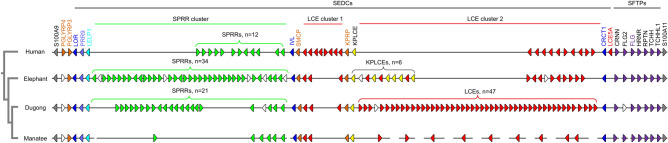
Comparison of the epidermal differentiation complex (EDC) in sirenians, elephant and human. The core region of EDC is represented by genes between *S100A9* to *S100A11*, which are schematically depicted as arrows pointing in the direction of transcription. Gene families are illustrated in identically colored arrows. White arrows indicate genes with a disrupted coding sequence by either premature stop codons or frameshifts. Gene family clusters are indicated by a bracket, where the “n” indicates the number of genes in the cluster. A cladogram shows the relation of the investigated species. Species: Human (*Homo sapiens*), elephant (*Elephas maximus indicus*), manatee (*Trichechus manatus latirostris*) and dugong (*Dugong dugon*). SEDC, simple EDC gene (1 coding exon); SFTP, S100 fused-type protein.

We investigated the EDC in the partly annotated genome sequence of the manatee and the not-yet-annotated genome sequence assembly of the dugong (Supplementary Tables S1, S2; Supplementary Figs. S1, S2). The gene organization of the EDC of sirenians was compared to the EDC in the Asian elephant (*Elephas maximus indicus*) (Supplementary Table S3; Supplementary Fig. S3), as a representative member of the phylogenetically closest terrestrial clade of mammals, the order Proboscidea. Furthermore, the human EDC was included in comparative analyses. The sequence of the EDC of the dugong was available as a continuous scaffold without sequence gaps, whereas genes of EDC of the manatee were identified on different sequence contigs that were not finally assembled at the time of this study (December 2023) (Fig. 2).

The EDC of both species of sirenians is comprised of S100A, PGLYRP, SEDC and SFTP genes in an arrangement homologous to that in other mammals^9–11^. We focused on the genes located between *S100A9* and *S100A11*. *PGLYRP3* is free of disruptive mutations, whereas *PGLYRP4* contains inactivating mutations in its coding sequence (Supplementary Fig. S4). Conservation of *PGLYRP3* and loss of functional *PGLYRP4* was also detected in the elephant, suggesting that the inactivation of *PGLYRP4* has occurred in a common ancestor of sirenians and elephants. Both intact genes and pseudogenes were also identified among the main types of EDC genes, that is, SEDCs and SFTPs, as will be described in detail below.

### Late cornified envelope (LCE) genes have been amplified in the dugong

Comparative analysis showed that sirenians have orthologs of all subtypes of SEDC genes (Fig. 2). *Loricrin*, *PRR9*, *LELP1*, *involucrin* (*IVL*), *SMCP*, *KPRP*, *KPLCE* and *CRCT1* are present as single copy genes in both manatee and dugong (Fig. 2, Supplementary Tables S1 and S2). Multiple paralogs of SPRRs and LCE genes are arranged in gene clusters in sirenians, similar to their homologs in elephants and humans. Due to gaps in the genome sequence of the manatee, the precise arrangement and the numbers of SPRR and LCE genes could not be determined for the manatee. In the dugong, twenty-one protein-coding SPRR genes and additional pseudogenized *SPRR*s are located between the *LELP1* and *IVL* genes. This number of SPRRs is smaller than that in the elephant (n = 34), but larger than the number of human SPRR genes (n = 12).

**Figure 3:**
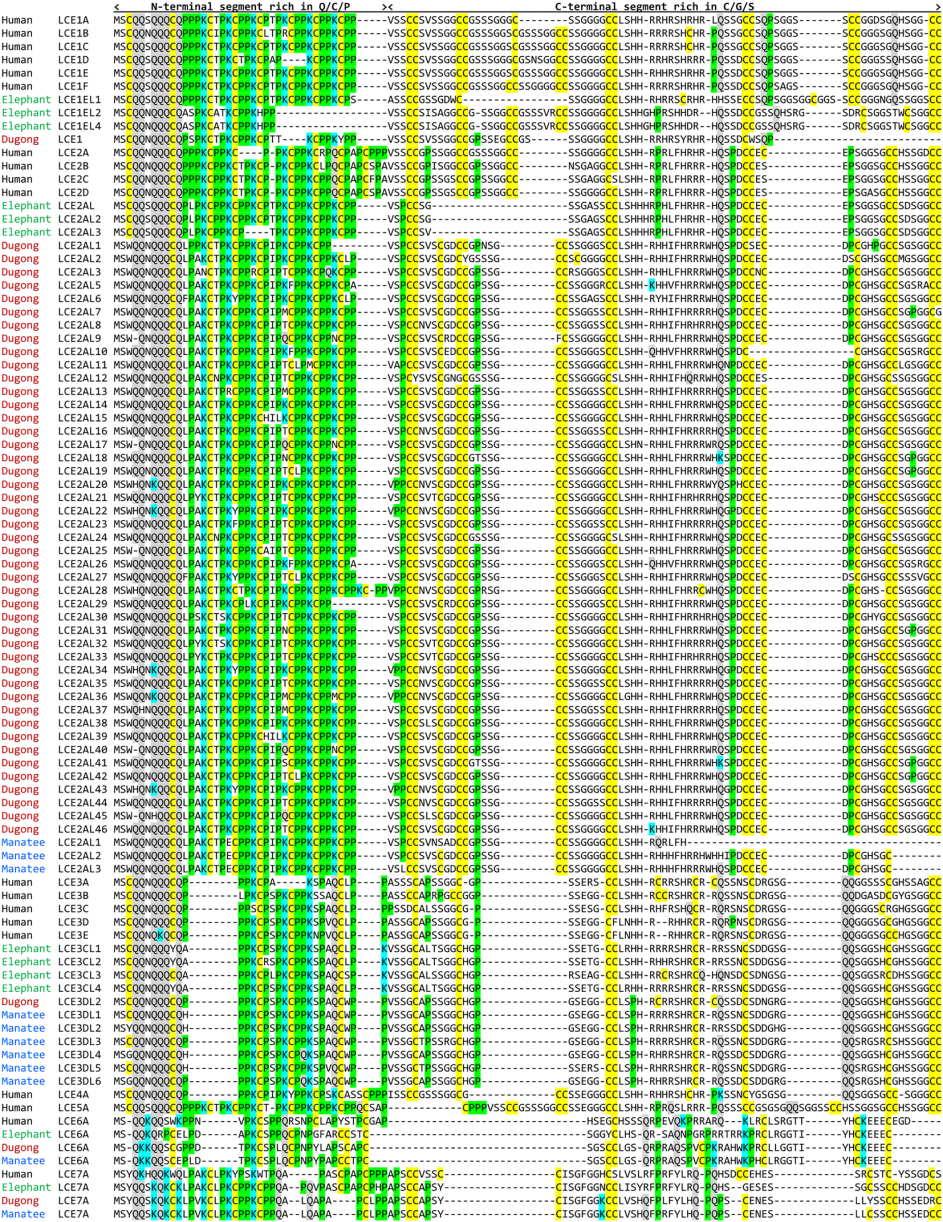
Amplification of late cornified envelope (LCE) proteins in dugong. Amino acid sequence alignment of LCE proteins in sirenans, elephant and human. Cysteine (C), glutamine (Q), lysine (K) and proline (P) are highlighted in yellow, grey, blue and green, respectively. Species: Human (*Homo sapiens*), elephant (*Elephas maximus indicus*), manatee (*Trichechus manatus latirostris*) and dugong (*Dugong dugon*).

Strikingly, the number of LCE genes is greatly increased in the dugong as compared to both elephant and humans. With 3 LCE genes in cluster 1 between *SMCP* and *KPRP* and 47 *LCE* genes in cluster 2 between *KPLCE* and *CRCT1*, the dugong has more than twice as many LCE genes as humans (n = 19) and the elephant (n = 15) (Figs. 2 and 3). The increase in the number of *LCE*s is due to the amplification of LCE2 paralogs which show slight variation at amino acid positions of the entire length of the protein (Fig. 3). Phylogenetic analysis confirmed that the main cluster of LCE genes of the dugong is monophyletic (Supplementary Fig. S5). Fewer LCE paralogs were identified in the genome of the manatee, which, however, contained several gaps in the region of the LCE genes (Fig. 2).

### Divergent evolution of KPLCE in elephants and sirenians

**Figure 4:**
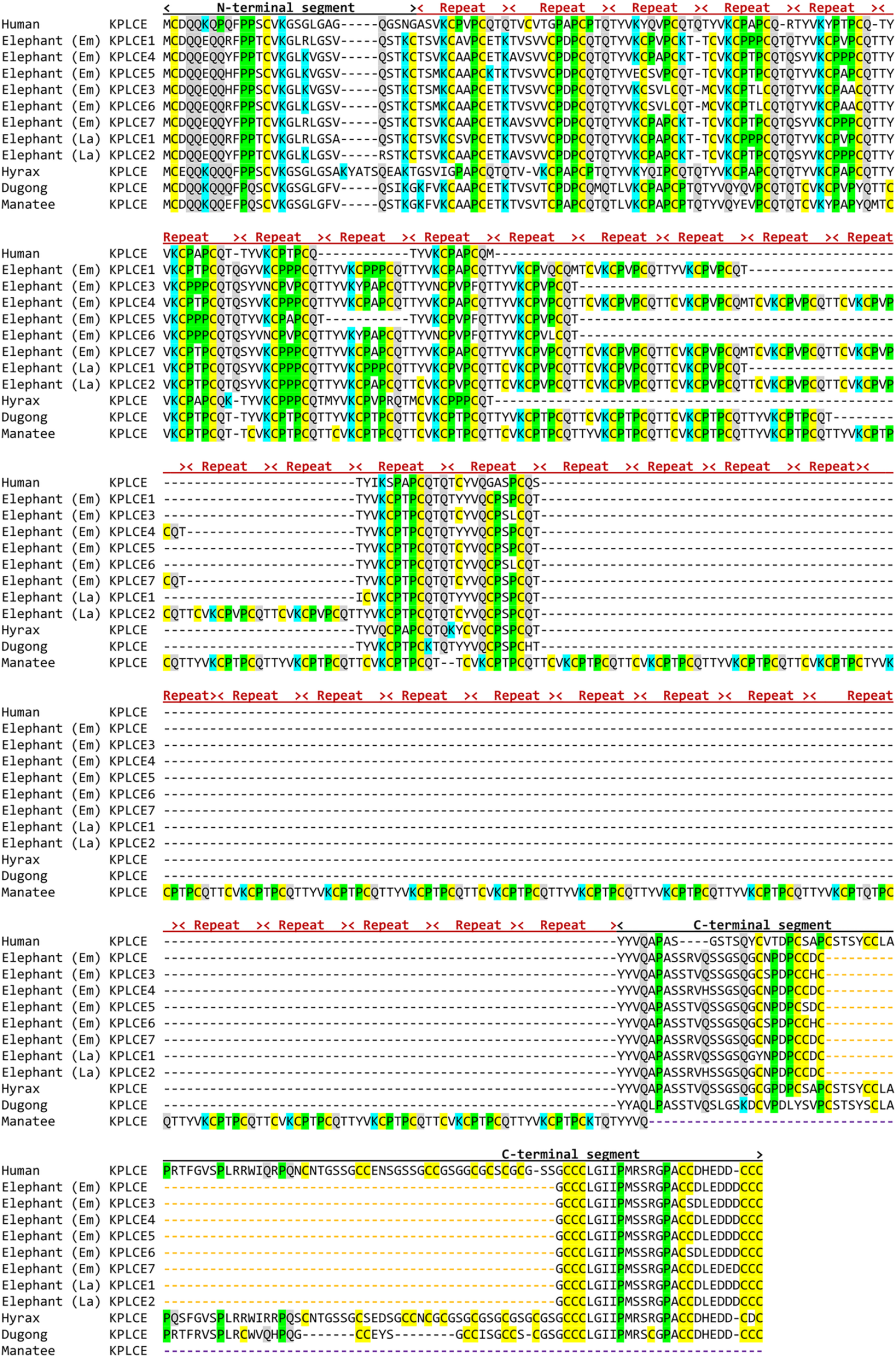
Amplification of KPLCE in elephants but not in sirenians and hyrax. Amino acid sequence alignment of KPLCE proteins of two elephant species compared to sirenians, hyrax and human. The positions of sequence repeats and the N-terminal and the C-terminal segments of the proteins are indicated above the alignment. Colored dashes indicate deletions in the C-terminal segment of KPLCE proteins in elephants and manatee. Cysteine (C), glutamine (Q), lysine (K) and proline (P) are highlighted by yellow, grey, blue and green shading, respectively. Species: Human (*Homo sapiens*), elephant (Em) (*Elephas maximus indicus*), elephant (La) (*Loxodonta africana*), hyrax (*Procavia capensis*), manatee (*Trichechus manatus latirostris*) and dugong (*Dugong dugon*).

*KPLCE* is a gene that has been recently re-named by GenBank after it was originally reported as *LEP7*, *XP32* or *C1orf68*. The KPLCE protein is characterized by a tripartite organization with an N-terminal segment, a central region with imperfect sequence repeats (Supplementary Fig. S6) and a C-terminal segment, which are largely conserved across species (Fig. 4). However, KPLCE of the manatee has an unusual organization as it contains more sequence repeats than its homologs in other species and lacks the C-terminal segment (Fig. 4). The EDC of the Asian elephant contains 7 copies of *KPLCE*, of which 6 encode proteins and one is a pseudogene (Fig. 2). The KPLCE proteins of the elephant are characterized by a shortened C-terminal segment, which lacks a subsegment of 59 amino acid residues present in human KPLCE (Fig. 4).

To estimate when in evolution the copies of *KPLCE* have emerged, we investigated the EDC of the African Savannah elephant (*Loxodonta africana*) and the rock hyrax (*Procavia capensis*). The African elephant has at least two intact copies of *KPLCE*, whereas the hyrax has only one (Fig. 4). This pattern suggests that the amplification of KPLCE has occurred in the phylogenetic lineage leading to elephants, and only one *KPLCE* gene was present in the common ancestor of sirenians, elephants and hyrax.

### Filaggrin and trichohyalin-like 1 genes contain premature stop codons in sirenians

**Figure 5:**
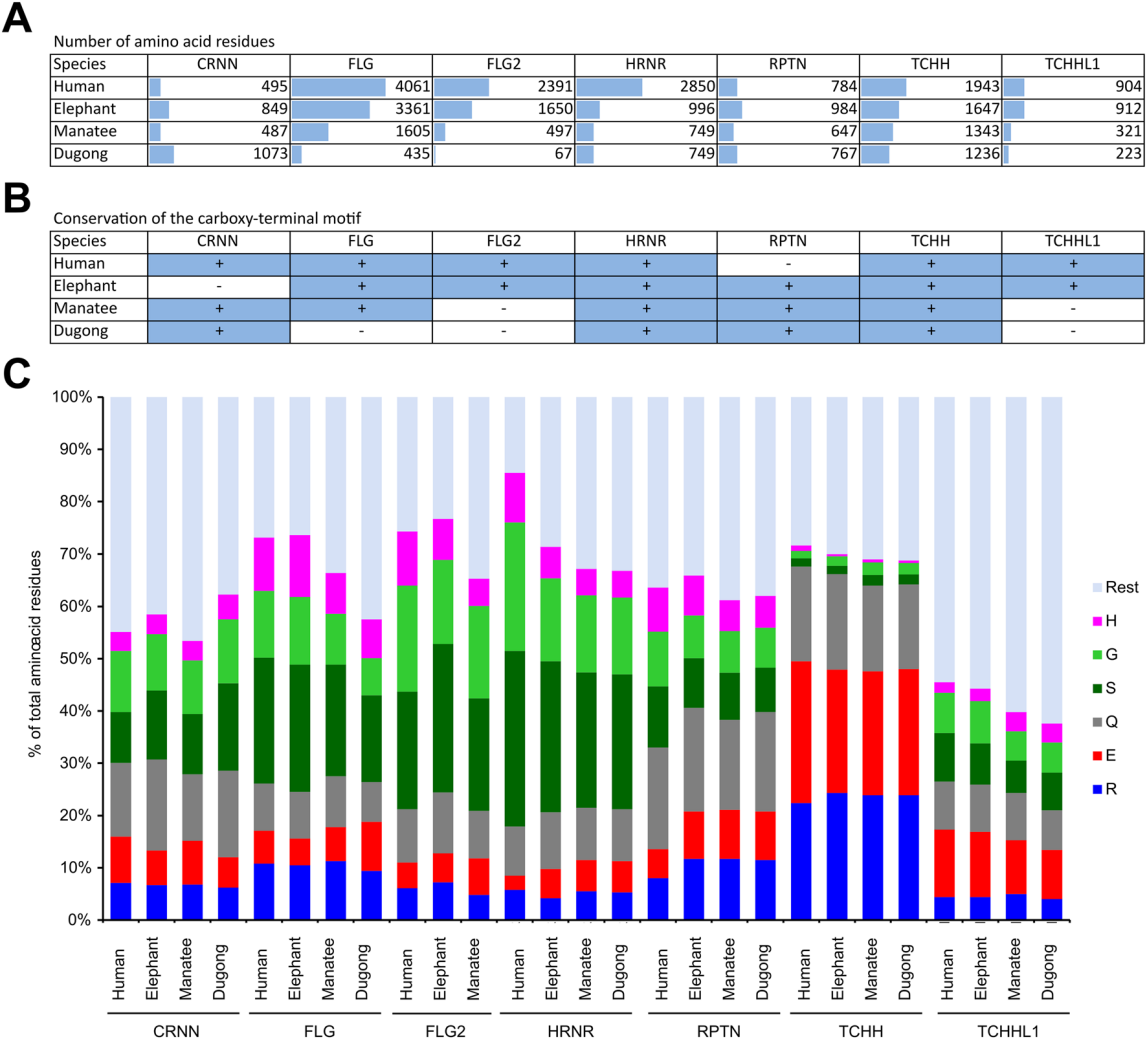
Comparison of SFTP genes of sirenians to placental mammals. Some SFTP genes of sirenians lack the carboxy-terminal motif and differ significantly in size compared to SFTPs in placentals. (**A**) Number of amino acid residues of sirenian SFTPs compared to SFTP proteins of elephant and human. (**B**) Comparison of the conservation of the carboxy-terminal motif in sirenians to placental mammals. (**C**) Amino acid contents of SFTP proteins of sirenians in comparison to elephant and human SFTPs. Alignments of the SFTPs are provided in Supplementary Fig. S7. Species: Human (*Homo sapiens*), elephant (*Elephas maximus indicus*), manatee (*Trichechus manatus latirostris*) and dugong (*Dugong dugon*).

SFTP genes form a cluster in the EDC of sirenians like in other mammals. All of the SFTP genes present in humans and elephants have homologs in sirenians (Fig. 2). However, due to premature stop codons the proteins encoded by *FLG*, *FLG2* and *TCHHL1* are more than 50% shorter in sirenians than in elephants and humans (Fig. 5A, Supplementary Fig. S7). A characteristic short amino sequence motif, that has been suggested to mediate binding of SFTPs to keratins^35^, is conserved in 6 out of 7 SFTPs of humans and elephants (Fig. 5B), but only in 4 and 5 proteins encoded by SFTP genes of the dugong and manatee, respectively. Both species of sirenians lack the C-terminal motif in the predicted FLG2 and TCHHL1 proteins (Fig. 5B). The C-terminal motif of SFTPs^21^ is present in FLG of the manatee but absent in FLG of the dugong. FLG2 of the dugong is predicted to be extremely short because of an in-frame stop codon in the currently available genome sequence. The sequence downstream of this predicted stop codon does not contain further stops for more than 2000 codons, suggesting that this gene has acquired the premature stop only recently in evolution.

SFTPs of sirenians and other species contain an N-terminal S100 domain of around 90 amino acid residues, followed by a long highly repetitive sequence that is strongly biased to only few amino acid residues. This leads to an extreme enrichment of few amino acids in many SFTPs. In line with this notion, only two amino acids, i.e. arginine (R) and glutamic acid (E), account for approximately 50% of all residues of TCHH in sirenians, strongly resembling TCHH in elephant and humans (Fig. 5C). Likewise, the high glycine and serine contents are conserved in HRNR of sirenians (Fig. 5C). Overall, the SFTPs of sirenians have a similar amino acid composition as their homologs in terrestrial mammals.

### Caspase-14 is inactivated by mutations in the dugong

**Figure 6:**
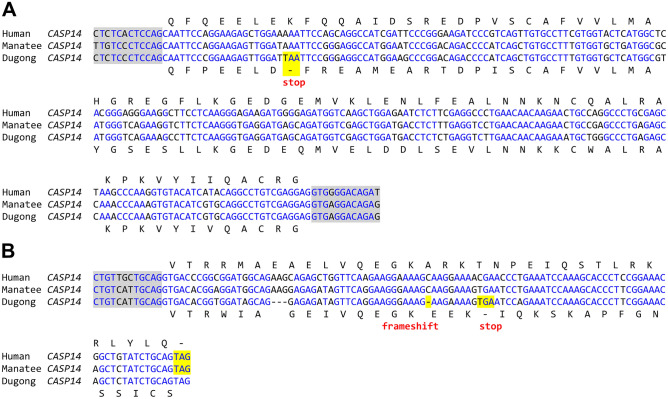
Mutations of *CASP14* in the dugong. Nucleotide sequence alignment of exon 4 (**A**) and exon 7 (**B**) of the *CASP14* gene in human (*Homo sapiens*), manatee (*Trichechus manatus latirostris*) and dugong (*Dugong dugon*) genomes. Intronic nucleotide sequences flanking the exons are marked by grey shading. Frame-shift mutations and premature stop codons are highlighted by yellow shading. Nucleotides conserved in all species are indicated by blue fonts. Amino acid sequences, derived by translation of exonic nucleotide sequences, are shown for human and dugong above and below the alignment, respectively. Nucleotide sequences in panel (**A**) Human (GenBank accession number NC_000019.10, nucleotides 15,053,721–15,053,970), manatee (GenBank accession number NW_004444058.1, nucleotides 8,025,835–8,025,586), dugong (GenBank accession number JASCZL010000003.1, nucleotides 19,502,339–19,502,451). Nucleotide sequences in panel (**B**) Human (GenBank accession number NC_000019.10, nucleotides 15,055,973–15,056,089), manatee (GenBank accession number NW_004444058.1, nucleotides 8,024,311–8,024,195), dugong (GenBank accession number JASCZL010000003.1, nucleotides19500820–19,501,069).

As FLG is an important skin barrier protein and mutations of the human *FLG* gene are associated with ichthyosis vulgaris and atopic dermatitis^36,37^, we investigated FLG-interacting proteins in the manatee, which has retained FLG, and the dugong, which has lost the C-terminal portion of FLG (Fig. 5A,B). Two proteases, aspartic peptidase retroviral like 1 (ASPRV1) and caspase-14 (CASP14), are expressed specifically in terminally differentiated keratinocytes where they are involved in the proteolytic processing of filaggrin^38,39^. ASPRV1 is conserved in the manatee and the dugong (Supplementary Fig. S8). By contrast, the *CASP14* gene is conserved only in the manatee, whereas it is disrupted by a premature stop codon and a frameshift mutation in the dugong (Fig. 6). All disruptive mutations of *CASP14* were present in three dugong genome sequences that were available in GenBank as results of independent projects (Supplementary Fig. S9).

## Discussion

**Figure 7:**
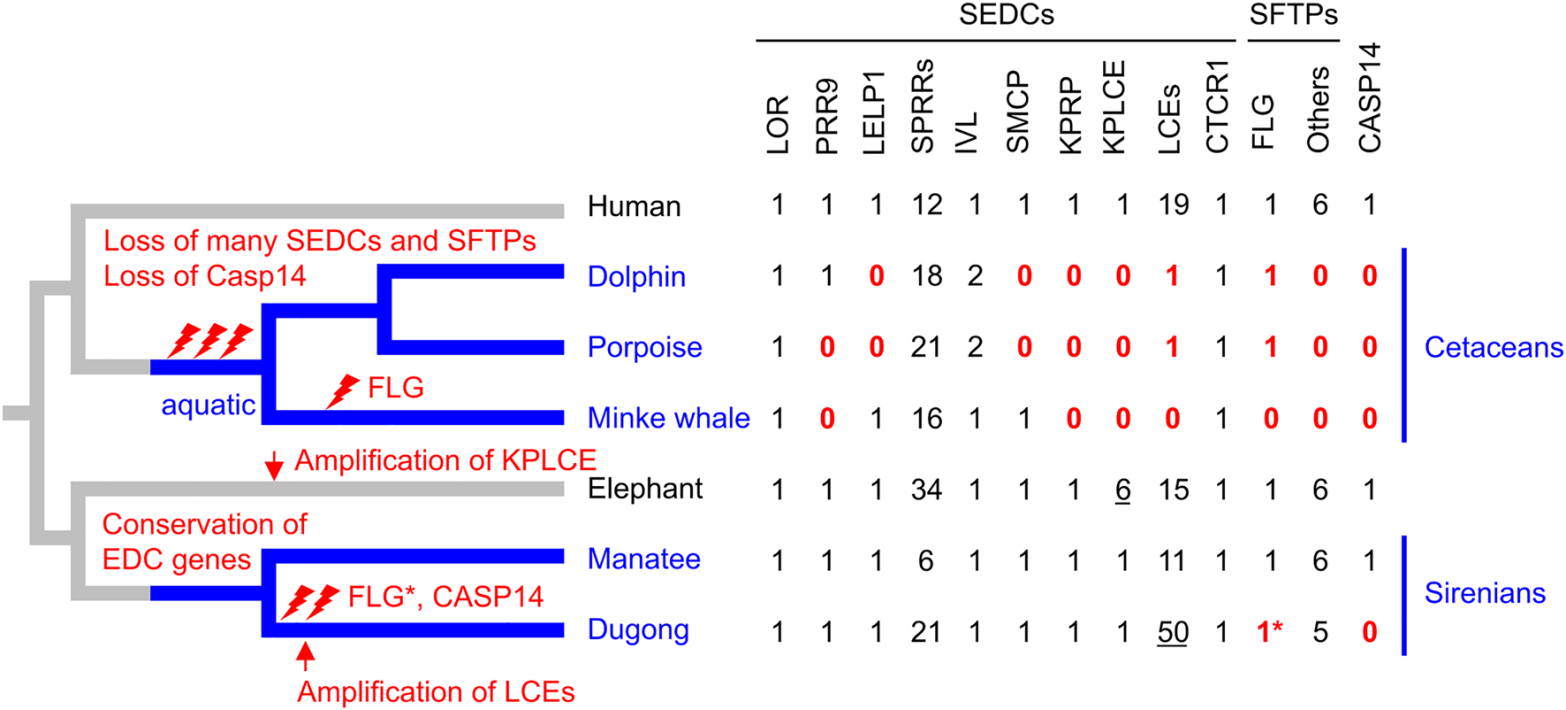
Schematic model showing the evolution of the EDC in mammals after the land-to-water transition. The matrix illustrates the numbers of genes belonging to the gene families in the species indicated. The cladogram shows the relation of the investigated species. Species colored in blue underwent the land to water transition. The asterisk indicates the loss of the C-terminal portion of FLG due to a premature stop. Underlines mark genes that are amplified in individual phylogenetic lineages. Species: Human (*Homo sapiens*), dolphin (*Tursiops truncatus*), porpoise (*Phocoena sinus*), Minke whale (*Balaenoptera acutorostrata scammoni*), elephant (*Elephas maximus indicus*), manatee (*Trichechus manatus latirostris*) and dugong (*Dugong dugon*).

The main function of keratinocyte differentiation is the establishment of the body’s interface with the environment^3,40^. Accordingly, adaptations to different environments are expected to involve adaptations of keratinocyte differentiation. Our results support this hypothesis with regard to mutations of genes, such as *FLG* and *CASP14*, implicated in the epidermal barrier formation in land-dwelling mammals. However, the extent of gene loss in the keratinocyte differentiation program is less pronounced than that in the other major group of aquatic mammals, the cetaceans^11,41,42^ (Fig. 7).

Sirenians have apparently intact *KPRP*, *KPLCE*, *PRR9*, *LELP1* and *LCEs*, the orthologs of which have been lost in cetaceans^11^. Our analysis shows that *LCE* genes are even amplified in the dugong, whereas the incompleteness of the current genome sequence assembly of the manatee does not allow to conclude on the number of LCE genes in this species. The increase of LCE gene copy numbers in the dugong has likely occurred through gene duplications by the mechanism of unequal crossing over^43^. The retention of the duplicated genes suggests that they have provided a selective advantage, for example by increasing the dosage of the encoded proteins or by facilitating subfunctionalization^44^. However, the possibility of neutral evolution of gene copy numbers needs to be considered^45^, and even potentially deleterious effects of large tandemly arrayed gene clusters have been discussed^46^. In humans, LCE proteins are components of cornified envelopes^47^. Their expression is increased upon exposure of the skin to ultraviolet radiation^48^ and during the repair of the skin barrier^49^, whereas lack of LCE3B and LCE3C due to gene loss predisposes to psoriasis^50^. LCEs have antimicrobial activities^51^ and interact with the antimicrobial cysteine-rich tail protein 1 (CYSRT1)^50^. It remains to be investigated which function of LCEs has been retained in sirenians whereas it is dispensable in cetaceans. Another antimicrobial protein encoded by an EDC gene, PGLYRP4, is absent in both sirenians and cetaceans^11^, indicating that this protein is dispensable for fully aquatic mammals.

In contrast to SEDC genes, the SFTP gene clusters of sirenians are affected by several mutations which are predicted to impair the normal function of the encoded proteins. The proteins encoded by the genes *FLG*, *FLG2* and *TCHHL1* are much shorter in sirenians than their orthologs in other mammalian species. Interestingly, the manatee has a potentially functional FLG including the characteristic C-terminal sequence motif of SFTPs (Fig. 5B), whereas FLG of the dugong is truncated and lacks this motif. Human *FLG* is probably the most-investigated EDC gene because polymorphisms of *FLG* affect skin barrier properties^21,52^ and *FLG* mutations are associated with the highly prevalent inflammatory skin disease, atopic dermatitis^37^. Both *FLG2* and *TCHHL1* are truncated by premature stop codons in sirenians (Fig. 5A,B). FLG2 is a component of cornified envelopes^53^ and mutations of the *FLG2* gene cause peeling skin syndrome type A^54^. *TCHHL1* is expressed in hair follicles^55^, and TCHHL1 protein was detected by mass spectrometry-based proteomics in mature hair shafts of mice^56^. As sirenians have a few hairs with putative mechanosensory functions, hair-related genes are not generally lost^31^. Accordingly, the main SFTP of the inner root sheath of hair follicles, TCHH, is conserved in both manatee and dugong. The comparison of SFTP genes in cetaceans^41^ and sirenians (this study) reveals striking differences, because all SFTPs have been lost in whales and only FLG is conserved in dolphins, whereas many SFTPs are conserved in sirenians.

Our finding of parallel loss of *FLG* and *CASP14* in the dugong suggests that a common pathway involving both proteins has been lost in the dugong. Caspase-14 is co-expressed with FLG^57^ and proteolytically processes FLG in murine and human keratinocytes^39,58^. However, *FLG* and *CASP14* have not been strictly interdependent during the evolution of mammals. *CASP14* is present in monotremes (platypus and echidna), whereas an SFTP with amino acid sequence features characteristic for FLG is missing^12^. *CASP14* has been lost in cetaceans, whereas *FLG* has been conserved, as mention above, in a subgroup of cetaceans^41^. Deletions in the human *CASP14* gene have been linked to a defect in cornification that manifests as autosomal recessive inherited ichthyosis^59^. The cellular features of the epidermis in manatees, which have *FLG* and *CASP14*, and dugongs, which lack *FLG* and *CASP14*, remain to be investigated in future studies.

Although the availability of genome sequences has provided insights into changes of keratinocyte differentiation genes, it is important to notice the limitations of the present study. First, the expression of EDC genes of sirenians remains to be investigated in situ, that is, in skin samples of manatees and dugongs. As protein sequences can be faithfully predicted now, proteomic analysis appears to be straightforward. Second, keratinocyte differentiation could not be studied in an in vitro model, because the culture of skin cells of sirenians is only in its infancy^60,61^, and fresh biosamples were not available to us. Finally, the interpretation of sequence data must be done cautiously because errors of DNA-sequencing and sequence assembly cannot be excluded.

## Material and methods

### Ethics statement

Genome and transcriptome data were obtained from public databases. This study involved neither humans nor animals.

### Identification of EDC genes in genomic sequences

Homologs of human EDC genes were identified by searches with the basic local alignment search tool (BLAST) at the NCBI website (https://blast.ncbi.nlm.nih.gov/Blast.cgi, last accessed on 21 December 2023) and analysis of the genomic region between the genes *S100A9* and *S100A11* in the genomes of the dugong (*Dugong dugon*, mDugDug1.hap1, GenBank accession number GCA_030035585.1, submitted by Vertebrate Genomes Project)^62^, manatee (*Trichechus manatus latirostris*, GenBank accession number GCA_030013775.1, submitted by Consejo Superior de Investigaciones Cientificas, Valencia, Spain) and elephant (*Elephas maximus indicus*, GenBank accession number GCF_024166365.1, submitted by Vertebrate Genomes Project). Sequences of the dugong that were considered important for the conclusions of this study, were analyzed in two additional dugong genome sequences (*Dugong dugon* assembly, WGS project CAJQER01, GenBank accession number GCA_905400935.1, submitted by Max-Planck Institute for Evolutionary Anthropology, Leipzig, Germany; *Dugong dugon* genome assembly D_dugong, WGS project BMBL01, GenBank accession number GCA_015147995.1, submitted by National Institute for Environmental Studies, Japan). The EDC region around *KPLCE* was analyzed in the genome sequence of another species of Proboscidea, the African savannah elephant (*Loxodonta africana*, GenBank accession number GCA_030014295.1, submitted by Vertebrate Genomes Project), and the hyrax (*Procavia capensis*, GenBank accession number GCA_000152225.2, submitted by Baylor College of Medicine, Houston, Texas). For some EDC genes, annotations were available in the genome sequence assemblies of NCBI GenBank, as indicated in Supplementary Tables S1–S3. Other EDC genes were identified by tBLASTn searches using proteins encoded in the EDC of humans or Afrotherian species as queries. To avoid false elimination of hits with biased amino acid composition characteristic for EDC proteins^13,24^, the filter for low sequence complexity was deactivated. Criteria for gene orthology were shared local synteny and reciprocal best hits in BLAST searches^63^.

### Analysis of amino acid sequences encoded by EDC genes

Amino acid sequences were aligned with MUSCLE^64^ and MultAlin^65^. The alignments were manually adjusted. Amino acid contents of proteins were calculated with the ProtParam tool at the ExPASy portal^66^. For the visualization of sequence repeats in KPLCE proteins, sequence logos were generated using the Weblogo software^67^.

### Molecular phylogenetics

Sequences belonging to the LCE family were collected from NCBI GenBank for each species of interest. The phylogenetic analysis was performed with PhyML (version 3.3.20220408)^68^ according to an approach described previously^19^. Phylogenetic trees were visualized and edited with FigTree (http://tree.bio.ed.ac.uk/software/figtree/, last accessed on December 17, 2023) and inkscape (version: 1.0.0.0; https://inkscape.org/de/, accessed on December 17, 2023).

### Supplementary Information

Supplementary Information.

## Data Availability

All data generated or analyzed during this study are included in this published article and its Supplementary Information files.
