# Supplementary material for: Comparative genomics of sirenians reveals evolution of filaggrin and caspase-14 upon adaptation of the epidermis to aquatic life
Source: Sci Rep. 2024 Apr 23;14:9278. doi: 10.1038/s41598-024-60099-2 (PMC11039687; doi:10.1038/s41598-024-60099-2)
Supplement: Supplementary file 1 — Supplementary Information. [file 41598_2024_60099_MOESM1_ESM.pdf]

Supplementary Data: Supplementary Tables and Figures

Comparative genomics of sirenians reveals evolution of filaggrin and caspase-14 upon adaptation of the epidermis to aquatic life

Julia Steinbinder, Attila Placido Sachslehner, Karin Brigit Holthaus, Leopold Eckhart

Content

Supplementary Tables S1-S3

Supplementary Figures S1-S9

Supplementary Table S1. EDC genes of the manatee (*Trichechus manatus latirostris*)

Gene name	Contig	Genomic DNA accession nr.	CDS start	CDS end	Sequence complete	Notes
S100A9	contig_9258_pilon	JARVKP010014720.1	97238	99584	yes	
PGLYRP4	contig_9258_pilon	JARVKP010014720.1	85949	66584	yes	pseudogene; frameshift mutation
PGLYRP3	contig_9258_pilon	JARVKP010014720.1	38709	16238	yes	
LOR	contig_18908_pilon	JARVKP010004035.1	26208	25718	yes	
PRR9	contig_18908_pilon	JARVKP010004035.1	55877	55527	yes	
LELP1	contig_18908_pilon	JARVKP010004035.1	68338	68045	yes	
SPRR2EL	contig_7965_pilon	JARVKP010007755.1	104348	103965	yes	
SPRR1B1	contig_7965_pilon	JARVKP010007755.1	90039	90452	yes	
SPRR1AL	contig_7965_pilon	JARVKP010007755.1	81171	81438	yes	
SPRR1AL2	contig_7965_pilon	JARVKP010007755.1	53757	54020	yes	
SPPR1B2	contig_7965_pilon	JARVKP010007755.1	42098	42415	yes	
SPRR4	contig_7965_pilon	JARVKP010007755.1	18570	18803	yes	
IVL	contig_15333_pilon	JARVKP010011006.1	44109	43012	yes	premature stop
SMCP	contig_15333_pilon	JARVKP010011006.1	61346	60990	yes	
LCE7A	contig_15333_pilon	JARVKP010011006.1	79836	79555	yes	
LCE6A	contig_15333_pilon	JARVKP010011006.1	94803	94573	yes	
KPRP	contig_15333_pilon	JARVKP010011006.1	209645	207771	yes	
KPLCE	contig_15333_pilon	JARVKP010011006.1	253516	252209	yes	
LCE2AL1	contig_16140_pilon	JARVKP010001178.1	3219	2986	yes	
LCE2AL2	contig_8786_pilon	JARVKP010010680.1	9064	8768	yes	
LCE2AL3	contig_6863_pilon	JARVKP010008136.1	177655	177951	yes	
LCE3DL1	contig_8786_pilon	JARVKP010010680.1	48477	48764	yes	
LCE3DL2	contig_21256_pilon	JARVKP010006131.1	10543	10830	yes	
LCE3DL3	contig_20385_pilon	JARVKP010005360.1	8265	8552	yes	
LCE3DL4	contig_13210_pilon	JARVKP010008709.1	7064	7351	yes	
LCE3DL5	contig_3327_pilon	JARVKP010009949.1	9554	9841	yes	
LCE3DL6	contig_3327_pilon	JARVKP010009949.1	2104	2391	yes	
CRCT1	contig_11079_pilon	JARVKP010012624.1	21881	21609	yes	
CRNN	contig_11079_pilon	JARVKP010012624.1	103249	105580	yes	
FLG2	contig_11079_pilon	JARVKP010012624.1	149165	151588	yes	
FLG	contig_11079_pilon	JARVKP010012624.1	208105	213516	yes	
HRNR	contig_11079_pilon	JARVKP010012624.1	262461	266427	yes	
RPTN	contig_21160_pilon	JARVKP010006059.1	59350	55861	yes	
TCHH	contig_21160_pilon	JARVKP010006059.1	21672	16929	yes	
TCHHL1	contig_7933_pilon	JARVKP010007730.1	302202	300393	yes	premature stop
S100A11	contig_7933_pilon	JARVKP010007730.1	234973	233326	yes	

Notes: CDS, coding sequence

Supplementary Table S2. EDC genes of the dugong (*Dugong dugon*)

Gene name	Genomic DNA accession nr.	CDS start	CDS end	Sequence complete	Notes
S100A9	JASCZL010000003.1	190566651	190564298	yes	
PGLYRP4	JASCZL010000003.1	190579014	190598369	yes	pseudogene; frameshift in exon 2
PGLYRP3	JASCZL010000003.1	190626496	190648979	yes	
LOR	JASCZL010000003.1	190691478	190690315	yes	
PRR9	JASCZL010000003.1	190794634	190794284	yes	
LELP1	JASCZL010000003.1	190807414	190807121	yes	
SPRR2EL1	JASCZL010000003.1	191906933	191907190	yes	
SPRR2EL2	JASCZL010000003.1	191928844	191929098	yes	
SPRR2EL3	JASCZL010000003.1	191966554	191966814	yes	
SPRR2EL4	JASCZL010000003.1	192032875	192033132	yes	
SPRR2EL5	JASCZL010000003.1	192062651	192062932	yes	
SPRR2EL6	JASCZL010000003.1	192133858	192134115	yes	
SPRR2EL7	JASCZL010000003.1	192163674	192163904	yes	
SPRR2EL8	JASCZL010000003.1	192182396	192182656	yes	
SPRR2EL9	JASCZL010000003.1	192285732	192285427	yes	
SPRR2EL10	JASCZL010000003.1	192315512	192315255	yes	
SPRR2EL11	JASCZL010000003.1	192387165	192386905	yes	
SPRR2EL12	JASCZL010000003.1	192405746	192405516	yes	
SPRR2EL13	JASCZL010000003.1	192435445	192435212	yes	
SPRR2EL14	JASCZL010000003.1	192532904	192532647	yes	
SPRR2EL15	JASCZL010000003.1	192552621	192552364	yes	
SPRR2EL16	JASCZL010000003.1	192572340	192572083	yes	
SPRR2EL17	JASCZL010000003.1	192636244	192636522	yes	
SPRR1B	JASCZL010000003.1	192657674	192657381	yes	
SPRR1AL1	JASCZL010000003.1	192666532	192666338	yes	premature stop
SPRR1AL2	JASCZL010000003.1	192693798	192693532	yes	
SPRR1B2	JASCZL010000003.1	192705413	192705120	yes	
SPRR4	JASCZL010000003.1	192728716	192728390	yes	
IVL	JASCZL010000003.1	193277578	193276649	yes	premature stop
SMCP	JASCZL010000003.1	193294758	193294423	yes	
LCE7A	JASCZL010000003.1	193313273	193312992	yes	
LCE6A	JASCZL010000003.1	193328283	193328053	yes	
LCE1	JASCZL010000003.1	193394777	193394508	yes	premature stop
KPRP	JASCZL010000003.1	193429186	193427450	yes	
KPLCE	JASCZL010000003.1	193471727	193470834	yes	
LCE2AL1	JASCZL010000003.1	193545913	193545605	yes	
LCE2AL2	JASCZL010000003.1	193564725	193564405	yes	
LCE2AL3	JASCZL010000003.1	193604175	193603855	yes	
LCE2AL4	JASCZL010000003.1	193628314	193628137	yes	pseudogene; frameshift
LCE2AL5	JASCZL010000003.1	193662118	193661798	yes	
LCE2AL6	JASCZL010000003.1	193735691	193735371	yes	
LCE2AL7	JASCZL010000003.1	193756864	193756544	yes	
LCE2AL8	JASCZL010000003.1	193774396	193774076	yes	
LCE2AL9	JASCZL010000003.1	193791931	193791614	yes	
LCE2AL10	JASCZL010000003.1	193815032	193814727	yes	
LCE2AL11	JASCZL010000003.1	193855825	193855505	yes	
LCE2AL12	JASCZL010000003.1	193897385	193897065	yes	
LCE2AL13	JASCZL010000003.1	193940036	193939715	yes	
LCE2AL14	JASCZL010000003.1	193957592	193957272	yes	
LCE2AL15	JASCZL010000003.1	193979823	193979503	yes	
LCE2AL16	JASCZL010000003.1	193995032	193994712	yes	
LCE2AL17	JASCZL010000003.1	194012574	194012257	yes	
LCE2AL18	JASCZL010000003.1	194028992	194028672	yes	
LCE2AL19	JASCZL010000003.1	194037164	194036844	yes	
LCE2AL20	JASCZL010000003.1	194058819	194058499	yes	
LCE2AL21	JASCZL010000003.1	194075300	194074980	yes	
LCE2AL22	JASCZL010000003.1	194099461	194099141	yes	
LCE2AL23	JASCZL010000003.1	194116859	194116539	yes	
LCE2AL24	JASCZL010000003.1	194162576	194162256	yes	

LCE2AL25	JASCZL010000003.1	194239056	194238739	yes	
LCE2AL26	JASCZL010000003.1	194262126	194261806	yes	
LCE2AL27	JASCZL010000003.1	194278702	194278382	yes	
LCE2AL28	JASCZL010000003.1	194308645	194308316	yes	
LCE2AL29	JASCZL010000003.1	194326073	194325765	yes	
LCE2AL30	JASCZL010000003.1	194343591	194343271	yes	
LCE2AL31	JASCZL010000003.1	194377343	194377023	yes	
LCE2AL32	JASCZL010000003.1	194394837	194394517	yes	
LCE2AL33	JASCZL010000003.1	194432460	194432140	yes	
LCE2AL34	JASCZL010000003.1	194456622	194456302	yes	
LCE2AL35	JASCZL010000003.1	194474034	194473714	yes	
LCE2AL36	JASCZL010000003.1	194490738	194490418	yes	
LCE2AL37	JASCZL010000003.1	194508137	194507817	yes	
LCE2AL38	JASCZL010000003.1	194525676	194525356	yes	
LCE2AL39	JASCZL010000003.1	194548161	194547841	yes	
LCE2AL40	JASCZL010000003.1	194579786	194579469	yes	
LCE2AL41	JASCZL010000003.1	194596167	194595847	yes	
LCE2AL42	JASCZL010000003.1	194604339	194604019	yes	
LCE2AL43	JASCZL010000003.1	194623982	194623662	yes	
LCE2AL44	JASCZL010000003.1	194641396	194641076	yes	
LCE2AL45	JASCZL010000003.1	194658896	194658579	yes	
LCE2AL46	JASCZL010000003.1	194682015	194681695	yes	
LCE3DL	JASCZL010000003.1	194738647	194739264	yes	
LCE3DL2	JASCZL010000003.1	194757574	194757861	yes	
CTCR1	JASCZL010000003.1	194790590	194790324	no	
CRNN	JASCZL010000003.1	194874289	194878382	no	
FLG2	JASCZL010000003.1	194887300	194888418	yes	pseudogene; premature stop
FLG	JASCZL010000003.1	194949378	194951282	yes	
HRNR	JASCZL010000003.1	195001694	195005663	yes	
RPTN	JASCZL010000003.1	195109010	195112732	yes	
TCHH	JASCZL010000003.1	195146923	195151324	yes	
TCHHL1	JASCZL010000003.1	195170993	195172507	yes	premature stop
S100A11	JASCZL010000003.1	195240398	195242055	yes	

Notes: CDS, coding sequence

Supplementary Table S3. EDC genes of the elephant (*Elephas maximus indicus*)

Gene name	Genomic DNA accession nr.	CDS start	CDS end	Sequence complete	Genbank Gene ID	Notes
S100A9	NC_064821.1	189457864	189460228	yes	126073624	
PGLYRP4	NC_064821.1	189435912	189416644	yes	126073622	pseudogene
PGLYRP3	NC_064821.1	189392580	189358003	yes	126073623	
LOR	NC_064821.1	189308232	189308978	yes	126071457	
PRR9	NC_064821.1	189264530	189264880	yes	126073625	
LELP1	NC_064821.1	189252129	189252434	yes	126073628	
SPRR1L	NC_064821.1	189207956	189208177	yes	126073649	
SPRR1L2	NC_064821.1	189169769	189169972	yes	n.a.	premature stop; not predicted in Genbank
SPRR2EL	NC_064821.1	188914878	188914669	yes	n.a.	not predicted in Genbank
SPRR1AL	NC_064821.1	189118309	189117986	yes	n.a.	not predicted in Genbank
SPRR2EL2	NC_064821.1	189105988	189105740	yes	n.a.	not predicted in Genbank
SPRR2BL	NC_064821.1	189105950	189105750	yes	n.a.	not predicted in Genbank
SPRR2EL3	NC_064821.1	189085965	189085639	yes	n.a.	not predicted in Genbank
SPRR2EL4	NC_064821.1	189059226	189059017	yes	n.a.	not predicted in Genbank
SPRR2EL5	NC_064821.1	188977152	188977361	yes	n.a.	not predicted in Genbank
SPRR2EL6	NC_064821.1	188884140	188884349	yes	n.a.	not predicted in Genbank
SPRR2EL7	NC_064821.1	188786068	188785859	yes	n.a.	not predicted in Genbank
SPRR2EL8	NC_064821.1	188745309	188745040	yes	126073657	
SPRR2EL9	NC_064821.1	188718389	188718180	yes	126073656	differently predicted in Genbank
SPRR2EL10	NC_064821.1	188659856	188659617	yes	n.a.	not predicted in Genbank
SPRR2EL11	NC_064821.1	188518349	188517981	yes	126071454	pseudogene; premature stop
SPRR2HL	NC_064821.1	188442705	188442406	yes	126071452	
SPRR2EL12	NC_064821.1	188397198	188396965	yes	126073640	
SPRR2GL	NC_064821.1	188376926	188377132	yes	126073655	
SPRR2EL13	NC_064821.1	188352774	188352568	yes	126073654	
SPRR2GL2	NC_064821.1	188338620	188338387	yes	126071451	
SPRR2EL14	NC_064821.1	188318236	188318030	yes	126071450	
SPRR2GL3	NC_064821.1	188294576	188294343	yes	126073645	
SPRR2EL15	NC_064821.1	188259957	188259751	yes	n.a.	not predicted in Genbank
SPRR2GL4	NC_064821.1	188237540	188237334	yes	126073653	
SPRR2GL5	NC_064821.1	188223469	188223263	yes	126073652	
SPRR2EL16	NC_064821.1	188203172	188202834	yes	n.a.	not predicted in Genbank
SPRR2GL6	NC_064821.1	188172624	188172391	yes	126073644	
SPRR2GL7	NC_064821.1	188158435	188158202	yes	126073643	
SPRR2EL17	NC_064821.1	188138118	188137912	yes	n.a.	not predicted in Genbank
SPRR2GL8	NC_064821.1	188115656	188115423	yes	126073642	
SPRR2EL18	NC_064821.1	188100879	188100673	yes	126073651	
SPRR2EL19	NC_064821.1	188051676	188051437	yes	126073639	premature stop
SPRR2EL20	NC_064821.1	188005693	188005460	yes	126073641	
SPRR1BL	NC_064821.1	187988093	187988314	yes	126073647	
SPRR1AL2	NC_064821.1	187979556	187979814	yes	126073638	frame-shift mutation
SPRR1AL3	NC_064821.1	187953799	187954065	yes	126073637	
SPRR1AL4	NC_064821.1	187938358	187938624	yes	n.a.	not predicted in Genbank
SPRR1B	NC_064821.1	187926517	187926786	yes	126073635	
SPRR4	NC_064821.1	187910590	187910799	yes	126073650	pseudogene; premature stop
IVL	NC_064821.1	187844192	187845871	yes	126071449	differently predicted in GenBank
SMCP	NC_064821.1	187827400	187827798	yes	n.a.	not predicted in Genbank
LCE7A	NC_064821.1	187804688	187804981	yes	126071448	
LCE6A	NC_064821.1	187784192	187784422	yes	126073646	
LCE1EL	NC_064821.1	187457892	187458218	yes	126071447	
KPRP	NC_064821.1	187438273	187439748	yes	126073621	differently predicted in GenBank
KPLCE	NC_064821.1	187391807	187392538	yes	126071446	
KPLCE2	NC_064821.1	187330346	187330543	yes	126071445	pseudogene; premature stop
LCE1EL2	NC_064821.1	187318415	187318735	yes	n.a.	not predicted in Genbank
KPLCE3	NC_064821.1	187280043	187280708	yes	126071444	
LCE1EL3	NC_064821.1	187268123	187268464	yes	n.a.	not predicted in Genbank
KPLCE4	NC_064821.1	187230846	187231643	yes	126071443	
KPLCE5	NC_064821.1	187180621	187181253	yes	126071441	
KPLCE6	NC_064821.1	187130404	187131069	yes	126071440	

LCE1EL4	NC_064821.1	187118486	187118806	yes	n.a.	not predicted in Genbank
KPLCE7	NC_064821.1	187079938	187080735	yes	n.a.	not predicted in Genbank
LCE2AL1	NC_064821.1	187026915	187026634	yes	126073631	
LCE2AL2	NC_064821.1	187017414	187017695	yes	126073630	
LCE2AL3	NC_064821.1	187003197	187003466	yes	126073636	
LCE2AL4	NC_064821.1	186971752	186971591	yes	126073632	
LCE3CL	NC_064821.1	186937652	186937363	yes	126071768	
LCE3CL2	NC_064821.1	186932242	186932529	yes	126071767	
LCE3CL3	NC_064821.1	186920072	186919785	yes	126071766	
LCE3CL4	NC_064821.1	186911755	186911468	yes	126071765	
LCE2AL5	NC_064821.1	186905665	186905405	yes	n.a.	not predicted in Genbank
LCE3CL5	NC_064821.1	186885051	186884749	yes	126071439	
CRCT1	NC_064821.1	186832868	186833158	yes	n.a.	not predicted in Genbank
CRNN	NC_064821.1	186725738	186721798	yes	126071438	premature stop
FLG2	NC_064821.1	186668896	186663034	yes	126071437	predicted as *FLG* in GenBank
FLG	NC_064821.1	186549798	186539082	yes	n.a.	not predicted in GenBank
HRNR	NC_064821.1	186472276	186467542	yes	126071436	differently predicted in GenBank
RPTN	NC_064821.1	186372196	186368421	yes	126071435	differently predicted in GenBank
TCHH	NC_064821.1	186340042	186334190	yes	126071434	differently predicted in GenBank
TCHHL1	NC_064821.1	186311087	186307428	yes	126073614	differently predicted in GenBank
S100A11	NC_064821.1	186232551	186227394	yes	126073618	

Notes: GenBank ID indicates the most similar gene prediction in GenBank. The presence of differences between these GenBank predictions and the gene predictions in this study is indicated in the last column. CDS, coding sequence; n. a., not applicable

A

>Tml_LOR
MSHQKKQPTPIPPVGCVKIGGGGGGGCGGGGSGGGGDSSCGGGSSGGGSGGGIKYCGDGGSSGGGSSCGGGSSGGFGGSHSGQ
QVQYQSYGGGCSGGSSGGGSGCFSSGGGGDGGSCYGGGSSRGGGDSSCGGGSSRGGSGSGKGVPVCHQTQQKQAPTWPCK

>Tml_PRR9
MSFDEQQCKQPCVPPPCLQNTQEQCQAKAEEVCLPPGQDPCQEKCLAQVQEVGLPQCQELSQENRPQQGLDPCLPPCQDQCLP
QCVEPCQELSQTKCVEIFPQKSQEKCSPPGKGK

>LELP1
MSSDDKSKSGEQKTEPKCEQKCEPKCEQKCESKCQPSCLKKLLQRCSDKCPRDKCPSPCPPKCPPPCPPPCPPPCPPPKPCTK
PCPPKCPSLCPPPE

>Tml_SPRR2EL
MSYQQQQCKQPCQPPPVVCPPKCPEPCPPKCPEPCPPPKRPEPCPPKCPEPCPPPKCPEPCPPKCPEPCPPPKCPEPCPPPKC
PEPCPPVQCPPQPCQQKCPPVQVFQPCQQKCPPKSKNLQDSSGS

>Tml_SPRR1B1
MSSQQQKKPCTLTPQLQQQQVKQPCQPPPQEPRVPQTKEPCHPKVPKPCHPTVPEPCHPKVPEPCHPTVPEPCHPKVPEPCHP
KVPEPCHPTVPEPCHPKVPEPCHPKVPEPCHPKVPEPCPSKVTPAPAQQKTKQK

>Tml_SPRR1AL
MSSYQQKQPFTPPPQPHGQQVIQACLPPQEPPVPITKAPRHSQVPPPGNTKVPEPGYPNVPDPVYYKDPQSCTSPFTPGPGQQ
KTKQN

>Tml_SPRR1AL2
MSSYQQKQPFTPPPQPHEQQVIQPCLPPQKPPVPVTKTPGHPQVPPPGNTKVPEPGYPKVPDSVCRKDPEPCPSPVSPDPDQQ
KTKKN

>Tml_SPRR1B2
MNSQQQKKPCVLPPQLQQQQVKQPCQPPPQEPCVPKTKEPCHPKVPEPCHPTVPEPCHPTVPEPCHPVVPEPCHPTVPEPCHP
KVPEPCPSKVTPAPAQQKTKQK

>Tml_SPRR4
MSSQQQEQQQCPPQKPQQQQVKQPCQPPPVKCQETCAPQTKDPCASQATKQCSPKGTATAVQQKCPPAQQAPKSKQK

>Tml_IVL
MSQQYKLPVTLPADLCQPPLKTVSPPVDTQQEQVKQPAPLPSPGQKVASEPPVKVPSEQGEKHTAPVKEVPEQELGQQQQPQE
QEQHKEQQHKEPQKLEQHQDQQHQKPQKLEQQQEQQHQEPQELEQEQEQQHQEPQELEQHQDQQHQEPQELEQHQDQQHQEPQ
ELEQQQDQQHQEPQELEQEQEQQHQEPQELEQHQDQQHQEPQELEQHQDQQQQEPQELEQHQDQQQQEPQELEQHQDQQQQEP
QELEQHEEQQQQQPQEPQEPQEQVQHQEQQQQPQEQQKQEHQHQEQHQEHQESESLEQQPKQEKAQKEEQLKEQLDQKKKLLD
HQIDQELEKKDEQLGKKEEQLPEHLEHQKQQSL

>Tml_SMCP
MCDQPKRNPCCQPPRQCCPSPRQCCPPPRQCCLPPRQCCPLPRQCCPPPRQCCPPPRQCCPSAPPCCIKQQCLEAKAECTCLN
EESEPKPLQTQDRGSHSPQNAPRPGILGQQKKSSK

>Tml_LCE7A
MSYQQSKQKCKLPVKCLPKCPPKCPPQALQAPAPCLPPAPSCCAPSYCISGFGGKCCLVSHQFPRFYLHQPQPSCENESLLCS
SCCHSSEDCC

>Tml_LCE6A
MSQKKQQSCEPLDTPKCSPLQCPNPYPAPCCTPCSGSCCLGSQRPRAQSPVCPKRAHWKPHCLRGGTIYHCKEEEC

>Tml_KPRP
MCDQPLIPSCLPLPQSCVKGSSFFPSPSPCATGQVVVQAPCEMQIVECPAPCPVQVSQVKCVAPCQSKTTQVKGQAPCQTQTT
QVESQAACQSEVSYVQCEVPCPVQTCYIEHAPVCCRETCYVEYPVQTCVTCPVPQPVQTYVTYSPVCQNQGRFSVQGQYQGSY
SRCTPQSQSRASFSTCAPQCQTQGSYGSFTALRRSQSARRCLPFSQLESSYRSCSLPRGLGCYYSICLPSRCCSDSCYCTPPR
RSEPIYNSGCSRSRTSGRSQRCGPKCRVEISSPCCPKQVPPQKCSVQIPPIGRCSKICAPPFSCRASCPDLRPCQLQSSSFRP
PRRRDQCPEPLLLPCPLPEPRPYPRPCPLPEPRPYPRPCPLPEPLPYPRPCPLPEPRPYPRPCPLPEPLPYPRPCPLPEPRPY
PRPCPLPEPRPYPRPCPLPEPLPYPRPCPLPEPRPHPRPCPLPEPRPYPRPCPLPEPLPYPRPCPLPEPLPCPSPCPCPERCL
SPEPCPCPPPRWSSEPCLYPEPRPAPSPAPRQALPSRPVWHEFPDRHLRAQPCDLPEPRPLPEPIPLPEPCPSPEPCVEPQSY
PNPCSGPKPIPCPPDLGCQESSPFRLDSEVPSCGPGGYNQGRE

>Tml_KPLCE
MCDQQKQQEFPQSCVKGSGLGFVQSTKGKFVKCAAPCETKTVSVTCPAPCQTQTLVKCPAPCPTQTYVQYEVPCQTQTCVKYP
APYQMTCVKCPTPCQTTCVKCPTPCQTTCVKCPTPCQTTCVKCPTPCQTTCVKCPTPCQTTYVKCPTPCQTTCVKCPTPCQTT

YVKCPTPCQTTYVKCPTPCQTTYVKCPTPCQTTYVKCPTPCQTTCVKCPTPCQTTCVKCPTPCQTTCVKCPTPCQTTCVKCPT
PCQTTYVKCPTPCQTTCVKCPTPCQTTYVKCPTPCQTTCVKCPTPCQTTYVKCPTPCQTTYVKCPTPCQTTCVKCPTPCQTTY
VKCPTPCQTTYVKCPTPCQTTYVKCPTPCQTTYVKCPTPCQTTYVKCPTPCQTTCVKCPTPCQTTYVKCPTPCQTTCVKCPTP
CQTTYVKCPTPCKTQTYYVQ

>Tml_LCE2AL1
MSWQQNQQQCQLPAKCTPECPPKCPIPKCPPKCPPKCPPVSPCCSVNSADCCGPSSGCCSSGGGGCCLSHHRQRLFH

>Tml_LCE2AL2
MSWQQNQQQCQLPAKCTPECPPKCPIPKCPPKCPPKCPPVSPCCSVSCGDCCGPSSGCCSSGGGGCCLSHHRHHHFHRRHWHH
IPDCCECDPCGHSGC

>Tml_LCE2AL3
MSWQQNQQQCQLPAKCTPECPPKCPIPKCPPKCPPKCPPVSLCCSVSCGDCCGPSSGCCSSGGGGCCLSHHRHHHFHRRRWHQ
SPDCCECDPCGHSGC

>Tml_LCE3DL1
MSCQQNQQQCQHPPKCPSPKCPPKSPAQCWPPVSSGCAPSSGGCHGPGSEGGCCLSPHRRRRSHRCRRQSSNCSDDGRGQQSG
GSHCGHSSGGCC

>Tml_LCE3DL2
MSYQQNQQQCQHPPKCPSPKCPPKSPAQCWPPVSSGCAPSSGGCHGPGSEGGCCLSPHRRHRSHRCRRRSSDCSDDGHGQQSG
GSHCGHSSGGCC

>Tml_LCE3DL3
MSCQQNQQQCQPPPKCPSPKCPPKSPAQCWPPVSSGCTPSSRGCHGPGSEGGCCLSPHRRRRSHRCRRQSSNCSDDGRGQQSR
GSRCSHSSGGCC

>Tml_LCE3DL4
MSGQQNQQQCQHPPKCPSPKCPQKSPAQCWPPVSSGCAPSSGGCHGPGSEGGCCLSPHRHRRSHRCRRQSSDCSDDGRGQQSG
GSHCGHSSGGCC

>Tml_LCE3DL5
MSCQQNQQQCQHPPKCPSPKCPPKSPVQCWPPVSSGCTPSSGGCHGPSSERSCCLSPHRHRRSHRCRRQSSNCSDDGRGQQSR
GSHCGHSSGGCC

>Tml_LCE3DL6
MSGQQNQQQCQPPPKCPSPKCPQKSPAQCWPPVSSGCAPSSGGCHGPGSEGGCCLSPHRHRRSHRCRRQSSDCSDDGRGQQSR
GSHCGHSSGGCC

>Tml_CTCR1
MSSQQTSKGFSKGTSKGPAPCPAPTPAPASSSSSGCGHGGCGSSSCCGDSCCCNPGCCGSSSASCCCFPRRRSRQRRCCCCCG
GSQRSQC

B

>Tml_CRNN
MQRKDCEAETRPSKQRRLMWSPPRGLFIFSSLTSKMPQLLRNIHGIIEAFGRYAKTEGNCTVLTRGELKRLLEHEFADVIVKP
HDPATVDEVLRLLDEDNTGTVEFKEFLVLVFKVAQACFKTLSESPEGACGSQESRSHHPGASQELREGQRSHTEVGWAGRGQL
REGSSQGQSKQASLKHDGSSAQVSLHDKQVESQRQESPSQQAQETGHTEHTQTAQDQRHQTRVGWSERQSQTREQSRAHQTSE
TVTGTLTQTQAGTTQTLGQDRSCQMGSTSTQSQESTCGQTGGTHSQDRSRQTGSTSTQSQESTCGQTGGTETNGQDRNQTSQV
VTGHIQTQAGSQTQVHPQTVEQDRSQTGRHAGAREEGQSQTQSGSGQTWTRVSNYEAGETVLEGQPQPGAGTLPGSQDGSSMH
PSYSVTGEQGESNVVVEEWVDDHTREMVIQRQDQGTLPSSASSALGGETAQPKERRGITAKGLYSYFQSTKP

>Tml_FLG2
MTDLLRSVVTVIDIFHKYTKQDGECATLSKDELKELLEKEFHPILKNPDDTDTVDVNMHMLDQDHDRQLDFTEFLLMVFKLAM
ACNKALSKEYCKASGSKKCRHGHQHQEEESETEEEEEETQGWKSGYRYSSWNEREEHQYGSAGSRGTMKHRLRSNSRRLGRQG
GLSSSENEEGSEKMCHGSSSGHSWSSGKERHGSSSEELGERRNKLSVSSCGESGEEYECGSGSKNGGRKGHGGLSLELEASGH
ESSSIQSRSGGQRLGSIFGGSGDCGRQSHACNSSNSGGCGSPQNASSSCQAGRFGGQGNQSSCTQSGYQSGSSGGQGHGCISG
GQSSGYSQHNSGSYSQCSSHRGHGSRACGQPQNCRGQQQGTGSSQSSCCGQYGSGASQSSSYGQQGYGSCGHSFTSSQKRSGS
NMFSKCGQCGSGSGQSSSQHRSSSSQSSGFGQHGSGTGQSSGQHGSGSSQFSGQHGSGTGQSSGQHGSGSSQFSGQHGSGTG

>Tml_FLG
MSILLENIVAIIDLFQQYSSKDKENDTLSEKELTELLEREFRSILKHPDDPDTTDVFMHFLDVDDNEKINFTEFLLMVFKLAQ
AYYESMKRKNFKATSTKQKKHGQERQNEEYEIEEEEKEEEQRRESILSSRHGRRGKRSPIRTDKKKHGSISNSKRRRDSSSPF
GHREKNERKHHHHTGKYYNRSSSTDLGEERNTSSIGQKREQEGDEHDYGYEKRGRVSDTQEDASQEESAHSGRQSGSSSRNHH
GSIHGQARDSSRHSRSLEQQTADRAQSHSGHVHSDSTATGRQGSIHGQSVDSARHSGSHQGERSTGVQSRSSTRGRQEFHQDH
SADPSRDSQSGCGKSSSEFRGDTHRGSSVSQTRDSEGQSEDSESQSGSSSRNHYGSTHGQATDSSRHSRSHEQQRSDHAQSGS

AHGHSDSTAIGRQGATYGQSVDSSRHSGSHQGERSAGVQSRSSARGRQDFHQDQSADRSTDSRSGRGQSSSELGARTHRGSVV
SQASDSGQQSEDSERQSASSSRNHHGFTQDLARDSFRHSRSLEQQTADRAQSHCGHGYSDSTATGRQGSIHGETVDNSRHSGS
HQGERSTGVQLRLTTRARQELHQDQSADRSRDSRSGRGQSSSELGTSTHRGSSVSQARDSEGQSEDSERQSGSSSRNHYGSTH
SQARDSSRHSRSHQVQRADRAWSGSAHAHSDSTATGRKECIHGETVDTSRHSGSHQGEWSACVQLRSTTRARQELHQDQSADR
STDSRSGRAQSSSELGGRTHRGSVVSQASDCEGQSEDSERQPASSSRNHHGFTQDQARDSFRHSRSHEQQRVNGAQSHSGHGH
SDSTATGRQGFIHGETVDNSRHSGSHQGERSTGVQSRSTTRGRQEFHQDHSADPSRDSQSGQSSSEFRADTHRGSSVSQASDS
EGQSEDSERQSGSSSRNHYGSTHSQARDSSRHSRSHQVQRADRAWSGSAHAHSDSTATGRKECIHGETVDTSRHSGSHQGERS
ACVQLRSTTRARQELHQDQSADPSTDSRSGRAQSSSELGGRTHRGSVVSQASDCEGQSEDSERQPASSSRNHHGFTQDQARDS
FRHSRSHEQQRVNGAQSHSGHGHSDSTATGRQGSIHGETVDNSRHSGSHQGERSTGVQSRSTTRGRQEFHQDHSADPSRDSQS
GQSSSEFRADTHRGSSVSQASDSEGQSEDSERQSGSSSRNHYGSTHGQARDSSRHSRSHEQQRSDHAQSGSAHGHSDSTARGR
QGATYGQSVDTSRHSGSHQGERSAGVQSRSSTRARQELHQDQSADRSTDSRSGRAQSPSELGGRTHRGSVVSQASDCQGQSED
SERQSASSSRNHYGFTQDQARDSFRHSRSHEQQRSDHAQSGSAHGHSDSTATRRKGSTRGQSVDSSRHSASHHGERSAGVQSR
SSTRGRQELHQDQSADRSRDSWSGLGQSSSEFRASTHRGSSVIQASDNEGQSEESARQSASNYETSGSSSRKQAESTHGQSGE
HQRRSGAHQGQKSTHGESDSTQEHQRQKADHGQADSNDTKSEGSSLSRFHSVNNDRRSHGAGQCWRHGSYGSTDYDYGQSGFG
QSQYGIVRNDRSGTKQVSSIYEILYARQ

>Tml_HRNR
MPKLLSSIVSVIEIYYQYATQDGEWDLLNKAELKELLENEFRHILKNPDDPDTVDVIMQSLDQDRNRKVDFTEYLMMIFKLAR
ACDKIIGKDYRQASGSKQRNHSYWHQEEQSETEEEQKGQESSSSQSSWSTGVENDSSSRGSRRIHYKTGLSSRRLGNQGGLSS
SEHKQSSGERRKSSSGYSKGRGKSKHGSYQPERSGSEEVGYTHSSNYRKRSNSANESDSCGQQRFGSGQPSQQRWHEPNTRSQ
SGNCEEQGYHPSSSEFSSYKKHRSSSGQPSSQRKHGSSSSGHSGSWRKEKHRSGSGNCSSFEQYGSSSGQSPNYGKHGSSTNH
SSSQRQDKSSSSSQLGHNRQQKQSGFESRESSSYKEYKSESGQSSTQRKQSNSGGCQSESCGRQKHKSGSVESPGYGQGGSDS
EQSSSYGHHGSGSGHSSSHGQYGTGSGQSSSHIHQEYGSRESSGYRQYGSDSSHSSRQKRHEYNSGGHSGICGRQKYRSGSSQ
SSNYGKYESGSNQSSSQHGFGSGQSSSYGQHGFGPGQSSSHSQYGSGSGQSSSFEQHKSGSGQSSSYGNHGSGSGQFSSHSQH
GSGSGQSSGCDQQGSSSGQSSSYGQLGSGSCQSSSQGQHGSGSGQCSSYSHGSGSGQYSNSEQYGSGSCPSSCSGQYGSGSGQ
HSGYEQCGQVESSSGCGFGQFTPYYGQPRSNTINELSICKEVYRQGGNSFLRGGNCRRGSTDSIFHSFCSSTPLYAYVKEQRH
YF

>Tml_RPTN
MAQLLNSILTVIKVFQKHAKENGDCASLCKKELKQLLLAEFGDILRRPNDPETVETILSILDRDRNGRVDFHEYLLLVFQLVQ
ACYRKLDIESYGDRTSRQEEGQEGAQDHKFPRNRDRQHRHRQEEERQDSHHGQSERQDRDSCHDQSEKQDRNSRHGQSEGQDR
DSHHGQSERQDQDFHHGQSERQDRDSSLNQSERQGQDSSYGQKLSHKSSNGQPKRQGYLFAVNQCEKPVQDSHHNQSERLGLR
SSCGQTRRLGQDTCSSRTEQQESGSIYGQSGRLAQESGCGQRDRQGLDSQYGQTDRQGQGYHYGQRETGETEIQGQNRYFQGS
EGIRRDTHVEQSGRSRRLNQQTQGQEVNPNQRQGSQKRLQAWERQLEDTQHHQHNLLAEIQQERSLCHKRRDWQSSGSEQDHR
QAQTQQSHSEGKSHRTEERQSHQSWGRQSHEGQESPCEAQDGQTREEEQSCQTRDKQTREDKQNHQRQDRQTHEDEQNRQRQI
KQIHEDEQNRQTQDRQTHENKQNCQRQDRQTHEDEQNHQRRDRQTDEEDQNLRRQQNTQTYDGEERYERSQNQQSHGTQQGCS
NRETFHMNEGGLSRGSQGRRSDPALHPTQGNGRPQRREQGGGHPTKAAISSNPLYDYVQEQRSHQY

>Tml_TCHH
MSALLRSIFDITEIFNQCASNDCNGAGLHKKDLKELLEREFGDPHDPETVALALELLDRDFNGLIDFHEYLLLIFRIAQACYY
ALGQATGLEEEKGAKYEGKGNPLNDRRQEDQRRFERRDRPVEEERRHKRQERARELVEEELQTDKQRRDEAQRLQRLEWLERE
EERREEIEQLPRRKGREPEDEQLQRRERQGQREPLRLEEELQRRREPLERQEEEEQLQRRERRELQRERQDEEEQLQRERQQE
AERRQRREGNELRLEEQLQRREQRERREQELAEQEEPELAESRTPRWQWQLESEADARQSKAYSRPRGQEEQRRRLEQEEEQR
RRQERERQLRREQDDAQRQAQPGQLRQEQRRDFQWQQEEERQRRRQRLSTRPSLKGQRKRELRADESQKRELLLREEEQRCQR
RQRAQEPQFREEEEQLQRQERDRQDLEEELLQREEREKRRLQERERQYREEQQLEQEEERLQREERERQYREEEQLEQEEERL
QREERERQYREEEQLEQEEERLQRERGRQERERQYREDEELQLEEEWLQREERKKRRRQEQMRQYREKQQLEQEEERPQRERR
RQERGRQYREEEELQRQDRKRQFRDEDQRRDLKRQLGKENEAGKNRVYSKSGKNEEKARQSEDSQVRERQFQQDRRSQQDELE
EERSFQEREEERRRRQQRDRQLQTADPLEGEEQEEAKRPDGMFREEEQLLREVREDRRRRQEGDRRFPEESFQQARDRRFQEE
KQLRRQERDRKFREEEQARRPLRDRQFRQEEQHLGQELEEEQLRRQERDRNLRGEQQLRRQQRDRKFHEDDQLRSQEREQLRQ
ERDRKSREVEEPRQQRKDELLRQKRDRQVREDEQLRRQERDRKFREEEELRLQQRDRQLREDEQLRRQEREQQLRQGRDRKSR
EDEQLRRQERDRQFREDQELRQERKDELLRQERDRKSREDEQLRRQERDRKLLEEEELRRERDEQQLRGQECEQQLRQERDRT
FREVDELRLEHEDKQLRRQERDRKLSEEEELRQEREEQQLRRQERDRQFRQVEELRLQQRDTQLREDEQLRRQERDRRFREEE
ELRLQQRDTQLREDEQLRLEREEQQLRRQEREQQLRQERDRRFREEEELRQEREEQQLRGQEREQQLRQERDRRFREVEELRL
ERAEQQLREERDRQLREEQQLRQERKEEKLRRQELDGAFSQDEQLNRAEQEEEQRRWRQRGSKFLEEEERLHQEREEEKRRRR
EQDRQFLQEEEQLRREEQEELRRRQERDRQYRAEEQFARDTRRQQEQEPRQEEEQRRRQERERKLRGEDKGKRQFVDPVVRSS
PLYEYIQEQRSQYRP

>Tml_TCHHL1
MPRLLRSVLCVIETFHKYAREDGNGVTLTCRELKQLLQGEFGDILQPHVIHAMEKNVNLLDIGSDGTISFDEFVLATCNLLNH
CYLDIQSLNSEPRQVSKPERKNPDDVDPQATIRNVQLTEETPPTQDKVVLPSGMAQSSQLNPEEKKRVEHNRVDPQEDFKTHN
LPREASEHNDSENQHLEGDEQIQEVAQDVQAAGDNGAQLEPNKAMTTSEETSSPTKGEGQDKEIPREAEKPAWEQKGTKTRAQ
LGQQGGNLGTQSSPAEETVQRPYEDHKVATEKSVQEHSKIQGHPCKQKTSPVQSMLTCQNKLLKVNHLRRRN

C

>Tml_PGLYRP3
MLPWLLVFSVLVLGARGLAVRDLSDGLLDLFSSISQLIQKGLNADAPTIVSRNEWGSRMLTCRAQLTQPVAYVITDELTGMEC
QEQNLCSWKLRGLQSHSVYTKGWCDVPYNFLVGDEGRVYEGVGWNIQGSHTQGYNNVSLGIAFFGSKMGSSPSPAALSAAGGL

ISYAIQKGYLSSRYIRPLILQSETCLVPQQPVRSRKACPNIVTRSAWGARETLCPKMNLPVKYVIIIHTAETSCNVSVDCQIR
VRDIQSFHINDRNFCDISYQFLVGQDGGVYEGVGWHTQGAHTYGYNDIALGIAFIGNFVERVPNNAALEAAQNLIQCAVDEGY
LVTDYLLVGHSDVVNTLSPGKALYNIIKTWPHFKH

>Tml_S100A9
MSQMEHSIETIINVFHQYSTRLGHPDTLNQKEFKQLVQKELANFLKVGLNSWQANEKKNDAAIKDIMEDLDTNVDKQLSFEEF
SILVGQLTEASHEKMHEGHPKGTDHSHGPGLGGKSGLGQGHGHSHSHDHDHGHKH

>Tml_S100A11
MANISSPTETERCIESLIAVFQRYAGQDGHGLTLSKKEFLNFMNTELGAFTKNQKDPGVLDRIMKKLDLNSDGQLDFPEFLNL
IGGLAQACHESFINRVR

Supplementary Figure S1. Amino acid sequences of proteins encoded by EDC genes of the manatee. **(A)** Amino acid sequences of proteins encoded by SEDC genes of the manatee. **(B)** Amino acid sequences of manatee SFTPs. **(C)** Amino acid sequences of proteins encoded by other EDC genes of the manatee. The following amino acid residues are highlighted to show the peculiar amino acid compositions of SEDCs and SFTPs: lysine (K) and glutamine (Q) as potential sites of transglutamination; cysteine residues (C) as potential sites of disulfide bonds; proline (P). When available, the GenBank accession number is shown behind the protein name. Only the S100A proteins encoded by genes that flank *PGLYRP3* and *TCHHL1* are included here. SEDC, simple (single coding exon) epidermal differentiation complex gene; SPRR, small proline rich protein; SFTP, S100 fused-type protein; Tml, *Trichechus manatus latirostris*.

A

>Dd_LOR
MSHQKKQPTPIPPVGCVKIGGGGGGGGGGGCGGGSGGCEGGSGGGIKYYGGGGSGCGSSGGGGGDSSCGGGIKYCGDGGSSGG
GCGGGIKYCGDGGSSGGGCGGGIKYYGGGGSGCGSSGGGGGDSSCGGGIKYCGDGGSSGGGCGGGIKYCGDGGSSGGGCGGGI
KYYGGGGSGCGSSGGGGGDSSCGGGIKYCGDGGSSGGGCGGGIKYCGDGGSSGGGCGGGIKYCGDGGSSGGGSGGGIKYCGDG
GSSGGGSGGGIKYCGDGGSSGGGSGGGIKYCGDGGSSGGGSSCWGGSSGGFGGSHSGQQVQYQSYGGGCSGGSSGGGSGCFSS
GGGGGGGSCYGGGSSGGRGDSSCGGGSAGGGSGSGKGVPVCHQTQQKQAPTWPCK

>Dd_PRR9
MSFNEQQCKQPCVPPPCLQKTQEQCQTKAEEVCLPTSQDPCQEKCLAQVQEVGLPQCQELSQENCPQQGLDPCLPPCQDQCLP
QCVEPCQELSQTKCVEIFPQKSQEKCLPPGKGK

>Dd_LELP1
MSSDDKSKSGEQKTEPKCEQKCEPKCEQKCESKCQPSCLKKLLQRCSDKCPRDKCPSPCPPKCPPPCPPPCPPPCPPPKPCTK
PCPPKCPSLCPPPE

>Dd_SPRR2EL1
MSYQQQQCKQPCQPPPVVCPPKCPEPCPPKCHEPCPPQKCPELCPPPKCPEPCPPVQCPPEPCQQKCPPVQVYQPCQQKCPPK
SK

>Dd_SPRR2EL2
MSYQQQQCKQPCQPPPVVCPPKCPEPCPPKCPEPCRPKCLEPCPPPKCPEPCPPVQCPPEPCQQKCPLVQVYQPCQQKCPPKS
K

>Dd_SPRR2EL3
MSYQQHQCKQPCQPPPVVCPPKCPEPCPLLKCPEPCPPPKCPEPCPPPKCPEPCPPVQCPPEPCQQKCPPVHVYQPCQQKCPP
KSK

>Dd_SPRR2EL4
MSYQQQQCKQPCQPPPVVCPPKCPEPCQPKCPEPCPPQKCPEPCPPPKCPEPCLPVQCPPEPCQQKCPPVQVYQPCQQKCPPK
SK

>Dd_SPRR2EL5
MSYQQQQCKQPCQPPPVVCPPKCPEPSPPPKCPEPCPPKCLEPCPPKCPEPCPPPKCHEPCPPVQCPPEPCQQKCPPVQVHQP
CQQKCPPKSK

>Dd_SPRR2EL6
MSYQQQQCKQPCQPPPVVCPSKCPEPCPPKCPEPCPPQKCPEPCPPPKCPEPCLPVQCPPEPCQQKCPPVQVYQPCQQKCPPK
SK

>Dd_SPRR2EL7
MSYQQQQCKQPCQPPPVVCPPKCPEPCPPKCPEPCPPPKCPEPCRPLQCPPQPCQQKCPPMQVYQPCQQKCPPTSK

>Dd_SPRR2EL8
MSYQQQQCKQPCQPPPVVCPPKCREPCPLPKCPEPCPPPKCPEPCPPAKCPEPCPPVQCPPEPCQQKCPPVHVYQPCQQKCPP
KSK

>Dd_SPRR2EL9
MSYQQQQCKQPCQPPPVVCPPKCPEPSPPPKCPEPCPPKCLEPCPPKCLEPCPPKCPEPCPPPKCPEPCPPVQCPPEPCQQKC
PPVQVHQPCQLKCPPKSK

>Dd_SPRR2EL10
MSYQQQQCKQPCQPPPVVCPPKCPEPCQPKCPEPCPPQKCPEPCPPPTCPEPCLPVQCPPEPCQQKCPPVQVYQPCQQKCPPK
SK

>Dd_SPRR2EL11
MSYQQQQCKQPCQPPPVVCPPKCREPCPLPKCPEPCPPPKCPEPCPPAKCPEPCPPVQCPPEPCQQKCPPVHVYQPCQQKCPP
KSK

>Dd_SPRR2EL12
MSYQQQQCKQPCQPPPVVCPPKCPEPCPLKCPEPCPPPKCPEPCRPLQCPPQPCQQKCPPMQVYQPCQQKCPPTSK

>Dd_SPRR2EL13
MSYQQQQCKQPCQPPPVVCPPKCPEPCPPQKCPEPCPPPKCAEPCPPVQCPPEPCQQKCPPVHVYQPCQQKCPPKSK

>Dd_SPRR2EL14

MSYQQQQCKQPCQPPPVVCPPKCPEPCPPKCPEPCPPPKCPEPCPPPKCPEPCPPVQCPPEPCQQKCPPVQVQQPCQQKCPPK
SK

>Dd_SPRR2EL15
MSYQQQQCKQPCQPPPVVCPPKCPEPCPPKCPEPCPPPKCPEPCPPPKCPEPCPPVQCPPEPCQQKCPPVQVQQPCQQKCPPK
SK

>Dd_SPRR2EL16
MSYQQQQCKQPCQPPPVVCPPKCPEPCPPKCPEPCPPPKCPEPCPPPKCPEPCPPVQCPPEPCQQKCPPVQVQQPCQQKCPPK
SK

>Dd_SPRR2EL17
MSYQQQQCKQPCQPPPVVCPPKCPEPCPPKCPEPCPLPKCPEPCQPKCPEPCPPKCPEPCPPVQCPPEPCQQKCPPVHVYQPC
QQKCPPKSK

>Dd_SPRR1B
MSSQQQKKPCTLPPQLQQQQVKQPCQPPPQEPCVPQTKEPCHPKVPEPCHPTVPEPCHPTVPEPCHPTVPEPCHPKVPEPCPS
TVTPAPAPQKTKQK

>Dd_SPRR1AL1
MSSYQQKQSFTPPPHPHEQQVIQPCLPPQEPPVPITETPGHPQVPPPGNTKVPEPGNPKVPDSV

>Dd_SPRR1AL2
MSSYQQKQPFTPPPQPHEQQVIQPCLLPQEPPVPITETPGHPQVPPPGNTKVPEPGYPKVPDSVCCKDPEPCPSPVSPDPDQQ
KTKQN

>Dd_SPRR1B2
MSSQQQKKPCVPPPQLQQQQVKQPCQPPPQEPCVPKTKEPCHPKVPEPCHPTVPEPCHPKVPEPCHPTVPEPCHPKVPEPCPS
TVTPAPAQQKTKQK

>Dd_SPRR4
MIFYKLVDRERWWEPFSWYSWFSSRFTCSCSNVFPAARAAAVPTPETQQQQVKQPCQPPPVKCQETCAPQTKDPCASQATKQC
SPKGTATAVQQKCPPAQQAPKSKQK

>Dd_IVL
MSQQYTPPVTIPADICQPPIKTVSPPVDTQEEQVKQPAPQPSPGQKVPSEPPVKVPSEQGEKHTAPVKEVPEQELWQQQQPQG
QEEHQEQQHQEPQELEQQQDQQQQMPQELKQHQEQQQQQPQELEQHQDQQQQEPQKLEQHQDQQNQEPQELEQHQDQQQQEPQ
KLEQHQDQQNQEPQKLEQHQDQQNQEPQKLEQHEEQQQQQPQEPQEPQEQVQHQEQQQPPQEPQKQEHQHQEQHQEHQESESL
EQQPKQEKAQKEEQLKEQLDQEKKLLDQQMDQELEKKDKQLGKKEEQLPEHLEHQEQQSL

>Dd_SMCP
MCDQPKRNPCCQPPRQCCTSPCQCCPSPRQCCLPPRKCCPLPRQCCPPPRQCCPSAPQCCIKQQCLEAKAECTCLNEESEPKP
PQTQDRGSHSPQNAPRPGILGQQKKPSK

>Dd_LCE7A
MSYQQSKQKCKLPVKCLPKCPPKCPPQALQAPAPCLPPAPSCCAPSYCISGFGGKCCLVSHQFPLFYLHQPQPSCENESLLYS
SCCHSSEDRC

>Dd_LCE6A
MSQKKQQSCGPPDTPKCSPLQCPNPYLAPSCAPCSGSCCLGSQRPRAQSPVCPKRAHWKPRCLLGGTIYHCKEEEC

>Dd_LCE1
MSCQQNQQQCQPSPKCTPKCPPKCPTTKCPPKYPPVSSCCSVSSGGCCGPSSEGCCGSSSGGGGCCLSHHRRHRSYRHRHQSS
DCWSQP

>Dd_KPRP
MCDQQLIPSCLPLPQCCVKGSSFFPSPSPCATGQVVVQAPCEMQIVECPAPCPVQVSQVKCVAPCQSKTTQVKGQASCQTQTT
QVESQAVCQPEVSYVQCEVPCPVQTCYVEHAPVCCRETCYVEYPVQTCVTCPVPQPVQTYVTYSPVCQNQGRFSVQGQYQGSY
SRCTPQSQSRASFSTYAPQCQTQGSYGSFTALRRSQSARRCLPFSQLEPCYRSCSAPRRLGCYYSICLPSRCCSDSCYCTPPR
RSEPIYNSGCSRGRTSGRSQRCGPKCRVEISSPCYPKQVPPQKCSVQIPPIGRCSKICAPPFSCGASCPDLRPCQLRSSSFRP
PRRLDQCPEPLLPPCPLPEPRPCPRPCPLPEPRPCPRPCPLPEPRPYPRPCPLPEPRPCPRPCPLPEPRPCPRPCPLPEPRPC
PRPCPLPEPRPCPPPCPLPEPRPCPRPCPCPCPERCLSPEPCPCPPPCWSSEPCLYPEPRPAPSPAPRLALRSRPVRYEFPHR
HLHAQPRDLREPRPLPEPIPLPAPCPSPEPCVELQSYPNPCSGPKPISCPPDLGCQESSPFRLDSEVPSCGPGGYNQGRE

>Dd_KPLCE
MCDQQKQQQFPQSCVKGSGLGFVQSIKGKFVKCAAPCETKTVSVTCPDPCQMQTLVKCPAPCPTQTYVQYQVPCQTQTCVKCP
VPYQTTCVKCPTPCQTTYVKCPTPCQTTYVKCPTPCQTTCVKCPTPCQTTYVKCPTPCQTTCVKCPTPCQTTCVKCPTPCQTT
YVKCPTPCQTTYVKCPTPCKTQTYYVQCPSPCHTYYAQLPASSTVQSLGSKDCVPDLYSVPCSTSYSCLAPRTFRVSPLRCWV
QHPQGCCEYSGCCISGCCSCGSGCCCLGIIPMRSCGPACCDHEDDCCC

>Dd_LCE2AL1
MSWQQNQQQCQLPPKCTPKCPPKCPIPKCPPKCPPVSPCCSVSCGDCCGPNSGCCSSGGGSCCLSHHRHHIFHRRRWHQSPDC
SECDPCGHPGCCSGSGGCC

>Dd_LCE2AL2
MSWQQNQQQCQLPAKCTPKCPPKCPIPKCPPKCPPKCLPVSPCCSVSCGDCYGSSSGCCSCGGGGCCLSHHRHHIFHRRRWHQ
SPDCCECDSCGHSGCCMGSGGCC

>Dd_LCE2AL3
MSWQQNQQQCQLPANCTPKCPPRCPIPTCPPKCPQKCPPVSPCCSVSCGDCCGPSSGCCRSGGGSCCLSHHRHHLFHRRRWHQ
SPDCCNCDPCGHSGCCSGSGGCC

>Dd_LCE2AL5
MSWQQNQQQCQLPAKCTPKCPPKCPIPKFPPKCPPKCPAVSPCCSVSCGDCCGPSSGCCSSGGGRCCLSHHKHHVFHRRRWHQ
SPDCCECDPCGHSGCCSGSRACC

>Dd_LCE2AL6
MSWQQNQQQCQFPAKCTPKYPPKCPIPKCPPKCPPKCLPVSPCCSVSCGDCCGPSSGCCSSGAGSCCLSHHRYHIFHRRRWHQ
SPDCCECDPCGHSGCCSGSGGCC

>Dd_LCE2AL7
MSWQQNQQQCQLPAKCTPKCPPKCPIPMCPPKCPPKCPPVSPCCSVSCGDCCGPSSGCCSSGGSSCCLSHHRHHIFHRRRRHQ
SPDCCECDPCGHSGCCSGPGGCG

>Dd_LCE2AL8
MSWQQNQQQCQLPAKCTPKCPPKCPIPTCPPKCPPKCPPVSPCCNVSCGDCCGPSSGCCSSGGSSCCLSHHRHHIFHRHRRHQ
SPDCCECDPCGHSGCCSGSGGCC

>Dd_LCE2AL9
MSWQNQQQCQLPAKCTPKCPPKCPIPQCPPKCPPNCPPVSPCCSVSCRDCCGPSSGFCSSGGGGCCLSHHRHHLFHRRRWHQS
PDCCECDPCGHSGCCSGSGGCC

>Dd_LCE2AL10
MSWQQNQQQCQLPAKCTPKCPPKCPIPKFPPKCPPKCPPVSPCCSVSCEDCCGPSSGCCSSGGGSCCLSHHQHHVFHRRRWHQ
SPDCCGHSGCCSGSRGCC

>Dd_LCE2AL11
MSWQQNQQQCQLPAKCTPKCPPKCPIPTCLPMCPPKCPPVAPCCSVSCGDCCGPSSGCCSSGGGGCCLSHHRHHLFHRRRWHQ
NPDCCECDPCGHSGCCLGSGGCC

>Dd_LCE2AL12
MSWQQNQQQCQLPAKCNPKCPPKCPIPTCPPKCPPKCPPVSPCYSVSCGNGCGSSSGCCSSGGGGCSLSHHRHHIFHQRRWHQ
SPDCCESDPCGHSGCSSGSGGCC

>Dd_LCE2AL13
MSWQQNQQQCQLPAKCTPRCPPKCPIPMCPPKCPPKCPPVSPCCSVSCGDCCGPSSGCCSSGGSSCCLSHHRHHIFHRRRRHQ
SPDCCECDPCGHSGCCSGSGGCC

>Dd_LCE2AL14
MSWQQNQQQCQLPAKCTPKCPPKCPIPKCPPKCPPKCPPVSPCCSVSCGDCCGPSSGCCSSGGGGCCLSHHRHHLFHRRRWHQ
SPDCCECDPCGHSGCCSGSGGCC

>Dd_LCE2AL15
MSWQQNQQQCQLPAKCTPKCPPKCHILKCPPKCPPKCPPVSPCCSVSCGDCCGPSSGCCSSGGGGCCLSHHRHHLFHRRRWHQ
SPDCCECDPCGHSGCCSGSGGCC

>Dd_LCE2AL16
MSWQQNQQQCQLPAKCTPKCPPKCPIPTCPPKCPPKCPPVSPCCNVSCGDCCGPSSGCCSSGGSSCCLSHHRHHIFHRRRRHQ
SPDCCECDPCGHSGCCSGSGGCC

>Dd_LCE2AL17
MSWQNQQQCQLPAKCTPKCPPKCPIPQCPPKCPPNCPPVSPCCSVSCGDCCGPSSGCCSSGGGGCCLSHNRHHLFHRRRWRQS
PDCCECDPCGHSGCCSGSGGCC

>Dd_LCE2AL18
MSWQQNQQQCQLPAKCTPKCPPKCPIPNCPPKCPPKCPPVSPCCSVSCGDCCGTSSGCCSSGGGGCCLSHHRHHLFHRRRWHK
SPDCCECDPCGHSGCCSGPGGCC

>Dd_LCE2AL19
MSWQQNQQQCQLPAKCTPKCPPKCPIPTCLPKCPPKCPPVSPCCSVSCGDCCGPSSGCCSSGGGGCCLSHHRHHLFHRRRWHQ
SPDCCECDPCGHSGCCSGPGGCC

>Dd_LCE2AL20
MSWHQNKQQCQLPAKCTPKCPPKCPIPKCPPKCPPKCPPVPPCCNVSCGDCCGPSSGCCSSGGGGCCLSHHRHHLFHRRRWYQ
SPHCCECDPCGHSGCCSGSGGCC

>Dd_LCE2AL21
MSWQQNQQQCQLPYKCTPKCPPKCPIPTCPPKCPPKCPPVSPCCSVTCGDCCGPSSGCCSSGGGGCCLSHHRHHIFHRHRRHQ
SPDCCECDPCGHSCCCSGSGGCC

>Dd_LCE2AL22
MSWHQNKQQCQLPAKCTPKYPPKCPIPKCPPKCPPKCPPVPPCCNVSCGDCCGPSSGCCSSGGGGCCLSHHRHHLFHRRRWHQ
GPDCCECDPCGHSGCCSGSGGCC

>Dd_LCE2AL23
MSWQQNQQQCQLPAKCTPKFPPKCPIPTCPPKCPPKCPPVSPCCSVSCGDCCGPSSGCCSSGGSSCCLSHHRHHIFHRRRRHQ
SPDCCECDPCGHSGCCSGSGGCC

>Dd_LCE2AL24
MSWQQNQQQCQLPAKCNPKCPPKCPIPTCPPKCPPKCPPVSPCCSVSCGDCCGSSSGCCSSGGGGCSLSHHRHHIFHRRRWHQ
SPDCCECDPCGHSGCSSGSGGCC

>Dd_LCE2AL25
MSWQNQQQCQLPAKCTPKCPPKCAIPTCPPKCPPKCPPVSPCCSVSCGDCCGPSSGCCSSGGGGCCLSHHRHHLFHRRRWHQS
PDCCECDPCGHSGCCSGSGGCC

>Dd_LCE2AL26
MSWQQNQQQCQLPAKCTPKCPPKCPIPKFPPKCPPKCPAVSPCCSVSCGDCCGPSSGCCSSGGGSCCLSHHQHHVFHRRRWHQ
SPDCCECDPCGHSGCCSGSRGCC

>Dd_LCE2AL27
MSWQQNQQQCQFPAKCTPKYPPKCPIPTCLPKCPPKCPPVSPCCSVSCGDCCGPSSGCCSSGGGSCCLSHHRHHIFHRRRWHQ
SPDCCECDSCGHSGCCSSSVGCC

>Dd_LCE2AL28
MSWHQNQQQCQLPAKCTPKCTPKCPIPKCPPKCPPKCPPKCPPVPPCCNVSCGDCCGPRSGCCSSGGGGCCLSHHRHHLFHRR
CWHQSPDCCECDPCGHSCCSGSGGCC

>Dd_LCE2AL29
MSWQQNQQQCQLPAKCTPKCPLKCPIPKCPPKCPPVSPCCSVSCRDCCGPSSGCCSSGGGSCCLSHHRHHIFHRRRWHQSPDC
CECDPCGHSGCCSGSGGCC

>Dd_LCE2AL30
MSWQQNQQQCQLPSKCTSKCPPKCPIPTCPPKCPPKCPPVSPCCSVSCGDCCGPSSGCCSSGGGGCCLSHHRHHIFHRRHRHQ
SPDCCECDPCGHYGCCSGSGGCC

>Dd_LCE2AL31
MSWQQNQQQCQLPAKCTPKCPPKCPIPNCPPKCPPKCPPVSPCCSVSCGDCCGPSSGCCSSGGGGCCLSHHRHHLFHRRRWHQ
SPDCCECDPCGHSGCCSGPGGCC

>Dd_LCE2AL32
MSWQQNQQQCQLPYKCTSKCPPKCPIPTCPPKCPPKCPPVSPCCSVTCGDCCGPSSGCCSSGGGGCCLSHHRHHIFHRRRRHQ
SPDCCECDPCGHSGCCSGSGGCC

>Dd_LCE2AL33
MSWQQNQQQCQLPYKCTPKCPPKCPIPTCPPKCPPKCPPVSPCCSVTCGDCCGPSSGCCSSGGGGCCLSHHRHHIFHRHRRHQ
SPDCCECDPCGHSCCCSGSGGCC

>Dd_LCE2AL34
MSWHQNKQQCQLPAKCTPKYPPKCPIPKCPPKCPPKCPPVPPCCNVSCGDCCGPSSGCCSSGGGGCCLSHHRHHLFHRRRWHQ
GPDCCECDPCGHSGCCSGSGGCC

>Dd_LCE2AL35
MSWQQNQQQCQLPAKCTPKCPPKCPIPTCPPKCPPKCPPVSPCCNVSCGDCCGPSSGCCSSGGSSCCLSHHRHHIFHRRRRHQ
SPDCCECDPCGHSGCCSGSGGCC

>Dd_LCE2AL36
MSWQQNKQQCQLPAKCTPKCPPKCPIPMCPPKCPPMCPPVPPCCNVSCGDCCGPSSGCCSSGGGGCCLGHHRHHLFHRRRWHQ
SPDCCECDPCGHSGCCSGSGGCC

>Dd_LCE2AL37
MSWQHNQQQCQLPAKCTPKCPPKCPIPMCPPKCPPKCPPVSPCCSVSCGDCCGPSSGCCSSGGSSCCLSHHRHHIFHRRRRHQ
SPDCCECDPCGHSGCCSGSGGCC

>Dd_LCE2AL38
MSWQQNQQQCQLPAKCTPKCPPKCPIPKCPPKCPPKCPPVSPCCSLSCGDCCGPSSGCCSSGGGGCCLSHHRHHLFHRRRWHQ
SPDCCECDPCGHSGCCSGSGGCC

>Dd_LCE2AL39
MSWQQNQQQCQLPAKCTPKCPPKCHILKCPPKCPPKCPPVSPCCSVSCGDCCGPSSGCCSSGGGGCCLSHHRHHLFHRRRWHQ
SPDCCECDPCGHSGCCSGSGGCC

>Dd_LCE2AL40
MSWQNQQQCQLPAKCTPKCPPKCPIPQCPPKCPPNCPPVSPCCSVSCGDCCGPSSGCCSSGGGGCCLSHHRHHLFHRRRWRQS
PDCCECDPCGHSGCCSGSGGCC

>Dd_LCE2AL41
MSWQQNQQQCQLPAKCTPKCPPKCPIPSCPPKCPPKCPPVSPCCSVSCGDCCGTSSGCCSSGGGGCCLSHHRHHLFHRRRWHK
SPDCCECDPCGHSGCCSGPGGCC

>Dd_LCE2AL42
MSWQQNQQQCQLPAKCTPKCPPKCPIPTCLPKCPPKCPPVSPCCSVSCGDCCGPSSGCCSSGGGGCCLSHHRHHLFHRRRWHQ
SPDCCECDPCGHSGCCSGPGGCC

>Dd_LCE2AL43
MSWHQNKQQCQLPAKCTPKYPPKCPIPKCPPKCPPKCPPVPPCCNVSCGDCCGPSSGCCSSGGGGCCLSHHRHHLFHRRRWHQ
GPDCCECDPCGHSGCCSGSGGCC

>Dd_LCE2AL44
MSWQQNQQQCQLPAKCTPKCPPKCPIPTCPPKCPPKCPPVSPCCNVSCGDCCGPSSGCCSSGGSSCCLSHHRHHIFHRRRRHQ
SPDCCECDPCGHSGCCSGSGGCC

>Dd_LCE2AL45
MSWQNHQQCQLPAKCTPKCPPKCPIPQCPPKCPPKCPPVSPCCSLSCGDCCGPSSGCCSSGGGGCCLSHHRHHLFHRRRWHQS
PDCCECDPCGHSGCCSGSGGCC

>Dd_LCE2AL46
MSWQQNQQQCQLPAKCTPKCPPKCPIPTCPPKCPPKCPPVSPCCSVSCGDCCGPSSGCCSSGGGGCCLSHHKHHIFHRRRWHQ
SPDCCECDPCGHSGCCSGSGGCC

>Dd_LCE3DL
MSCQQNQQQCQPPPKCPPKIPAQCWPPVSSGCVPSSGACHGPSSEGGCCLSPHRRRRSHQCQHQSSNSSDAGCSQQSGGSHCS
HSSGGLLLTWAVWTRPAIVKETKTQSPKDRPSLTQPFPDPLSQAELHGCACGGSELSLEGFSCLMSRISQFPPHPSPADACQG
LCLTLWNIKLLLITTQVSLVCLLCPSPRFLSPAQPNTGE

>Dd_LCE3DL2
MSCQQNQQQCQPPPKCPSPKCPPKSPAQCWPPVSSGCAPSSGGCHGPGSEGGCCLSPHRCRRSHRCRCQSSDCSDNGRGQQSG
GSHCGHSSGGCC

>Dd_CRCT1
MSSQQTSKGFSKGTSKGPAPCPAPTPAPASPSSCCGHGCCGSSSCCGDSRCCNSGCCGSSSTSCCCFPRRRRHRRCCCCCGGS
QRSQC

B

>Dd_CRNN
MPQLLRNIHGIIEAFGRYAKTEGDCTVLTRGELKRLLEHEFADVIVKPHDPVTVDEVLRLLDEDNTGTVEFKEFLVLVFKVAQ
ACFKTLSESPEGACGSQESRSHHPGASQELREGQRSHTEVGWAGRGQLHEGSSRGQSKHAFLEHDGSSAQVSLHDKQVESQRQ
ESPSQQAQETGHTEHTQTAQDQRHQTRVGWSERQFQTREQGRAHQTSETVTGTLTQTQAGTTQTLGQDRSHQMRSTSTQSQES
TCGQTRGTHSQDRSHQMGSTSTQSQESTCGQMGGTHSQDRSHQTGSISTQSQESTYGQTRGTHGQDKSQTSQVVIGGHVQTQV
GSQTQTVEQDRSRQMGSTSTQSQESTCGQTRGTHSQDKSRQTGSTSTQSQESTYGQTGGTHSQDKSRQTGSTSTQSQESTYGQ
TGGTHSQDRSRQTGSTSTQSQESTCGQTRGTHSQDKSRQTGSTSTQSQESTYGQTGGTHSQDKSRQTGSTSTQSQESTYGQTG
GTHSQDRSRQTGSTSTQSQESTCGQTRGTHSQDRSHQMGSTSTQSQESTCGQTRGTHSQDRSHQMGSTSTQSQESTCGQMGGT
HSQDRSHQTGSISTQSQESTYGQTRGTHGQDKSQTSQVVIGGHVQTQVGSQTQTVEQDRSRQMGSTSTQSQESTCGQTRGTHS

QDKSRQTGSTSTQSQESTYGQTGGTHSQDRSHQTGSTSTQSQESTYGQTRGTHSQDRSRQTGSTSTQSQESTYGQTGGTHSQD
RSHQTGSTSTQSQESTYGQTGGTHSQDRSRQTGSTSTQSQESTYGQTGGTHSQDRSRQTGSTSTQSQESTYGQTGGTHSQDRS
RQTGSTSTQSQESTYGQTGGTHSQDRSRQTGSTSTQSQESTYGQTGGTHSQDRSCQTGSTSTQSQESTYGQTRGTETNSQDRN
QMSQVVTGHIQTQAGSQTQVHPQAVEQDRSQTGRHAGAREEGQSQTQSGSGQTWTRVSNYEAGETVLEGQPQPGAGTLPGSQD
GSSMHPSYSVTGGQGESNVVVEEWVDDHTREMVIQRQDQGTLPSSASSALGGETAQPKERQGITAKGLYSYFQSTKP

>Dd_FLG2
MTDLLRSVVTIIDIFYKYTKQDGECATLSKDELKELLEKEFHPILKNPDDADTVDVIMHMLHQDHDR

>Dd_FLG
MSILLEKIVAIIDLFQQYSSKDKENDTLSKKELTELLEREFRSILKHPDDPDTTDVFMHVLDVDHNEKINFTEFLLMVFKLAQ
AYYESMKRKNFKATSTKQKTHGQECQNEDYEIEEEEKEEEQRRESILSSRHGRKGKRSPIRTDKKKHGSISSSKGRRDSSSPF
GHREKNERKHHHHTGKYYNRSSSTDLGEERDTSSIGQKREQEGDEHDYGYEKRGRVSDTHEDTSQEESAHSGRQSGSSSRNHH
GSTHGQARDSFRHSRSLEQQTADHVQSHSGHVHSDSTATGRQGSVHGQSVHSARHSGSHQGQRSTGVQSRSTTRGRQEFHQDQ
SADRSRVSQSGPGQSSSEFRTDTHRESSVSQASDSEGQSEDSERQSGSSSRNHYGSTHGQARDSSRHSRSHQDQRANRAWSGS
AMDTQILLPQEEKDAFMERL

>Dd_HRNR
MPKLLSSIVSVIEIYYQYATQDGEWDLLNKAELKELLENEFHHILKNPDDPDTVDVIMQSLDQDRNRKVDFTEYLMMIFKLAR
ACDKIIGKDYRLASGSEQRNHSRWHQEEQSETEEEQKGQESSSSQSSWSTGAENDSSSRGSRCIQYETGFSSRRLGNQGGLSS
SEHRESSGERRKSSLGYSKGRGKSKHASYQPGRSGSEEVGYTHSSNYRKRSNSANESDSCGQQRFGSGPPSQQRWHEPNAGSQ
SGNCEEQGYHPSSSEFSSYKKHRSSSGQPSSQRKHGSSSSGHSGSWRKERHRSGSGNCSSFEQYGSSSGQSPNYGKHGSSTNH
SSSQKQDKSSSSGQLGHNRQQKQSGFESRESSSYKEYKSESGQSSTQRKQSNSFGCQSQSCGRQKHESGSVESPGYGQGGSDS
KQPSSYDHHGSGSDHSSSHGQYGTGSGQSSSHIHQEYGSRESSGYRQYGSGSSHSSRQKRHEYNSGGHSGICGRQKYGSGSSQ
SSNYGKYESGSNQSSSQHGFGSGQSSSYGQHGFGPGQSSSHSQYGSGSGQSSSYEQHKSGSGQSTSYGNHGSGSGQSSSHSQH
GSGSGQSSGCDQQGSSSGQSSSYGQLGSGSCQSSSQGQHGSGSGQCSSYSHGSGSRQYSNSEQYGSGSYPSSCSGQYGSGSGQ
HSGYEQCGQVESSSGCGSGQFTPSYGQPRSNTINKLSICKEVYRQGGNSFLRGGNCRRGSTDTISHSFCSSTPLYAYVKEQRH
YF

>Dd_RPTN
MAQLLNSILIVIKVFQEHAKENGDCASLCKKELKQLLLAEFGDILRRPNDPETVETILSILDRDRNGRVDFHEYLLLVFQLVQ
ACYRELDIESCGDRTSQQEEGQEGAQDHKFPRNRGRQHRHRQEEERQDSHHGQSERQDRDSCHDQSERQDRNSRHGQSERQDQ
DFCHGQSEGQDRDSSLNQSERQVQDSSYGQKLSRKSSNGQPKRQGYLFAVNQCEKPFQDSHHNQSEKLGQRSSCGQCRRLGQD
SCSSHTEQQESGSICGQSGRLAQESGCGQRDRQGLDSHYGQTDRQGQSSHYEQIDRQGQSSLHGQTDKQGLSSHYGQTDRQGQ
SSHYGQTDRQGQGYHYGQRETRETEIQGQNRYFQGSEGTRRDTHVEQSGRSGRLNQQTQGQEVNPNQRQGSQNRLQAWERQLE
DTQHHQHNLLAQIQQERSLCHKRRDWQSSGSEQGHRQAQTQQSHSEGKSHRTEDRQSHQSWGRQSHEGQKSPCEAQDGQTHEE
EQSCQTRDKQTREDEQNHQRQDKQTHEDEQNRQRQIKQTREDKQNRQRQDRQTHENKQNCQRQDRQTHEDEQKRQRQIRQTRE
VEQNQQRQDKQTHEDKKTRQKQDRQIHENEQNHQKQDRQTHEDEQNRQRQDRQTHENKQNCQKQDRQTHEDEQNHQRRERQTH
EEDQNLRRQQNTQTYDREERYERSQNQQSHGTQQGCSNRETFHMNEGGLSRGSQGRRSDPALHPTQGNGKPQRREQGGGHPTK
AAISSNPLYDYVQEQRSHQY

>Dd_TCHH
MSALLRRIFDITEIFNQCASNDCNGAGLHKKDLKELLEREFGDVLRRPHDPETVALALELLDRDWNGLIDFHEYLLLIFRIAQ
ACYYSLGQATGLEEQKGAKYEGKGNPMNDRRQEDQRRFERRDRPVEEERRRKRQEQARELVEEELQRDKQRRDKAQSLQRLEW
LEREEERREEIEQLQRRKGREPEDEQLQRRERRERQEQREPLRREEELQRRREPLERQEEEEQLQRRERRELQRERQDEEEQL
QRERQQEAERRQRREGNELRLEEQLQRREQERREQELAEQKEPELAESGTPRWQWQLESEADARQSKAYSRPRGQEEQRRRL
EQEEEQRPRQERERQLRREQDDAQRQARPAQLREEQRRDFQWQLEEERERPRQRLSARPSLKGQREREWRADERQKRELLLRE
AELRCQRRQRAQEPQFREEEEQLQRQERDRQDLEEELLQREEREKRRRQEQVRQYRDEEQLEQEEERLQREEREKRRHQEQVR
QYRDEEQLEQEEERLQREEREKRRRQEQVRQYRDEEQLEQEEERLQREEREKRRRQEQVRQYRDEEQLEQEEERLQREERERQ
YREEQQLEQEEERLQREERERQYREEQQLEQEEERMQRERRRQERGRQYREEEELQRQDRKRQFRDEDQRGNLKRQLGKENEA
GKNRVYSKSGKNEEKARQSEDSQVRERQFQQDRRPEQDELEEERSLQEREQERRRRQQRDRQLQTADPLEGEEQEEAKRPDGM
FREEEQLLREVREDRRRRQEGDRRFPEESFQQARDRRFQEEEQLRRQERERKFREEEQVHRPLRDRQFREEEQQLRRQQRDRK
FHEDDQLRSQERDRKSREVEELRQERKDDLLRQERDRQVREEEELRLQQRDRQLREDEQLRRQERDRKLREEEELRQERDEQQ
LRRQERDRQFREVEELRRQEREQQLRQERDRQFREDEQLRRQERDRQFREEEELRRQQRDRQLREEEELRRQQRDRQLREDKQ
LRLEREEQQLRRQEREQQLRQERDRQFREDKELRQEREEQQLRGQERDRQFREVEELRLEREEQQLREERDRQLREEQQLRQE
RKEEKLRCRELDGAFSQDEQLNRAEQEEEQRRWRQRGSKFLEEEERLLQEREEEKRRRREQDRQFLQEEEPLRREEQEELRRR
QERDRQYPAEEQFARDTRRQQEQELRQEEAQRRRQERERKLRGEDKGKRQFVDPVVRSSPLYEYIQEQRSQYRP

>Dd_TCHHL1
MPRLLRSVLCVIETFHKYAREDGNGVTLTCRGLKQLPQGESGGILQPHVIHALEKNVNLLDIGSDGTISFDEFVLATCNLLNH
CYLDIQSLNSEPRQVSKPEKKNPDDMDPQATSRNVQLTEETPPTQDKAVLPSGMAQSSQLNPEEKKRVEHNRVDPQEDFKTHN
LPREASEHNDSKNQHLEGDEQIQEVAQDVQAAGDNGAQLETNKAMTTSEETSSPTKG

C

>Dd_PGLYRP3
MLPWLLVFSVLVLGARGLAVRDLSDGLLGLFSSISQLIQKGLNADAPSIVSRNEWGSRMLTCRAQLTHPVAYVITDELTGMEC
QEQNLCSWKLRGLQSHSVYTKGWCDVPYNFLVGDEGRVYEGVGWNIQGSHTQGYNNVSLGIAFFGSKMGSSPSPAALSAVGGL

FSYAIQKGYLSSRYIRPLILQSETCLVPQQPVRSRKACPNIVTRSAWGSRETLCPKMNLPVKYVIIIHTAGTSCNVSVDCQIR
VRDIQSFHINDKNFCDISYQFLVGQDGGVYEGVGWHTQGAHTYGYNDIALGIAFIGNFVERVPNNAALEAAQNLIQCAVDEGY
LVTDYLLVGHSDVVNTLSPGKALYNIIKTWPHFKH

>Dd_S100A9
MSQMERSIETIINVFHQYSTRLGHPDTLNQKEFKQLVQKELANFLKNEKKNDAAIKDIMEDLDTNEDKQLSFEEFSVLVGQLT
EASHEKMHMCHPKGTDHSHGPGLGEESDLGQGQGQCHSHGHDHGHKH

>Dd_S100A11
MAKISSPTETERCIESLIAVFQKYAGQDGHGLTLSKTEFLNFMNTELGAFTKNQKDPCVLDRIMKKLDLNSDGQLDFPEFLNL
IGGLAQACHECFMNRVR

Supplementary Figure S2. Amino acid sequences of proteins encoded by EDC genes of the dugong. **(A)** Amino acid sequences of proteins encoded by SEDC genes of the dugong. **(B)** Amino acid sequences of dugong SFTPs. **(C)** Amino acid sequences of proteins encoded by other EDC genes of the dugong. The following amino acid residues are highlighted to show the peculiar amino acid compositions of SEDCs and SFTPs: lysine (K) and glutamine (Q) as potential sites of transglutamination; cysteine residues (C) as potential sites of disulfide bonds; proline (P). When available, the GenBank accession number is shown behind the protein name. Only the S100A proteins encoded by genes that flank *PGLYRP3* and *TCHHL1* are included here. SEDC, simple (single coding exon) epidermal differentiation complex gene; SPRR, small proline rich protein; SFTP, S100 fused-type protein; Dd, *Dugong dugon*.

A

>Em_LOR (XP_049731609.1)
MSHQKKQPTPTPPVGCGKTGGGGGGGGSVGGGCGGGSGSGGCDGGSGGGVKYSGGGGYSGGGYSGGGSSSCGGGGSGGSIKYS
GGGGSSGGGSSGGGFSCGGGYSGGGGGSYSGQQVPCQSYGGGSSGGGSSGGGLGCGGGGGYTGGSSSCGGGGSGYSLQQTTQT
PCVPLQSYGGGSSGGGNCGGGSSGGGSGCFSSGGGSSGGGSGCFSSGGGGSGSGCGGGSDGGKGVPVYNQTQQKQAPTWPCK

>Em_PRR9 (XP_049736471.1)
MSFNEQQCKQPCVPPPCLQKNQEQCQAKAEEVCLPPSQDPCQEKCPAQIQEVGLPQCQELSQENYPQQGQDPCLSPCQDQCLP
QCMEPCQELSQTKCVEIFPQNSQEKCSPPGKGK

>Em_LELP1 (XP_049736472.1)
MSSDDKSKSGEQKTEPKCEQKCEQKCEPKCQPSCLKKLLQLCSDKCPRDKCPSPCAPKCPPPCPPPCPPP
CPPPCPPPCPPPKLCAKPCPSKCPSPCPPPE

>Em_SPRR1L (XP_049736490.1)
MSYQQQQQRKVPSQPPPQVLEPCPPVVPVPCPPQVPEPCLPKVPEPFPQAPSQQKCPPVKCPPCQQEYQPKQK

>Em_SPRR1L2
MSYQQQQQCKVPSQPPPKVLEPCPPVVPVPCPPQVAQPCFPKVPELFPPPPSQQKCPPAKCPPCQQE

>Em_SPRR2EL
MSYYQQQCKQPCLKSPVVCPPKCPEKCPPVCCLPPPCPPVCCPPLPCQQKCPPVLPCQTCQQKCVPKFK

>Em_SPRR1AL
MSSYQQKQPFIPPPQPHDQQVKQPCIPPQDTFVPITKEPCYPQVPQPGNPKVPEPGYPKVPDQGQNKYPQPYPLPVTPGPDQQ
KTKQK

>Em_SPRR2EL2
MRLFCRFPETQERCLTISSSASNPACHLLWCAHLSALIHAHLCVALPPPCPPVCCPSPPCQQKCPPVLPPQHCQQKCVPKIK

>Em_SPRR2BL
MSYYQQQCKQPCLPPPVVCPPKCPDPCPPVCCPPSTMPTCVLPFSAMPAEMPSCVATPTLPAEVCT

>Em_SPRR2EL3
MLSCLASKSVTTGHTVMCLFSDSQRLRKDVLLSAAVQAALPCHLLWCAHLSALSHAHLCVALLRHAHLCVALPPPCPPVCCPS
PPCQQKCPPVLPPQPCQQKCVPKIK

>Em_SPRR2EL4
MSYYQQQCKQPCLKTPVVCPPKCPEKCPPVCCLPPPCPPVCCPPLPCQQKCPPMLPCQTCQQKCVPKFK

>Em_SPRR2EL5
MSYYQQQCKQPCLKSPVVCPPKCPEKCPPVCCLPPPCPPVCCPPLPCQQKCPPVLPCQTCQQKCVPKFK

>Em_SPRR2EL6
MSYYQQQCKQPCLKSPVVCPPKCPEKCPPVCCLPPPCPPVCCPPLPCQQKCPPVLPCQTCQQKCVPKFK

>Em_SPRR2EL7
MSYYQQQCKQPCLKSPVVCPPKCPEKCPPVCCLPPPCPPVCCPPLPCQQKCPPVLPCQTCQQKCVPKFK

>Em_SPRR2EL8 (XP_049736499.1)
MSYYQQQCKQPCLPPLVVCPPKCPVKCPPVCCPPPPCPPVCCPPPPCPPVCCPPPPCPPVCCPPPTCQQKCPPVLPPQPCQQK
CVPKIK

>Em_SPRR2EL9 (XP_049736498.1)
MSYYQQQCKQPCLPTPVVCPPKCPEKCPQLCCLPPPCPPVCCPPLPCQQKCPPVLPCQTCQQKCVPKFK

>Em_SPRR2EL10
MSYYQQQCKQPCLPIPVVCSPKCPVKCPPVCCPPPPCPPVCCPPPPCPPVCCPPPPCQQKCPPVLPCQTCQQKCIPKIK

>Em_SPRR2EL11
MSYHQQQCKQPCQTPPVVCPPKCPEPCPPPKCPDLCPPPKCPEPCSPLKCPEPCSPPRCPEPCLPV

>Em_SPRR2HL (XP_049731606.1)
MSYQQQEQCKQPCQPPPVCLPKCPEPCPPPKCPEPCPPPVKCPEPFPPIKCPEPCPPVQCPEPCPPVKCPEPCPPPEFPPQLC
QQKCPPVQPPQQCQQK

>Em_SPRR2EL12 (XP_049736481.1)
MSYQQQQCKQPCQPPPVVCTPKCPEPCPPPKCPEPCPPPKCPEPCPPPKCPPQPCQQKCPPTQIYQPCQQKCPPKSK

>Em_SPRR2GL (XP_049736497.1)
MSYQQQQCKQPCQPPPVVCPPKCPKPCPPPKCPEPCPPPKCPPQPCQQKCPPTQIYQPCQQKCPPKSK

>Em_SPRR2EL13 (XP_049736496.1)
MSYQQQQCKQPCQPPPVVCTPKCPEPCPPPKCPEPCPPPKCPPQPCQQKCPPTQIYQPCQQKCPPKSK

>Em_SPRR2GL2 (XP_049731605.1)
MSYQQQQCKQPCQPPPVVCPPKCPEPCPPPKCPEPCPPPKCPEPCPPPKCPPQPCQQKCPPTQIYQHCQQKCPPKGK

>Em_SPRR2EL14 (XP_049731604.1)
MSYQQQQCKQPCQPPPVVCTPKCPEPCPPPKCPEPCPPPKCPLQPYQQKCPPTQIYPPCQQKCPPKSK

>Em_SPRR2GL3 (XP_049736487.1)
MSYQQQQCKQPCQPPPVVCPPKCPEPCPPPKCPEPCPPPKCPEPCPPPKCPPQPCQQKCPPTQIYQPCQEKCPPKSK

>Em_SPRR2EL15
MSYQQQQCKQPCQLPPELYPPKCPEPCPPPKCLEPCPPPKCPLQPCQQKCPPTQIYQPCQQKCPPKSK

>Em_SPRR2GL4 (XP_049736495.1)
MSYQQQQCEQPCQPPPVVCPPKCPEPCPPPKCPEPCPPPKCPPQPCQQKCPPTQIYQPCQQKCPPKSK

>Em_SPRR2GL5 (XP_049736494.1)
MSYQQQQCKQPCQPPPVVCPPKCPEPCPPPECPEPCSPPKCPPQPCQQKCPPTQIYQPCQQKCPPKSK

>Em_SPRR2EL16
MSYQQQQCKQPCQPPPELYPPKCPEPCPPPKCPEPCPPPKCPLQPCQQKCPPTQIYQPCQQKCPPKSIHQDRARIRTIGSDSF
HNSTFTFFSKPVMDTDFLSICPGSAHNDS

>Em_SPRR2GL6 (XP_049736486.1)
MSYQQQQCKQPCQPPPVVCPPKCPEPCPPPKCPEPCPPPKCPEPCPPPKCPPQPCQQKCPPTQMYQPCQQKCPPKSK

>Em_SPRR2GL7 (XP_049736484.1)
MSYQQQQCKQPCQPPPVVCPPKCPEPCPPPKCPEPCPPPKCPEPCPPPKCPPQPCQQKCPPTQIYQPCQQKCPPKSK

>Em_SPRR2EL17
MSYQQQQCKQPCQPPPELYPPKCPEPCPPPKCPEPCPPPKCPLQPCQQKCPPTQIYQPCQQKCPPKSK

>Em_SPRR2GL8 (XP_049736483.1)
MSYQQQQCKQPCQPPPVVCPPKCPEPCPPPKCPEPCPPPKCPEPCPPPKCPPQPCQQKCPPTQIYQPCQQKCPPKSK

>Em_SPRR2EL18 (XP_049736492.1)
MSYQQQQCKQPCQPPPVVYLPKCPEPCPPPKCPEPCPPPKCPPQPCQQKCPPTQIYQPCQQKCPPKSK

>Em_SPRR2EL19
MSYQQQQCKQPCQPPPVVCPPKCPEPCPPPKCPPPEPCPPPK

>Em_SPRR2EL20 (XP_049736482.1)
MSYQQQQCKQPCQPPPVACPPKCPEPCPPPKCPEPCPPPKCPEPCPPPKYPPQPCQQKCPPTQIYQPCQQKCPPKNK

>Em_SPRR1BL (XP_049736489.1)
MSSQQQKQPCTPPPPLQQQQVKQPCQPPPQEPCVPQTKEPCHPKVPEPCHPKLPEPCQPTVTPAPPQQKTKQK

>Em_SPRR1AL2 (translation of ORF in pseudogene)
MSSHQQKQPFNHDLSLTTSR

>Em_SPRR1AL3 (XP_049736479.1)
MSSHQQKQPFTPPPQPHDQQVKQPCLSPQDTFVPITKEPYHPQVPQPGNTKVPEPGYLKVPDQGQNKYPQPCPSPVTPGPHQQ
KTKQK

>Em_SPRR1AL4
MSSYQQKQPFIPPPQPHDQQVKQPCIPPQDTFVPITKEPCYPQVPQPGNPKVPEPGYPKVPDQGQNKYPQPYPLPVTPGPDQQ
KTKQK

>Em_SPRR1B (XP_049736477.1)
MSSEQQKQPCVPPPQLQQQQVKLPCQPPPQEPCVPKVPEPCHPKVPEPCHPKVPEPCHPKVPEPCNPTVPDPCPPTVTPAPAQ
QKTKQK

>Em_SPRR4 (translation of ORF in pseudogene)
MSSQQQQQCPPQKP

>Em_IVL
MSQQHTLPVTLPAVLSQQPLKTVSPPLDTQQEQVKQPTPLPAPCQKVPSELPVEVPWEQGEKYTAPVKEVPEQEQGQQQHQEH
QQQEPQEQIQHQVQQLQQLQEQVQDQEQQQQEPQQQEQHQEQQQQEPQEQIQHQVQQLQQLQEQVQDQEQQQQETQQQEQHQE
QQQQEPQQQEQHQEQQQQEPQEQIQHQVQQLQQEQHQEQQQQEPQEQIQHQEQQQQEPQEQIQHQEHQQQEPQEQIQHQEHQQ
QEPQEQIQHQVQQLQQLQEQAQDQEQQQQETQQQEQHQEQQQQEPQEQIQHQEQQQQQPQHQEQQQQEPQEQIQHQEHQQQEP
QEQIQHQVQQLQQLQEQAQDQEQQQQETQQQEQHQEQQQQEPQEQIQHQVQQQQQPQEQVQDQEQQQQQEPQEQEQHQEQQQQ
QPQEPQVQVPYQEQKQEQQDQEQHQEHQESENLEQQLELENAQREQQLEEQLEQEKKLLGHQMDRELEKRDEQLGKKEEQLLE
HLEHQEMQPELSVQQEEQPVLIPAPDQVQETQPVQLPKGEVLFPTEHHQQKQEVQCPSKHK

>Em_SMCP
MCDQSKCNPCCQPPCQYCPPPRQCCPPPHQSCPPLHQCCPPPHHCCPSPSQCCPPPRQCCPSPSQCCPPPRQCCPSATQCCIK
QQCLEAKPESTCLNKESEPKPPQTQDRMSQSPQNASRPGTVGQQKKPSK

>Em_LCE7A (XP_049731602.1)
MSYQQSKQKCKLPAKCLPKCPPKCPPQAPQVPASCPAPCPHPAPSCCAPSYCISGFGGNCCLISYRFPRFYLRQPQHSGESES
SGCSSCCHDSGDCC

>Em_LCE6A (XP_049736488.1)
MSQQKQRPCELPDAPKCSPPQCPNPGFARCCSTCSGGYCLHSQRSGAQNPGRPRRTRRKPRCLRGGTIYHCKEEEC

>Em_LCE1EL (XP_049731601.1)
MSCQQSQQQCQPPPKCTPKCPPKCPTPKCPPKCPPKCPSASSCCGSSGDWCSSGGGGCCLSHHRHRRSCRHRHHSSECCSQPS
GGSGGCGGSSCCGGGNGQSSGGSCC

>Em_KPRP
MCDQQQIQSSLPLPQCCVKDSSFFPSPYPCATGEVVVQAPCEMQIVECPVPCPVQVSQVKCQAPCQSKTTQVKCQAPCQAQET
QVKSQAPCQSEVSYVQCETPCPVQTCYVEHVPVCCTETCYVEYPVETYVPCPAPQPVQTYVAYSPVCQNQEGFRIQGQYQGSY
GSCVPQRRSRTSFRICAPQCQTQGSYGSFTAQRRSQSASRCLPPPPLRPSYRSCSPPPRCGPFYSSCLPSRCSWDSYNYCTPP
RRSEPIYNSGCARDRASGCSQRCRPKCRIEISSPCCPKQVPLQKCRVQIPPISRCPQTCTPRFSSGASCPDLRPRELPRSSFR
PLRRLDQCPEPLLPLCPLPAPRPYPRPCPLPAPRPYPLPCPLPEPCLRPEPRPCPPRRRLSEPCLRPAPRPTPRPRPVQCERP
EPRPYPRPCPLPEPIPLPAPCPSPEPCVEPWSYPNPCSGPNPIPCQGDLGCHESSPCHLDTEVPSYGPASYNPGRE

>Em_KPLCE (XP_049731600.1)
MCDQQEQQRFPPTCVKGLRLGSVQSTKCTSVKCAVPCETKTVSVVCPDPCQTQTYVKCPVPCKTTCVKCPPPCQTQTYVKCPV
PCQTTYVKCPTPCQTQGYVKCPPPCQTTYVKCPPPCQTTYVKCPAPCQTTYVKCPVQCQMTCVKCPVPCQTTYVKCPVPCQTT
YVKCPTPCQTQTYYVQCPSPCQTYYVQAPASSRVQSSGSQGCNP
DPCCDCGCCCLGIIPMSSRGPACCDLEDDDCCC

>Em_KPLCE2
MCDQQEQQHFPPSCVKGLKVGSVQSTKCTSMKCAAPCKTKTVSVVCPDPCQTQTYVECSVPCQTT

>Em_LCE1EL2
MSCQQNQQQCQASPKCATKCPPKHPPVSSCCSISAGGCCGSSGGCCGSSSVRCCSSGGGGCCLSHHGHPRSHHDRHQSSDCCG
SSQHSRGSDRCSGGSTWCSGGCC

>Em_KPLCE3
MCDQQEQQHFPPSCVKGLKVGSVQSTKCTSMKCAAPCETKTVSVVCPDPCQTQTYVKCSVLCQTMCVKCPTLCQTQTYVKCPA
ACQTTYVKCPPPCQTQSYVNCPVPCQTTYVKYPAPCQTTYVNCPVPFQTTYVKCPVPCQTTYVKCPTPCQTQTCYVQCPSLCQ
TYYVQAPASSTVQSSGSQGCSPDPCCHCGCCCLGIIPMSSRGPA
CSDLEDDDCCC

>Em_LCE1EL3
MSCQQNQQQCQASPKCATKCPPKHPPVSSCCSISAGGCCGSSGGCCGSSSVRCCSSGGGGCCLSHHGHPRSHHDRHQSSDCCG
SSQHSRGSDRCSGALLGALGAAADLDHEIH

>Em_KPLCE4 (XP_049731598.1)
MCDQQEQQYFPPTCVKGLKVGSVQSTKCTSVKCAAPCETKAVSVVCPDPCQTQTYVKCPAPCKTTCVKCPTPCQTQSYVKCPP
PCQTTYVKCPTPCQTQSYVKCPPPCQTTYVKCPAPCQTTYVKCPAPCQTTYVKCPVPCQTTCVKCPVPCQTTCVKCPVPCQMT
CVKCPVPCQTTCVKCPVPCQTTYVKCPTPCQTQTYYVQCPSPCQTYYVQAPASSRVHSSGSQGCNPDPCCDCGCCCLGIIPMS
SRGPACCDLEDDDCCC

>Em_KPLCE5 (XP_049731596.1)
MCDQQEQQHFPPSCVKGLKVGSVQSTKCTSMKCAAPCKTKTVSVVCPDPCQTQTYVECSVPCQTTCVKCPTPCQTQTYVKCPA
PCQTTYVKCPPPCQTQTYVKCPAPCQTTYVKCPVPFQTTYVKCPVPCQTTYVKCPTPCQTQTCYVQCPSPCQTYYVQAPASST
VQSSGSQGCNPDPCSDCGCCCLGIIPMSSRGPACCDLEDDDCCC

>Em_KPLCE6 (XP_049731595.1)
MCDQQEQQRFPPSCVKGLKLGSVQSTKCTSMKCAAPCETKTVSVVCPDPCQTQTYVKCSVLCQTMCVKCPTLCQTQTYVKCPA
ACQTTYVKCPPPCQTQSYVNCPVPCQTTYVKYPAPCQTTYVNCPVPFQTTYVKCPVLCQTTYVKCPTPCQTQTCYVQCPSLCQ
TYYVQAPASSTVQSSGSQGCSPDPCCHCGCCCLGIIPMSSRGPA
CSDLEDDDCCC

>Em_LCE1EL4
MSCQQNQQQCQASPKCATKCPPKHPPVSSCCSISAGGCCGSSGGCCGSSSVRCCSSGGGGCCLSHHGHPRSHHDRHQSSDCCG
SSQHSRGSDRCSGGSTWCSGGCC

>Em_KPLCE7
MCDQQEQQYFPPTCVKGLRLGSVQSTKCTSVKCAAPCETKAVSVVCPDPCQTQTYVKCPAPCKTTCVKCPTPCQTQSYVKCPP
PCQTTYVKCPTPCQTQSYVKCPPPCQTTYVKCPAPCQTTYVKCPAPCQTTYVKCPVPCQTTCVKCPVPCQTTCVKCPVPCQMT
CVKCPVPCQTTCVKCPVPCQTTYVKCPTPCQTQTCYVQCPSPCQTYYVQAPASSRVQSSGSQGCNPDPCCDCGCCCLGIIPMS
SRGPACCDLEDEDCCC

>Em_LCE2AL (XP_049736475.1)
MSCQQSQQQCQPLPKCPPKCPPKCPTPKCPPKCPPKCPPVSPCCSGSSGASSCCLSHHHRPHLFHRHRHQSPDCCECEPSGGS
GCCSDSGGCC

>Em_LCE2AL2 (XP_049736474.1)
MSCQQSQQQCQPLPKCPPKCPPKCPTPKCPPKCPPKCPPVSPCCSGSSGAGSCCLSHHHRPHLFHRHRHQSPDCCECEPSGGS
GCCSDSGGCC

>Em_LCE2AL3 (XP_049736478.1)
MSCQQSQQQCQPLPKCPPKCPTPKCPPKCPPKCPPVSPCCSVSSGAGSCCLSHHHRPHLFHRHRHQSPDCCECEPSGGSGCCS
DSGGCC

>Em_LCE2AL4 (translation of ORF in pseudogene)
MSHQQNQQCQPPSKCTPKCTPKCPPPNAQLYTQPLPAVAALGEAVDLGVAAAV

>Em_LCE3CL (XP_049731966.1)
MSCQQNQQQYQAPPKCPSPKCPPKSPAQCLPKVSSGCALTSGGCHGPSSETGCCLRHHRRRRSHRCRRRSSNCSDDGSGQQSG
GSHCGHSSGGCC

>Em_LCE3CL2 (XP_049731965.1)
MSCQQNQQQYQAPPKCRSPKCPPKSPAQCLPKVSSGCALTSGGCHGPSSETGCCLSHHRHHRSHRCRRRSSNCSDDGSGQQSG
GSHCGHSSGGCC

>Em_LCE3CL3 (XP_049731964.1)
MSCQQNQQQCQAPPKCPLPKCPPKSPAQCSPKVSSGCALSSGGCHGPRSEAGCCLSHHRRCRSHRCQHQNSDCSDNGSGQQSG
GSRCDHSSGGCC

>Em_LCE3CL4 (XP_049731963.1)
MSCQQNQQQYQAPPKCPSPKCPPKSPAQCLPKVSSGCALTSGGCHGPSSETGCCLRHHRRRRSHRCRRRSSNCSDDGSGQQSG
GSHCGHSSGGCC

>Em_LCE2AL5
MSCQQNQQQCQYPPMCPPKCLPQCPAPCPPSVSSCCCSSSGGGSCYLSHHGYGLFLRHLHQSPTCCECEPFWGLRLLPQLCWL
LLM

>Em_LCE3CL5 (XP_049731594.1)
MSCQQSQQQCQAPPKCPSPKCPPKTPTQCLPKVSSSCALSSGGCHGPSSEAGCCLSHHRRHRSHRCWRQSSNCSDDGSGEQSG
GSHCGHSSGQCGRDKQL

>Em_CRCT1
MSSQQSSKSFSKGSSQGSAPCPAPTPAPSPSSSSCCGGGCCGDSGCCGDSGCCGSSSTGCCCFPLRRRRQRRGCCCCGGSSQR
SQCSSNSGSSCCC

B

>Em_CRNN
MPQLLRNIHGIIEAFGRYAKTEGNCMVLTRGELKRLLEHEFADVIVKPHDPVTVDEVLRLLDEDDTGTVEFKEFLVLVFKVAQ
ACFKTLSESPEGVCGSQESRSCHSAASQELREGQRSHTEVGGAGRGQLHEGSSRGQSKQASTGQDGSSTQVTHHDRQAESQIQ
ESMSQEAQETGQTQSAEDKSHQTREGQSERQSQTREQDRAHQTSETVTGTLTQTQTGATQTPGQDRSHQTGSTGSQARESNYS
QTRGTHSQDRSQTSQIVTGGHIQTQAGSQTQTTEQDRSHQTGNTSTQSWESTIGQTRGTHSQNRSQTSQGVTGGYVQTQTGSQ
TQTVEQDRSQQTGNTSTQSWVSSFHQTRETHDQDRRQTSQTATGGHIQTQAGSQTQTHTQAPGQDESHQAGSTITQSRESNTG
QTRGTRSQDRSQTSQGVTGGYVQTQTGSQTQTGEQGRSQQTRNTSTQSRESTVGQIRGPHSQDRSQTSQTVTGGHVQTQAGSQ

TQTTEQDRSHQTGNTSTQSQESTIGQTRGTHSQNRSQISQGVTGGYVQTQTRSQTQAVEQDRSQQTGNTSTQSRESTIGQTRG
AHSQDRSQTSQTVTGGHVQTQAGSQTQTTEQDRSHQTGNTSTQSRESTIGQTRGTRSQNRSQTSQGVTGGYVLTQTRSQTQTV
EQDRSQQTGNTSTQSRVSTSGQTGATETNSQDGNQKSQVVTGGYIQTQAGSQTQVHPQTMEQDRSQMGSHVGAREQEQSQTQS
GSGQTWTQVGNYEAGETVLEGQAQPGASTLTGSQDWSSSHPSCSVTARQGEGEPHPKESNVVVEEWVDDPTREMVVQRQNQSS
LHTSVSSAQGGETAQPEGR

>Em_FLG2
MTDLLRSVVTIIDIFYKYTRQDGECGTLSKEELKELLEKEFHPILKNPDDPDTVDVIMHMLDRDHDRRLDFTEFLLMVFKLAM
ACNKVLSKEYCQASGSKKHRHGHQHQEEESETEEEEEETLGQKSGHRYSSWSEREEHGYGSGGSSGTVKHRRGSKSRRLGRRD
GLSSSENEEGSEKRSHGSHSGHFWSSGKERHGSRSEELGERRNKSSVSPSRESGEEYEYGSGSKSRGRKAHGGLSHGLEASGH
ESSSTQSRSGGQSLRSSSGGSGDCGRQSHTCNSSNSEGYGRSQNASSSCQAGRFGGLGNQSSCTQSGYESGSSEGQGHEGISG
GQSSGCQFSGYSQHNSGSSSQSSSHRGHESRARGQPQNSRGQQQGTGSSQSSCYEQYGSGVSQSSSYGQYEYGSCGSSSTSSQ
KRSGSNEFSKRGQLDPGSGHFSSQHRSSSSQSSGQHGSGPSQSSYGQHGSGSHQSSGQHGSFTGQSSSQHRSSSSQSSSQHGS
GTAQFSGQHGSGSSQSSFGQHGSGSGQSSGFGQHGSGLSQSSYGQHGTSSSQSSGQLGSCTGQSSSQHRSSSSQSSGQHGSGT
GQFSGQQGSGSSQSSFGQHGSGSGQSSGQHVSGSSQSSYSHHGSGSSQSSGQHLSGSSQSSYGQHGSGSSQSSGQHGSCTGQS
SSRHRSSSSQSSGQHGSGSSQSSGFGQNGSSSSQSSGQHGYGIGQSSYGQHGSQSSQFSEQHGSGSGQFSGFGQHGSGSGQSS
GFKQYESRSSQSSGQHRSGSCQSSRQQGYGSSQSSYGQHGCRSSQSSGQHGPGISQSSYGQHRSSSSQSSGQHGYGSSQSSGF
SQHGSGSGQTSGFGQHELGSRQSSHSGQHSSGSGQSPSFEPQGSSSGKSSSSGQHRSSISGKGSCGPISGKNYPKSGRTISDS
SHSQARSSSQERTKQGDSERSQGHSISGSESEDHDSTHQHRQQRQGSNEEGRVPGRQETRYSQTSDSEGQSDNWERYSASPHT
SSRIDHEVPSHEKSGSSRRQQSSQRQSSDSEQHSGSSQRHSGSTHGQSGSQHGESGSSRRKGSSHGHSGDTTRHDQSGHGQLT
QSGSRTTERRDSSHSQSSDSEWQSEDSQRHSGSTHGQSGSQQGESGSSRRQGTSHEQSGDITRHASSGHGQLTESVSGTTGRR
GSSHSQSSDSEQHSGDSQRHSESTHGQSGSQHGESGSSRRQGSSHGHSGETTRHDQSGHEQLTQSGSRTTERRDSSHSQSSDS
EWQSEDSQRHSGSTHGQSGSQQGESGSSRRQGTSHEQSGDITRHASSGHGQLTESGSGTTGRRGSSHSQQSSDSEQHSGDSQR
HSESTHGRSRSQHGESGSSRRQGSSHGQSGETTRHANSGHGQLTQSGSRTTGRRDSSHSQSSDIEQHSGNSYRHSRSGHDQSG
SQHGESGPSRRQENFHGQSGDTTKQAQSSHGQRTESGSRTAERRDSSHSQSNDSEWHSGVSERHTASTYGQSGSSSKQSVSNS
TRTYESSNEHSEDIPKHSVSHQRQTSPYGQPGYRTSGRQGSPHVQRGDRSTHSGSIHGQSGSRTTGRQGSTQSHDIHRQSRDS
IRTQSSSSHHQPGANHSQPWSSQRQRHGSGQGWKHGSYGRAAYDYGESGYGPSGGSRASNHNSNQEQRCYYFD

>Em_FLG
MSTLLENITAIIDLFQQYSSNDKENDTLSKKELEELLETEFQPILENPNDPDTADDFMHILDLDHNKKVDFTEFFLMVFKLAQ
AYYESTKRQNFKASGPKQKKYGHKCQNEEDEVEEEEKIEEQRRKSSLSRRHRQTGKKSLTTTDKKKHRSSSKNKGRRDDSSPS
GHREKNGKNHHHHTGKNKRSSFTDLGEETNISSISQKREVGDEHNYGSENRERGSARWEYSNQEESEDSDRQAGPPGTNHNGF
THHQSRGRRHSGFNQEHRSDSQQLHSTHKHSHSAARGRQGSRHGHSVDSSTHSDSDQGEISSEGRSRSSTKSRQQSHQHNSAD
HPRHSGTGRGRSTIESGAGSHRGSSVGQASNSEGQSEDSQRQSGSASGHHHGSNQGQSRDSSRHSGYHQGHRTTTPHSHSAHG
HSDSTPIESQGPPYGQSGDSSRHSGSHQGETSAHGQSRSSTRGRQKSRQEHSADRSRRSGPGHAQSSVESGEGRHRGSSVSQA
SDSKGQSEDSHRQSGYASTHYHGSTHGQSRDTSRHSGSHQGHRADASQSHSAHGHSESTARGRQGSSHGHSVDSSRHSSSHQG
ETSAHGQPRSSTRGRQEYHQEHSADHARQSGTGHGRSPNESGAGRDRGSSVSQASDSEGQLENSHRQSGYASRHHQGSTHGQS
RDYSGHSGSHQGHRADASQSHSAHGHSDSTARGRQAYSHRQSVDSSRHSGSHQGETSAHGQSRSSTRGTQESPQEHSADRSTH
SGAGHTHSSVESGEGRHRGSSVSQATDSEGQSEDSHRQSGYASTHYHGSTHGQSRDTSRHSGSHQGHRADASQSHSAHGHSDS
TVRGRQGSSHGHSVDSSRHSSFHQGETSACGQPRSSTRGRQESHQKHSGERSRDSGTGRGQSPNESGAGRHREFSVSQASDSE
GQSDDSQTQSGYASRHHQGSTHGQSGDNSRNSRSHQGHRDDGPQSHSAHGHSDSTATGRQGSSHGQSVDSSRHLGSQQGETST
HRQSRSSTRGRQESHLEHSTDRSRHSGAGHAQSSFESRAVRHRAPSVSQASDSEGQSEDSHRQSGYASTHYHGSTHGQSRDSS
RRSGSHQGHRADRPQSHPAHGHSDSTARGRQGSSHGQSVDTSRHSGSHQRETSAHGQSRSSTRGRQESRQEHSADHSRHSGDG
RGQSSIESGEGRHRGSSVSQASDSEGQSEDSHRQSGSASGHHHGSNHGQSRDSSRHSRYHRGRRATAPNSHSAHGHSDSTAIG
SQGPSYGQSVDSSRHSGSHQRETFTHGQSRSSTRGRQESRQKHSADHSRHSGTGHGQSPNESGVRRHRGSSVIQASDSKGQSE
DSQTQSGYASRHHQGSTHGQSRDNSRHSGSHQEHRADGPESHSAHGHSDSTARGGQGSSHGQSVDSSRHSDSQQGKTSAHGQS
RSSTRGRQESHLEHSADRSRHSGAGHAQSSFESRAVRHRGPSVSRASDREGRSEDSHRQSGSASTHYHGSTHGQSRDSSRHSG
SHQGHRANGPQSHPARGHSDSTARGRQGSSHGQSVDTSRHSGSHQRETSAHGQSRSSTRGRQESRQEHSADRSRHSGAGYAQS
SLESGAVRHRGSSVSQASDSEGQSEDSHRQSGSASGHHHGSNHGQSRDSSRHSGYHRGHRATALNSHSTHGHSDSTAIGSQGP
SYGQSVDSSRHSGSHQRETFTHGQSRSSTRGRQESRQEHSADRSRHSGDGHGQSSAESGEGRHRGSSVIQASYSEGQSEDSHR
QSGYASTHYHGSTHGQARDSSRHSGSHQGHRADASQSHSAHGHSDSTATGRQGSSHGQSVDSSRHSDSQQGETSAHGQSRSST
RGRQESHLEHSTDRSRHSGAGHAQSSFESRAVRHRAPSVSQASDSEGQSEDSHRQSGYASTHYHGSTHGQSRDSSRRSGSHQG
HRADRPQSHPAHGHSDSTARARQGSSHGQPEDSSRHSGSHQGETSARGEYRSSTRDRQESLQEHSADHSRHSGAGNAQSSFES
GAGRHRGSSVSQASDSEGQSEDSHRQSGFASTQYHGSTHGQSRDSSKHLGSHQGHRANGPQSHSVHGHSDSTARGRKGSSHGQ
SVDSSRHSGSHQGETSAHGQSRSSTRGRQETQQEHSADHSRHSVSGHGRSSFESGAGRHRGSSVGQASDSKGQSEHSGIQSGS
GSGQQGRSTYSQSRDSSRHSGSPQGLRADAPQSHTAHGHSYSSAIGKQGSSQGQSLDSTRHSGSHHGETSAHGESRSSTRGKK
ESRQEHSADTSRQSGTGRGRSTIESGAGRHGGSSVSQASDSEGQSEDSHRQSGSASGHHLEFNHGQSRDSSRHSGYHQGHRAT
VPQSHSAHGHSDSTAIESQGPSYGQSGDSSRHSGSHQGETSAHGQSRSRTRGRQESRQEHSADRSRHSGAGHAQSSLESGAVR
HRGSSVSQASDSEGQSEDSHRLSGYASTHYHGSTHGQARDSSRHSGSHQGHRADASQSHSAHGHSESTARGRQGSSHGYSVDS
SRHSSSHQGETSAHGQPRSSTGGRQKSHQEHSADHSRQSGTGHGRSPNESGAGRDRGSSVSQASDSEGQSEDSHRQSGYVSRH
HQGSTHGQSRDYSRHSGSHQGHRDDGPHSHSAHGHSDSTARGRQGSSHGQSVDSSRHSDSHQGESSAHGQSRSSTKGRQESHQ
ERSADRSRHSGAGHAQSSFESRAVRHRGPSVSRASDREGRSEDSHRQSGSASTHYHGSTHGQSRDSSRHSGSHQGHRADGPQS
HSAHGHSDSTARGRQGLSHGQSADSSRHSDSHQGETSTHGQSRSSTRGRQESRQEHSADRSRHSGAGYAQSSLESGAVRHRGS
SVSQASDSEGQSEDSHRQSGYASTHYHGSTHGQSRDSSRHSGSHQGHRADASQSHSAHGHSESTARGRQGSSHGYSVDSSRHS
SSHQGETSAHGQPRSSTGGRQKSHQEHSADHSRQSGTGHGRSPNESGAGRDRGSSVSQASDSEGQSEDSHRQSGYASRHHQGS
THGQSRDYSRHPGSHQGHRDDGPHSHSAHGHSDSTARGRQGSSHGQSVDSSRHSDSHQGESSAHGQSRSSTRGRQESHQERSA
ERSRHSGAGHAQSSLESGAVRHRGSSVSQASDSKGQSEDSHRQSGYASGHQLESNHGQSRDSSRHSGYHRGHKATAPNSHSVH
GHSDSTAKGSQGPSYRQSVDSSRHSASHQGETSTHGQSRSSTRGRQESHQEHSADRSRHSGAGHARSSIESGAGRLRGSIVSQ
ASGTEGQSEVSFGQSASNHESSGFGSRNQPGSIHGQSGDSLRHSGSHSGQISTQRQSDSTHVHQSQKADHGQSDSNGRINQGS
SLCQFYSVNNDRQRHDAGHCWRHGSYGSSDYDYGQSRFGQS

>Em_HRNR

MPKLLPSIVSVIEIYYQYATEEGECNSLNRAELKELLENEFRQILKNPDDPDTVDIIMQSLDRDHNKKVDFTEYLVMIFKLAQ
ACNKIISKDYRQTSGSKQRHHSYQHQEEQSETEEEEKGQESSSSHSSWSTGEENDSYSRDSRSIKHKTGSSSRRLGYQGGLSS
SEHRQSSGERRESSSGYSKGRGKNKHGSYQPEGSESEEVGSTHSSNHRKRSNSANKSDSCGEQEHISYSEDQSFSFDQHWSDS
NESLGNRQHRRRSDKFPSKQHHGFSSSSCGGQDSWTNSNESTDYGQLGFGSGQPSQQRQHEPNAGSQSGKCEEQGYHSSSSES
SSYGKHRSSSGQPSSQKKRGSSSSGQSGNWRKEKHGSGSGKSSSFEQYGSSSGQSSNCGKHGSSINHSSSQEQHKSSSSGQSG
HKGQQKQSGFQSGQSSSYEECKSESGQSLGSGKNQPSSFQSSTQRKQSNSSGRQSESCGRQKHGSGSVQSSGCGQQGSGTKQS
SSYGHHGSGSGHSSNHAQYETGSGQSSSHSHQKCGSGESSGFGQYGSSSSHSSRLKHHEYNSGGHSGSCGRQKHGSGSSQSPN
HGKYGSASNQSSSQHGFGSGQSSSYEQQRSGSQQSSSYRQHRFGSGQSSSCSQHGSGLGQSSSCGQHGSGSGQTLSHSRHGSG
SGQSSSYGQHESCSGQSSSFGQHGSGLGQSSGCSQHRSGSGQSSSYEKHWSGLGQCSSHSKHGSSSGQSSGFGQHGSGSVQSS
SYGQHRSGSGQSSSYGQHGSGSGQSSSHSRHGSGSRQLSNCGQQGSGSSQSSSYGQDGSGSGQSSSYGQHESGSCQSSSYSQH
GSGLGHSSSHGQNGSSSGQCSSYSQHGSGSGSGSGSGSGSGSGSGSGSGSGQYSNSEQYGSGSCLSSCSEQYDFGSGQHSS
YEQRGQVESSSGCGFGQFTTSYGQSRFNTANKLSICNEVYRQSGNCYQRGSNCGRGSTDSISHSFCSSTPLYEYVQERNNKQK

>Em_RPTN

MAQLLSSILTVIKVFQKHASENGDCTSLCKKELKQLLLAEFGDILWRPNDPETVETILTILDRDSNGHIDFHEYLLLVFQLAQ
ACYHKLDIQSCGDRTSQQEEEQEGVQDHTFPRNRGRQHRQRHEEERQHSHHGHSERQDRDSHHSGSERQERDSSYGQSERQDQ
DSYYDQSERQERDSHYGQSERQGWDSRHGQSERYNLDSHYSQPERQDRDSSLNQSERQGQDSSYGQKLSHKSRQGYLFALNQC
EKPVQDSHHNQSESLGQRSSCGQSRRLGQDSCSSHTEQQQTGCIYGQSGRLDQESGCGQKHRQGLDSEYGQTDRQDQSPHYCQ
TGRQGQSSHYGQTDRPGQSSHYGQTDRQDQSSRYGQADRRGQSSHYGQTDRQGQSSDYGQTDRQGQSSCYGQRDRQGHSSRYG
QADRRGQSSHDGQTDRPGQSSHYGQTDRQDQSSHYGQTDRQGQSSHDGQTDRQGQSSHDGQTDKEVHSSHYGQTETGETEIQG
QDRHFQGTEGTRRDTYVEQSGRSGRLSQQTQGQEVNRNQRQGSQNRQQAWKRQLEDTQHHQHKLLAQIQQERSLCHKGRDWQS
SGSEQGHRQAETQQSHSQTEERQGHQSWGIHSHEGQESPREARDRQTHKEEQSCQTRDRQSHEDEQNHQRQDRQTHEDEQNHQ
RQDRQTHEDEQNHQREDWQTHEGEQDHQRQDRQTQEDEQNRQRQDRQTHEGEQNHQRQDWRTHEGEQNRQRQDRQTHEDEQNH
QRQDRQTHEDEQNHQRQDWQTHEGEQDHQRQDRQTHEDEQNRQRQDRQTHEGEQNHQRQDWRTHEGEQNRQRQDRQTHEGEQN
RQRQDRQTHEGEQNRQRQDRQTHEGEQNRQRQDRQTHEGEQNRQRGNRQIHEEDQNCWQQQNTQTHEGEERYDRSQNQQFNVT
QQGCSNRETFHLNEGGQSRVSQGRRSDQAFHSTQGNGRPQRREQDRDHPTKAAISSNPLYDYVQEQRSHQY

>Em_TCHH

MPALLRSICDITEIFNQYASNDCDRAALCKKDLKELLEREFGDVLRRPHDPETVDLVLELLDRDHNGLIDFHEYLLLIFRMAQ
ACYYALSQATGLDEEKGAKYEGKRNPLNDRRQEDQRRFERRQRLVEEERKQNGQERELVEEELQRERQQRREQRDPQRRDEGQ
RLQRREGLERREEKGLLQRRQGREPAEFPEEEQLQRRERLEQRERQVEEEQLQRERQVEEERRQRRERLEQELQLPRRGRDEP
RAQRERREQERREQELAEEEQQELGESRASRWQWQPESEADARHSKVRSRPGRQEEQRRRLEQEEESRRCQEPEQQLRRELED
DARRQARPGQLREERERRDFQWQLEEEREKRRQRLSARSSLKEQRERQARAKEGQERDGRLGEEEDQRRDREELQFLEEEEQL
QRRRRAGQFQEEGNLQEDQEIRRRQEDQGRDQKWKWQLEEESQRRRHLLYSKSAKQEQLQRQARDRQYGEEDLLQQEEEQLQR
EQREKRRRQEPERQYREEEQLQQGEEQLQRERRLQERDRKYREEEEPQRQERKQQFRDEDQCRDLKRQPEKENEVRKNRENEE
KARQLEESQVLERQFQQDRRPQQDELEEELSLQEREQERRRWLQRERQFPTEVPLRGEEQEKAKRLDGKFREEEQLLREGTEE
KRRRQEGDRRFPEESFQQSRDRRFQEEEKLRRQERDRKFREEEQRLQEREEEQVRRQLRDRQFREEEQQLDQELEEERLRRQQ
RDRNFREEGQMRLEREEQQLRRQQQDRNFPEEEQLRQERENQQLRRQQRDRKFLEDEQLSQEREDEQLRRQERDRKFREDEQL
RRQEREQQLPQERDRKFLEEEELRQEREEQQLRRQEREQQLRQERDRQFREVEELRRQQREQQRRQERDRQFREVKELRQERE
DEPLRRQQRDRKFREDEQLRQEREEQQLRQERDRQFREVEELRQEREDEQLNRQQRDRKFREDEQLLQEREEQQLRRQEQEQQ
RRQELDRQFREVEELRQEREERQLRRQEREQQLRRERDRQFRDVEELRQEREDEQLNRQQRDRKFREDEQLLQEREEQQLRRQ
EQEQQRRQERDRQFREVEELRQEREERQLRRQEREQQLPQERDRKFLEEEELRQEREEQQLRRREREQQLRQERDRQFREVEE
LRRQQREQQRRQERDRQFREVKELRQEREDEPLRRQQRDRKFREDEQLRQEREEQQLRRQEQEQQRRQERDRQFREVEELRQE
REERQLRRQEREQQLRRERDRQFREVEELRQEREDEQLRRQQRDRKFREDEQLLQEREEQQLRRQEREQQLRQERDRRFREVE
ELRRQQRDRKFREDEQLRRERDDEQLRRREREQQYRQERDRKFREEEELRQEREEQQFRRQEQQLRQERDRKFREDEQLRQER
EKEQLHRQEREQQLRQERDRNLREVEELRQEQEDEQVRRQQRDRRFREEDELRQEQEELQLSRQERDRRFREEQQLRQEGEDA
QLRRQELDAAFSQDEQLNRAEQEEEQRRWRQKGSKFLEEEESLQQEREEEKRRRREQDRKFRQEEEQLRREEQQEQRHRQERD
RQYRAEEQFARDKRRRQEQELRQEEEQRRRQERERKLREEDQGKRQFVNPPVRSSPLYEYIQEQRSQYRP

>Em_TCHHL1

MPRLLRRVLCVIETFHKYAREDGDGVTLTHRELKQLLQGEFGDILQPHVMHAVEKNVNLLDIGSDGTIRFDQFVLATCNLLNH
CYLDIQSLNSEPRQAPKPEKENPGDVDPQATSRSGQLTEETPPTQDKVVLPSGMAPSSQLNPEEREPVEHNRVDPQEDSKTHN
LPGEASEHNDSKNQHLEGDEQIQEVAQHVQTAGDNGAQLETNKEMVTSKQTSSPTEVEGQDKEIPREAEKPAGEQSHTKARDQ
LGEQEGNLGTQSSPAEETAQRPSKDHKVATEKGIKEHSKTQEPSLQAENESSSEQADLPEKGAEGKQSPTQKLTDPEDEGRIS
ETQEPGKDANRESYESEKLTESVIDSRVPEVQEPGKGDADRTPPETKITAEPGDDGRISETQELPAQEQETKDMPVQGDGKNI
SETHYVTTERKLGRGPETHGTTGQKGSKRKTQTPALEVQTQEGMYQELQGPSKERDTAKGTETQDLGSEGTDQSHTEIEGAAA
LGDVRYIEKGREEALVGSKNAPVAEGTAGARERTQESAPLESQSVGKKRRITKTQDKSTKEDDSYQGEDPVPPTTQNDEGFFK
TPNNLAPEEGDSSLETGDLPAQRDSQSQVDPPGESVQGGCNNDPDAQKQAESGEKYRAQEAVVLAVRGEAEQLTDEQEQFLRG
EHKSWGSGTKGPDPDVEVNGLPEAQQSTAGSDNGKSVKAEIPGALDADCNDQPSVMQQPAKGDSRKKLKVQGPGTKGEKGGES
KTQETPLKSLNEDNSASSETHIEEPATLKEEDENPQELAEGDDQQHPTKKGGYSSAPLPGLEERMQRDQQPYTVEKDLVHPSP
SYTYLQEKIPQQIDITHEERQNQAQPAQASGPELSTDQSRASLTSEISNCLTIFYHSQALRRYTRELSPDEAPADPQQTSAL

C

>Em_PGLYRP3 (XP_049736469.1)
MLPWLLVFSVLGLVAGDAPAIVSRNEWGSRMFACSAPLTHPVAYVITDELRGMECQEQNICSWKLRGLQSHSVYTKGWCDVPY
NFLVGDDGRVYEGVGWKIQGLHTQGYNNISLGLAFFGNKMGSSPSPAALSAAGGLISYAIQKGYLSPRYIQPLILQSETCLVP
QQPARSRKACPNVITRPAWGARETLCSKMDLPVKYVIIIHTAETSCNVSVDCQIRVRDIQSFHIDDKNFCDIGYQFLVGQDGV
VYEGVGWHTQGAHTYGYNDIAVGIAFIGNFVEKPPNDAALEAAQNLIQCAVDKGYLIPNYLLVGHSDVVNTLSPGKALYNIIK
TWPHFKH

>Em_S100A9 (XP_049736470.1)
MADQLSQLERDIETIINVFHQYSVRLEHRDALNQKEFKQLVQKELANFLKKEKKDDAVINDIMEDLDTNEDKELSFEEYIVLV
AKLTEASHEKMHEGHPRGHHHSHGEGLGEDRKCKGDDKQKGSGPGQGHGHSHGHGHSHGHGHSH

>Em_S100A11 (XP_049736463.1)
MAKISSPTETERCIESLIAVFQKYAGQDGKNLTLSKREFLNFMNTELAAFTKNQKDPGVLDRMMKKLDLNSDGQLDFQEFLNL
IGGLAQACHDSFMRRPH

Supplementary Figure S3. Amino acid sequences of proteins encoded by EDC genes of the elephant. **(A)** Amino acid sequences of proteins encoded by SEDC genes of the elephant. **(B)** Amino acid sequences of elephant SFTPs. **(C)** Amino acid sequences of proteins encoded by other EDC genes of the elephant. The following amino acid residues are highlighted to show the peculiar amino acid compositions of SEDCs and SFTPs: lysine (K) and glutamine (Q) as potential sites of transglutamination; cysteine residues (C) as potential sites of disulfide bonds; proline (P). When available, the GenBank accession number is shown behind the protein name. Only the S100A proteins encoded by genes that flank *PGLYRP3* and *TCHHL1* are included here. Em, *Elephas maximus indicus*; ORF, open reading frame; SEDC, simple (single coding exon) epidermal differentiation complex gene; SPRR, small proline rich protein; SFTP, S100 fused-type protein.

```
                   intron      G  D  S  S  W  N  K  T  Q  A  K  Q  V  S  E  G  L
Human    PGLYRP4   ATTCCTTTGCAGGTGATTCCTCCTGGAACAAAACACAAGCTAAACAGGTATCAGAGGGGCT
Dugong   PGLYRP4   GTTCTTTTGCAAGCGATTCCTCCTGGAATGAAATACAAAC-AAAGACATATCAGAGAGGCT
Manatee  PGLYRP4   GTTCTTTTGCAAGCGATTCCTCCTGGAATGAAATACAAAC-AAAGACATATCAGAGAGGCT
                                  D  S  S  W  N  E  I  Q  T   K  T  Y  Q  R  G
                   Change of splicing phase                frameshift

                     Q  Y  L  F  E  N  I  S  Q  L  T  E  K                intron
Human    PGLYRP4   CCAGTACCTATTTGAGAACATCTCCCAGCTCACTGAAAAAGGTAAACTGGATCTGGATAGT
Dugong   PGLYRP4   TCTGTATCTGTTTGGCAACGTTTCTAATCTCATTAAAAAAGGCAAACTGGCTCTGAATGGT
Manatee  PGLYRP4   TCTGTATCTGTTTGGCAACGTTTCTAATCTCATTAAAAAAGGCAAACTGGCTCTGAATGGT
                   F  C  I  C  L  A  T  F  L  I  S  L  K  K  A  N  W  L  -  M
                                                         Loss of splicing signal
```

Supplementary Figure S4. *PGLYRP4* is a pseudogene in sirenians. Nucleotide sequence alignment of homologous segments of exon 2 in human, dugong and manatee. The coding sequence of human and manatee *PGLYRP4* was translated and the amino acid sequence is shown above and below the nucleotide sequences, respectively. Intronic nucleotide sequences flanking the exons are marked by grey shading. Frame-shift mutations and mutations of splice sites in the sirenian genes are highlighted (red). Nucleotides conserved in all species are indicated by blue fonts. Species: Human (*Homo sapiens*), dugong (*Dugong dugon*), manatee (*Trichechus manatus latirostris*).

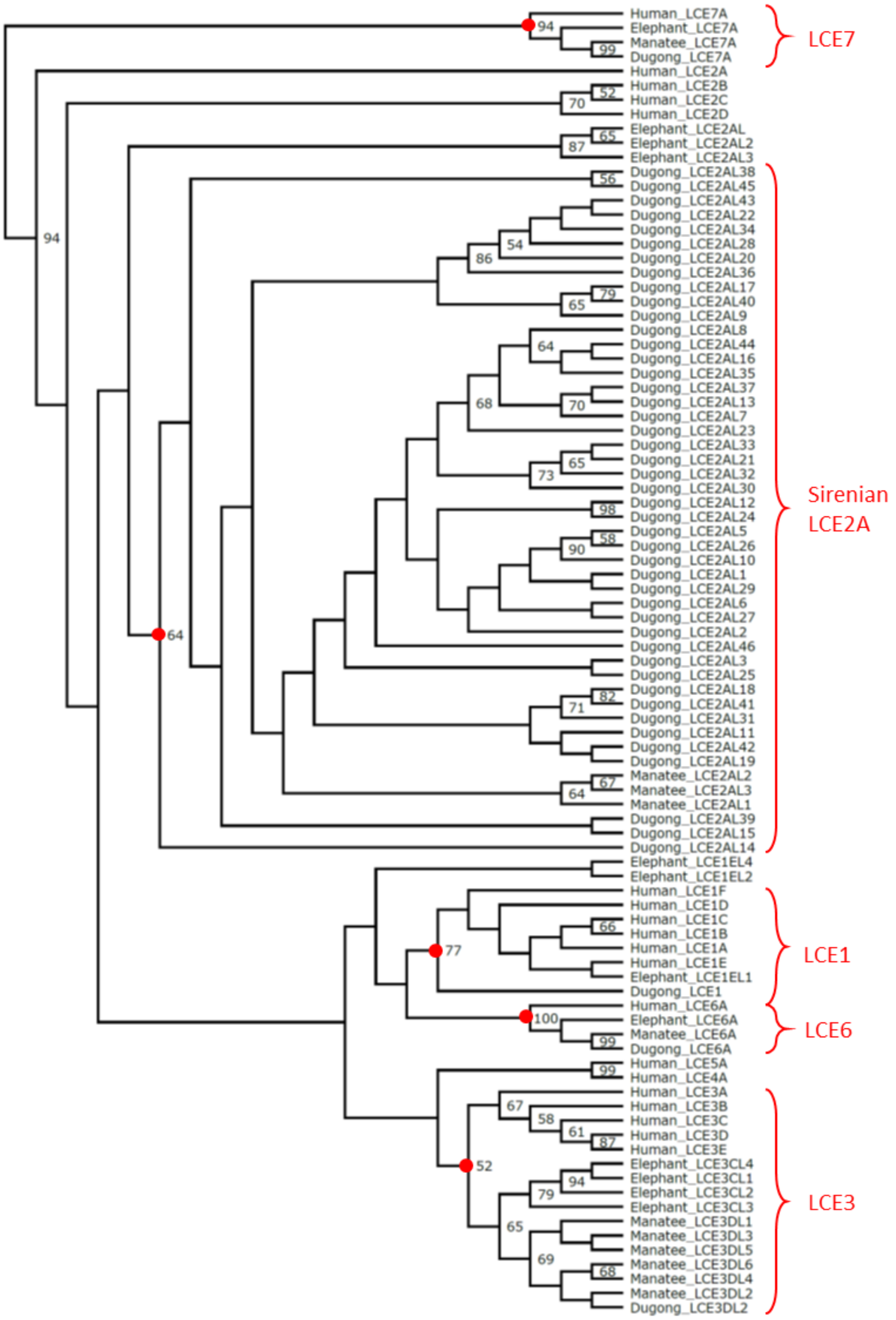

Supplementary Figure S5. Phylogeny of late cornified envelope (LCE) genes. Cladogram of human, elephant, manatee and dugong LCEs. The whole LCE sequence was used for the construction of the phylogenic analysis. A maximum likelihood tree was calculated as described in the Materials and methods section. Bootsrap values >50 are shown. Important monophyletic clades, including the clade of the sirenian LCE2A genes, are highlighted.

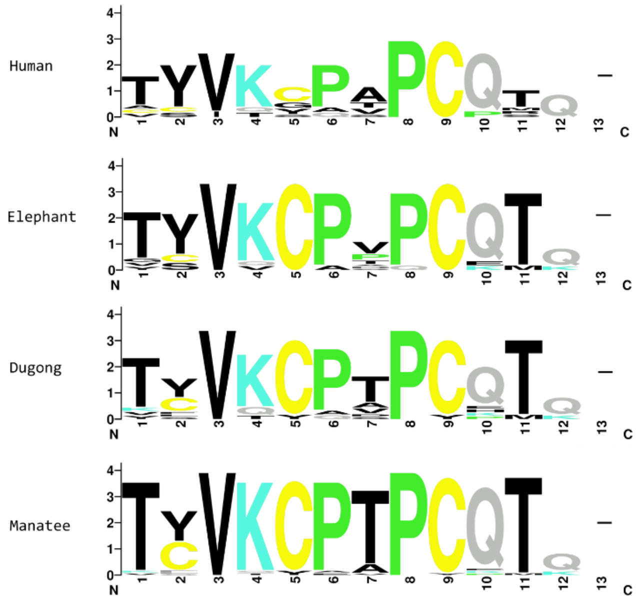

Supplementary Figure S6. Sequence repeats in the central region of KPLCE in human, elephant, dugong and manatee. Comparison of sequence repeat motifs of human, elephant, dugong and manatee KPLCEs. Amino acid residues are present in the one-letter code. The Weblogo software (Crooks et al. 2004) was used to generate sequence logos. Species: Human (*Homo sapiens*), elephant (*Elephas maximus indicus*), dugong (*Dugong dugon*), manatee (*Trichechus manatus latirostris*). The amino acid sequences of manatee and dugong KPLCE are shown in Supplementary Figures S1 and S2. GenBank accession numbers: Human *KPLCE*, NP_001019850.1; elephant *KPLCE*, XP_049731600.1.

A

```
           1                                                                                                  100
Hs_CRNN    MPQLLQNINGIIEAFRRYARTEGNCTALTRGELKRLLEQEFADVIVKPHDPATVDEVLRLLDEDHTGTVEFKEFLVLVFKVAQACFKTLSESAEGACGSQ
Em_CRNN    MPQLLRNIHGIIEAFGRYAKTEGNCMVLTRGELKRLLEHEFADVIVKPHDPVTVDEVLRLLDEDDTGTVEFKEFLVLVFKVAQACFKTLSESPEGVCGSQ
Tml_CRNN   MPQLLRNIHGIIEAFGRYAKTEGNCTVLTRGELKRLLEHEFADVIVKPHDPATVDEVLRLLDEDNTGTVEFKEFLVLVFKVAQACFKTLSESPEGACGSQ
Dd_CRNN    MPQLLRNIHGIIEAFGRYAKTEGDCTVLTRGELKRLLEHEFADVIVKPHDPVTVDEVLRLLDEDNTGTVEFKEFLVLVFKVAQACFKTLSESPEGACGSQ

           101                                                                                                200
Hs_CRNN    ESGSLHSGASQELGEGQRSGTEVGRAGKGQHYEGSSHRQSQQGSRGQNRPGVQTQGQATGSAWVSSYDRQAESQSQERISPQIQLSGQTEQTQKAGEGKR
Em_CRNN    ESRSCHSAASQELREGQRSHTEVGGAGRGQLHEGSSRGQSKQASTGQDG----------SSTQVTHHDRQAESQIQESMSQEAQETGQT---QSA-EDKS
Tml_CRNN   ESRSHHPGASQELREGQRSHTEVGWAGRGQLREGSSQGQSKQASLKHDG----------SSAQVSLHDKQVESQRQESPSQQAQETGHTEHTQTA-QDQR
Dd_CRNN    ESRSHHPGASQELREGQRSHTEVGWAGRGQLHEGSSRGQSKHAFLEHDG----------SSAQVSLHDKQVESQRQESPSQQAQETGHTEHTQTA-QDQR

           201                                                                                                300
Hs_CRNN    NQTTEMRPERQPQTREQDRAHQTGETVTGSGTQTQAGATQTVEQDSSHQTGRTSKQTQEATNDQNRGTETHGQGRSQTSQAVTGGHAQIQAGTHTQTPTQ
Em_CRNN    HQTREGQSERQSQTREQDRAHQTSETVTGTLTQTQTGATQTPGQDRSHQ---------------------------------------------------
Tml_CRNN   HQTRVGWSERQSQTREQSRAHQTSETVTGTLTQTQAGTTQTLGQDRSCQMGSTSTQSQESTCGQTGGTHS------------------------------
Dd_CRNN    HQTRVGWSERQFQTREQGRAHQTSETVTGTLTQTQAGTTQTLGQDRSHQMRSTSTQSQESTCGQTRGTHSQDRSHQMGSTSTQSQESTCGQMGGTHS---

           301                                                                                                400
Hs_CRNN    TVEQDSSHQTGSTSTQTQESTNGQNRGTEIHGQGRSQTSQAVTGGHTQIQAGS----HTETVEQDRSQTVSHGGAREQGQTQTQPGSGQR----------
Em_CRNN    ---------TGSTGSQARESNYSQTRGT--HSQDRSQTSQIVTGGHIQTQAGSQTQTTEQDRSHQTGNTSTQSWESTIGQTRGTHSQNRS----------
Tml_CRNN   ---QDRSRQTGSTSTQSQESTCGQTGGTETNGQDRNQTSQVVTG-HIQTQAGSQTQVHPQTVEQDRSQTGRHAGAREEGQSQTQSGSGQT----------
Dd_CRNN    ---QDRSHQTGSISTQSQESTYGQTRGT--HGQDKSQTSQVVIGGHVQTQVGSQTQTVEQDRSRQMGSTSTQSQESTCGQTRGTHSQDKSRQTGSTSTQS

           401                                                                                                500
Hs_CRNN    ----------------------------------------------------------------------------------------------------
Em_CRNN    -QTSQGVTGGY----VQTQTGS-QTQTVE-----------QDRSQQTGNTSTQSWVSSFHQTRETHDQDR-RQTSQTAT-----------GGHIQ-----
Tml_CRNN   ----------------------------------------------------------------------------------------------------
Dd_CRNN    QESTYGQTGGTHSQDKSRQTGSTSTQSQESTYGQTGGTHSQDRSRQTGSTSTQSQESTCGQTRGTHSQDKSRQTGSTSTQSQESTYGQTGGTHSQDKSRQ

           501                                                                                                600
Hs_CRNN    ----------------------------------------------------------------------------------------------------
Em_CRNN    ---TQAGSQTQTHTQAPG------------------------------QDESHQAGSTITQSRESNTGQTRGTRSQDRSQTSQGVTGGYVQTQTGSQTQT
Tml_CRNN   ----------------------------------------------------------------------------------------------------
Dd_CRNN    TGSTSTQSQESTYGQTGGTHSQDRSRQTGSTSTQSQESTCGQTRGTHSQDRSHQMGSTSTQSQESTCGQTRGTHSQDRSHQMGSTSTQSQESTCGQMGGT

           601                                                                                                700
Hs_CRNN    ----------------------------------------------------------------------------------------------------
Em_CRNN    GEQGRSQQTRNTSTQSRESTVGQIRGPHSQDRSQTSQTVTGGHVQTQAGSQTQTTEQDRSHQTGNTSTQSQESTIGQTRGTHSQNRS-----------QI
Tml_CRNN   ----------------------------------------------------------------------------------------------------
Dd_CRNN    HSQDRSHQTGSISTQSQESTYGQTRGTHGQDKSQTSQVVIGGHVQTQVGSQTQTVEQDRSRQMGSTSTQSQESTCGQTRGTHSQDKSRQTGSTSTQSQES

           701                                                                                                800
Hs_CRNN    ----------------------------------------------------------------------------------------------------
Em_CRNN    SQGVTGG---------------YVQTQTRSQTQAVE-QDRSQQTGNTSTQSRESTIGQTRGAHSQDRS-QTSQTVT-----------GGHVQTQA----G
Tml_CRNN   ----------------------------------------------------------------------------------------------------
Dd_CRNN    TYGQTGGTHSQDRSHQTGSTSTQSQESTYGQTRGTHSQDRSRQTGSTSTQSQESTYGQTGGTHSQDRSHQTGSTSTQSQESTYGQTGGTHSQDRSRQTGS

           801                                                                                                900
Hs_CRNN    ----------------------------------------------------------------------------------------------------
Em_CRNN    SQTQTTE-----------QDRSHQTGNTSTQSRESTIGQTRGTRSQNRS-----------QTSQGVTGGYVL---------TQTRSQTQTV-------EQ
Tml_CRNN   ----------------------------------------------------------------------------------------------------
Dd_CRNN    TSTQSQESTYGQTGGTHSQDRSRQTGSTSTQSQESTYGQTGGTHSQDRSRQTGSTSTQSQESTYGQTGGTHSQDRSRQTGSTSTQSQESTYGQTGGTHSQ

           901                                                                                               1000
Hs_CRNN    --------------------------------------------------------------------------------------WMQVSNPEAGETVP
Em_CRNN    DRSQQTGNTSTQSRVSTSGQTGATETNSQDGNQKSQVVTGGYIQTQAGSQTQVHPQTMEQDRSQMGSHVGAREQEQSQTQSGSGQTWTQVGNYEAGETVL
Tml_CRNN   --------------------------------------------------------------------------------------WTRVSNYEAGETVL
Dd_CRNN    DRSCQTGSTSTQSQESTYGQTRGTETNSQDRNQMSQVVTG-HIQTQAGSQTQVHPQAVEQDRSQTGRHAGAREEGQSQTQSGSGQTWTRVSNYEAGETVL

           1001                                                                                              1100
Hs_CRNN    GGQAQTGASTESGRQEWSSTHPRRCVTEGQGDRQPT-----VVGEEWVDDHSRETVILRLDQGNLHTSVSSAQGQDAAQSEEKRGITARELYSYLRSTKP
Em_CRNN    EGQAQPGASTLTGSQDWSSSHPSCSVTARQGEGEPHPKESNVVVEEWVDDPTREMVVQRQNQSSLHTSVSSAQGGETAQPE-GR----------------
Tml_CRNN   EGQPQPGAGTLPGSQDGSSMHPSYSVTGEQGESN-------VVVEEWVDDHTREMVIQRQDQGTLPSSASSALGGETAQPKERRGITAKGLYSYFQSTKP
Dd_CRNN    EGQPQPGAGTLPGSQDGSSMHPSYSVTGGQGESN-------VVVEEWVDDHTREMVIQRQDQGTLPSSASSALGGETAQPKERQGITAKGLYSYFQSTKP
```

B

```
           1                                                                                                  100
Hs_FLG     MSTLLENIFAIINLFKQYSKKDKNTDTLSKKELKELLEKEFRQILKNPDDPDMVDVFMDHLDIDHNKKIDFTEFLLMVFKLAQAYYESTRKENLPISGHK
Em_FLG     MSTLLENITAIIDLFQQYSSNDKENDTLSKKELEELLETEFQPILENPNDPDTADDFMHILDLDHNKKVDFTEFFLMVFKLAQAYYESTKRQNFKASGPK
Tml_FLG    MSILLENIVAIIDLFQQYSSKDKENDTLSEKELTELLEREFRSILKHPDDPDTTDVFMHFLDVDDNEKINFTEFLLMVFKLAQAYYESMKRKNFKATSTK
Dd_FLG     MSILLEKIVAIIDLFQQYSSKDKENDTLSKKELTELLEREFRSILKHPDDPDTTDVFMHVLDVDHNEKINFTEFLLMVFKLAQAYYESMKRKNFKATSTK

           101                                                                                                200
Hs_FLG     HRKHSHHDKHEDNKQEENK--ENRKRPSSLERRNNRKGNKGRSKSPRETGGKRHESSSEKKERKGYSPTHREEEYGKNHHNSSKKEKNKTENTRLGDNRK
Em_FLG     QKKYGHKCQNEEDEVEEEEKIEEQRRKSSLSRRHRQTGKKSLTTTDKK---KHRSSSKNKGRRDDSSPSGHREKNGKNHHHHTGKNK-RSSFTDLGEETN
Tml_FLG    QKKHGQERQNEEYEIEEEEKEEEQRRESILSSRHGRRGKRSPIRTDKK---KHGSISNSKRRRDSSSPFGHREKNERKHHHHTGKYYNRSSSTDLGEERN
Dd_FLG     QKTHGQECQNEDYEIEEEEKEEEQRRESILSSRHGRKGKRSPIRTDKK---KHGSISSSKGRRDSSSPFGHREKNERKHHHHTGKYYNRSSSTDLGEERD
```

```
         201                                                                                               300
Hs_FLG   RLSERLEEKEDNEEGVYDYENTGRMTQKWIQSGHIATYYTIQDEAYDTTDSLLEENKIYERSRSSDGKSSSQVNRSRHENTSQVPLQESRTRKRRGSRVS
Em_FLG   -ISSISQKREVGDEHNYGSENRERGSARW--------EYSNQEESED--------------SDRQAGPPGTNHNGFTHH--------QSRGRRHSGFNQE
Tml_FLG  TSSIGQKREQEGDEHDYGYEKRGRVSDTQ--------EDASQEESAH--------------SGRQSGSSSRNHHGSIHG--------QARDSSRHSRSLE
Dd_FLG   TSSIGQKREQEGDEHDYGYEKRGRVSDTH--------EDTSQEESAH--------------SGRQSGSSSRNHHGSTHG--------QARDSFRHSRSLE

         301                                                                                               400
Hs_FLG   QDRDSEGHSEDSERHSGSASRNHHGSAWEQSRDGSRHPRSHDEDRASHGHSADSSRQSGTRHAETSSRGQTASSHEQARSSPGERHGSGHQQSADSSRHS
Em_FLG   HRSDSQ-QLHSTHKHSHSAARGRQGSR--------------------HGHSVDSSTHSDSDQGEISSEGRS-------RSSTKSRQQSHQHNSADHPRHS
Tml_FLG  QQTADRAQSHSGHVHSDSTATGRQGSI--------------------HGQSVDSARHSGSHQGERSTGVQS-------RSSTRGRQEFHQDHSADPSRDS
Dd_FLG   QQTADHVQSHSGHVHSDSTATGRQGSV--------------------HGQSVHSARHSGSHQGQRSTGVQS-------RSTTRGRQEFHQDQSADRSRVS

         401                                                                                               500
Hs_FLG   ATGRGQASSAVSDRGHRGSSGSQASDSEGHSENSDTQSVSGHGKAGLRQQSHQESTRGRSGERSGRSGSSLYQVSTHEQPDSAHGRTGTSTGGRQGSHHE
Em_FLG   GTGRGRSTIESGAGSHRGSSVGQASNSEGQSEDSQRQSGSASGH-------HHGSNQGQSRDSSRHSGYHQGHRTTTPHSHSAHGHSDSTPIESQGPPYG
Tml_FLG  QSGCGKSSSEFRGDTHRGSSVSQTRDSEGQSEDSESQSGSSSRN-------HYGSTHGQATDSSRHSRSHEQQRSDHAQSGSAHGHSDSTAIGRQGATYG
Dd_FLG   QSGPGQSSSEFRTDTHRESSVSQASDSEGQSEDSERQSGSSSRN-------HYGSTHGQARDSSRHSRSHQDQRANRAWSGSAMDTQILLPQEEKDAFME

         501                                                                                               600
Hs_FLG   QARDSSRHSASQEGQDTIRGHPGSSRGGRQGSHHEQSVNRSGHSGSHHSHTTSQGRSDASHGQSGSRSASRQTRNEEQSGDGTRHSGSRHHEASSQADSS
Em_FLG   QSGDSSRHSGSHQGETSAHGQSRSSTRGRQKSRQEHSADRSRRSGPGHA----QSSVESGEGRHRGSSVSQASDSKGQSEDSHRQSGY----ASTHYHGS
Tml_FLG  QSVDSSRHSGSHQGERSAGVQSRSSARGRQDFHQDQSADRSTDSRSGRG----QSSSELGARTHRGSVVSQASDSGQQSEDSERQSAS----SSRNHHGF
Dd_FLG   RL--------------------------------------------------------------------------------------------------

         601                                                                                               700
Hs_FLG   RHSQVGQGQSSGPRTSRNQGSSVSQDSDSQGHSEDSERWSGSASRNHHGSAQEQSRDGSRHPRSHHEDRAGHGHSADSSRKSGTRHTQNSSSGQAASSHE
Em_FLG   THGQSRD-------TSRHSGSH-------QGHRADA---SQSHSAHGHSESTARGRQGS-----------SHGHSVDSSRHS-------SSHQGETSAHG
Tml_FLG  TQDLARD-------SFRHSRSL-------EQQTADR---AQSHCGHGYSDSTATGRQGS-----------IHGETVDNSRHSG-------SHQGERSTGV
Dd_FLG   ----------------------------------------------------------------------------------------------------

         701                                                                                               800
Hs_FLG   QARSSAGERHGSRHQLQSADSSRHSGTGHGQASSAVRDSGHRGSSGSQATDSEGHSEDSDTQSVSGHGQAGHHQQSHQESARDRSGERSRRSGSFLYQVS
Em_FLG   QPRSSTRGRQ-EYHQEHSADHARQSGTGHGRSPNESGAGRDRGSSVSQASDSEGQLENS-------HRQSGYASRHHQGSTHGQSRDYSGHSGSHQGHRA
Tml_FLG  QLRLTTRARQ-ELHQDQSADRSRDSRSGRGQSSSELGTSTHRGSSVSQARDSEGQSEDS-------ERQSGSSSRNHYGSTHSQARDSSRHSRSHQVQRA
Dd_FLG   ----------------------------------------------------------------------------------------------------

         801                                                                                               900
Hs_FLG   THKQSESSHGWTGPSTGVRQGSHHEQARDNSRHSASQDGQDTIRGHPGSSRRGRQGSHHEQSVDRSGHSGSHHSHTTSQGRSDASRGQSGSRSASRTTRN
Em_FLG   DASQSHSAHGHSDSTARGRQAYSHRQSVDSSRHSGSHQGETSAHGQSRSSTRGTQESPQEHSADRSTHSGAGHTHSSVESGEGRHRGSS----VSQATDS
Tml_FLG  DRAWSGSAHAHSDSTATGRKECIHGETVDTSRHSGSHQGEWSACVQLRSTTRARQELHQDQSADRSTDSRSGRAQSSSELGGRTHRGSV----VSQASDC
Dd_FLG   ----------------------------------------------------------------------------------------------------

         901                                                                                              1000
Hs_FLG   EEQSRDGSRHSGSRHHEASSHADISRHSQAGQGQSEGSRTSRRQGSSVSQDSDSEGHSEDSERWSGSASRNHRGSAQEQSRHGSRHPRSHHEDRAGHGHS
Em_FLG   EGQSEDSHRQSGY----ASTHYHGSTH----------------------------GQSRDTSRHSGSHQGHRADASQSHSAHGHSDSTVRGRQGSSHGHS
Tml_FLG  EGQSEDSERQPAS----SSRNHHGFTQ----------------------------DQARDSFRHSRSHEQQRVNGAQSHSGHGHSDSTATGRQGFIHGET
Dd_FLG   ----------------------------------------------------------------------------------------------------

         1001                                                                                             1100
Hs_FLG   ADSSRQSGTPHAETSSGGQAASSHEQARSSPGERHGSRHQQSADSSRHSGIPRRQASSAVRDSGHWGSSGSQASDSEGHSEESDTQSVSGHGQDGPHQQS
Em_FLG   VDSSRHSSFHQGETSACGQP-------RSSTRGRQESHQKHSGERSRDSGTGRGQSPNESGAGRHREFSVSQASDSEGQSDDSQTQS-------GYASRH
Tml_FLG  VDNSRHSGSHQGERSTGVQS-------RSTTRGRQEFHQDHSADPSRDSQSG--QSSSEFRADTHRGSSVSQASDSEGQSEDSERQS-------GSSSRN
Dd_FLG   ----------------------------------------------------------------------------------------------------

         1101                                                                                             1200
Hs_FLG   HQESARDWSGGRSGRSGSFIYQVSTHEQSESAHGRTRTSTGRRQGSHHEQARDSSRHSASQEGQDTIRAHPGSRRGGRQGSHHEQSVDRSGHSGSHHSHT
Em_FLG   HQGSTHGQSGDNSRNSRSHQGHRDDGPQSHSAHGHSDSTATGRQGSSHGQSVDSSRHLGSQQGETSTHRQSRSSTRGRQESHLEHSTDRSRHSGAGHAQS
Tml_FLG  HYGSTHSQARDSSRHSRSHQVQRADRAWSGSAHAHSDSTATGRKECIHGETVDTSRHSGSHQGERSACVQLRSTTRARQELHQDQSADPSTDSRSGRAQS
Dd_FLG   ----------------------------------------------------------------------------------------------------

         1201                                                                                             1300
Hs_FLG   TSQGRSDASHGQSGSRSASRQTRKDKQSGDGSRHSGSRHHEAASWADSSRHSQVGQEQSSGSRTSRHQGSSVSQDSDSERHSDDSERLSGSASRNHHGSS
Em_FLG   SFESRAVRHRAPSVSQASD----SEGQSEDSHRQSGY----ASTHYHGSTH---GQSRDSSRRSGSHQGHRADRPQSHPAHG--------------HSDS
Tml_FLG  SSELGGRTHRGSVVSQASD----CEGQSEDSERQPAS----SSRNHHGFTQ---DQARDSFRHSRSHEQQRVNGAQSHSGHG--------------HSDS
Dd_FLG   ----------------------------------------------------------------------------------------------------

         1301                                                                                             1400
Hs_FLG   REQSRDGSRHPGFHQEDRASHGHSADSSRQSGTHHTESSSHGQAVSSHEQARSSPGERHGSRHQQSADSSRHSGIGHRQASSAVRDSGHRGSSGSQVTNS
Em_FLG   TARGRQGS-----------SHGQSVDTSRHSGSHQRETSAHGQS-------RSSTRGRQESRQEHSADHSRHSGDGRGQSSIESGEGRHRGSSVSQASDS
Tml_FLG  TATGRQGS-----------IHGETVDNSRHSGSHQGERSTGVQS-------RSTTRGRQEFHQDHSADPSRDSQSG--QSSSEFRADTHRGSSVSQASDS
Dd_FLG   ----------------------------------------------------------------------------------------------------

         1401                                                                                             1500
Hs_FLG   EGHSEDSDTQSVSAHGQAGPHQQSHKESARGQSGESSGRSRSFLYQVSSHEQSESTHGQTAPSTGGRQGSRHEQARNSSRHSASQDGQDTIRGHPGSSRG
Em_FLG   EGQSEDSHRQSGSASGH-------HHGSNHGQSRDSSRHSRYHRGRRATAPNSHSAHGHSDSTAIGSQGPSYGQSVDSSRHSGSHQRETFTHGQSRSSTR
Tml_FLG  EGQSEDSERQSGSSSRN-------HYGSTHGQARDSSRHSRSHEQQRSDHAQSGSAHGHSDSTARGRQGATYGQSVDTSRHSGSHQGERSAGVQSRSSTR
Dd_FLG   ----------------------------------------------------------------------------------------------------

         1501                                                                                             1600
Hs_FLG   GRQGSYHEQSVDRSGHSGYHHSHTTPQGRSDASHGQSGPRSASRQTRNEEQSGDGSRHSGSRHHEPSTRAGSSRHSQVGQGESAGSKTSRRQGSSVSQDR
Em_FLG   GRQESRQKHSADHSRHSG-------------TGHGQSPNESGVR------------RHRGSSVIQASDSKGQSEDSQT----QSGYASRHHQGSTHGQSR
Tml_FLG  ARQELHQDQSADRSTDSR-------------SGRAQSPSELGGR------------THRGSVVSQASDCQGQSEDSER----QSASSSRNHYGFTQDQAR
Dd_FLG   ----------------------------------------------------------------------------------------------------
```

```
        1601                                                                                              1700
 Hs_FLG  DSEGHSEDSERRSESASRNHYGSAREQSRHGSRNPRSHQEDRASHGHSAESSRQSGTRHAETSSGGQAASSQEQARSSPGERHGSRHQQSADSSTDSGTG
 Em_FLG  DNSRHSGSHQEHRADGPESH-------SAHGHSDSTARGGQGSSHGQSVDSSRHSDSQQGKTSAHGQS-------RSSTRGRQESHLEHSADRSRHSGAG
Tml_FLG  DSFRHSRSHEQQRSDHAQSG-------SAHGHSDSTATRRKGSTRGQSVDSSRHSASHHGERSAGVQS-------RSSTRGRQELHQDQSADRSRDSWSG
 Dd_FLG  ----------------------------------------------------------------------------------------------------

        1701                                                                                              1800
 Hs_FLG  RRQDSSVVGDSGNRGSSGSQASDSEGHSEESDTQSVSAHGQAGPHQQSHQESTRGQSGERSGRSGSFLYQVSTHEQSESAHGRTGPSTGGRQRSRHEQAR
 Em_FLG  HAQSSFESRAVRHRGPSVSRASDREGRSEDS-------HRQSGSASTHYHGSTHGQSRDSSRHSGSHQGHRANGPQSHPARGHSDSTARGRQGSSHGQSV
Tml_FLG  LGQSSSEFRASTHRGSSVIQASDNEGQSEESARQSASNYETSGSSSRKQAESTHGQSGEHQRRSGAHQGQKSTHGESDSTQEHQRQKADHGQADSN----
 Dd_FLG  ----------------------------------------------------------------------------------------------------

        1801                                                                                              1900
 Hs_FLG  DSSRHSASQEGQDTIRGHPGSSRGGRQGSHYEQSVDSSGHSGSHHSHTTSQERSDVSRGQSGSRSVSRQTRNEKQSGDGSRHSGSRHHEASSRADSSRHS
 Em_FLG  DTSRHSGSHQRETSAHGQSRSSTRGRQESRQEHSADRSRHSGAGYAQSSLESGAVRHRGSS----VSQASDSEGQSEDSHRQSGS----ASGHHHGSNH-
Tml_FLG  DTKSEGSSLRFHS---------------------------------------------------------------------------------------
 Dd_FLG  ----------------------------------------------------------------------------------------------------

        1901                                                                                              2000
 Hs_FLG  QVGQGQSSGPRTSRNQGSSVSQDSDSQGHSEDSERWSGSASRNHLGSAWEQSRDGSRHPGSHHEDRAGHGHSADSSRQSGTRHTESSSRGQAASSHEQAR
 Em_FLG  --GQSRDSSRHSGYHRGHRATALNSHSTHGHSDSTAIGSQ-----GPSYGQSVDSSRHSGSHQRETFTHGQS------------RSSTRGRQESRQEH--
Tml_FLG  ----------------------------------------------------------------------------------------------------
 Dd_FLG  ----------------------------------------------------------------------------------------------------

        2001                                                                                              2100
 Hs_FLG  SSAGERHGSHHQLQSADSSRHSGIGHGQASSAVRDSGHRGYSGSQASDSEGHSEDSDTQSVSAQGKAGPHQQSHKESARGQSGESSGRSGSFLYQVSTHE
 Em_FLG  --------------SADRSRHSGDGHGQSSAESGEGRHRGSSVIQASYSEGQSEDSHRQS----GYASTHYHG---STHGQARDSSRHSGSHQGHRADAS
Tml_FLG  ----------------------------------------------------------------------------------------------------
 Dd_FLG  ----------------------------------------------------------------------------------------------------

        2101                                                                                              2200
 Hs_FLG  QSESTHGQSAPSTGGRQGSHYDQAQDSSRHSASQEGQDTIRGHPGPSRGGRQGSHQEQSVDRSGHSGSHHSHTTSQGRSDASRGQSGSRSASRKTYDKEQ
 Em_FLG  QSHSAHGHSDSTATGRQGSSHGQSVDSSRHSDSQQGETSAHGQSRSSTRGRQESHLEHSTDRSRHSGAGHAQSSFESRAVRHRAPSVSQ-ASDSEGQSED
Tml_FLG  ----------------------------------------------------------------------------------------------------
 Dd_FLG  ----------------------------------------------------------------------------------------------------

        2201                                                                                              2300
 Hs_FLG  SGDGSRHSGSHHHEASSWADSSRHSLVGQGQSSGPRTSRPRGSSVSQDSDSEGHSEDSERRSGSASRNHHGSAQEQSRDGSRHPRSHHEDRAGHGHSAES
 Em_FLG  SHRQSGYASTHYH--GSTHGQSRDSSRRSGSHQGHRADRP------QSHPAHGHSDSTAR----------------ARQGS-----------SHGQPEDS
Tml_FLG  ----------------------------------------------------------------------------------------------------
 Dd_FLG  ----------------------------------------------------------------------------------------------------

        2301                                                                                              2400
 Hs_FLG  SRQSGTHHAENSSGGQAASSHEQARSSAGERHGSHHQQSADSSRHSGIGHGQASSAVRDSGHRGSSGSQASDSEGHSEDSDTQSVSAHGQAGPHQQSHQE
 Em_FLG  SRHSGSHQGETSARGEY-------RSSTRDRQESLQEHSADHSRHSGAGNAQSSFESGAGRHRGSSVSQASDSEGQSEDS-------HRQSGFASTQYHG
Tml_FLG  ----------------------------------------------------------------------------------------------------
 Dd_FLG  ----------------------------------------------------------------------------------------------------

        2401                                                                                              2500
 Hs_FLG  STRGRSAGRSGRSGSFLYQVSTHEQSESAHGRTGTSTGGRQGSHHKQARDSSRHSTSQEGQDTIHGHPGSSSGGRQGSHYEQLVDRSGHSGSHHSHTTSQ
 Em_FLG  STHGQSRDSSKHLGSHQGHRANGPQSHSVHGHSDSTARGRKGSSHGQSVDSSRHSGSHQGETSAHGQSRSSTRGRQETQQEHSADHSRHSVSGHG-----
Tml_FLG  ----------------------------------------------------------------------------------------------------
 Dd_FLG  ----------------------------------------------------------------------------------------------------

        2501                                                                                              2600
 Hs_FLG  GRSDASHGHSGSRSASRQTRNDEQSGDGSRHSGSRHHEASSRADSSGHSQVGQGQSEGPRTSRNWGSSFSQDSDSQGHSEDSERWSGSASRNHHGSAQEQ
 Em_FLG  -------------------RSSFESGAG-RHRGSSVGQASDSKGQSEHSGIQSGSGSG-----------QQGRSTYSQSRDSSRHSGSPQGLRADAPQSH
Tml_FLG  ----------------------------------------------------------------------------------------------------
 Dd_FLG  ----------------------------------------------------------------------------------------------------

        2601                                                                                              2700
 Hs_FLG  LRDGSRHPRSHQEDRAGHGHSADSSRQSGTRHTQTSSGGQAASSHEQARSSAGERHGSHHQQSADSSRHSGIGHGQASSAVRDSGHRGYSGSQASDNEGH
 Em_FLG  TAHGHSYSSAIGKQGSSQGQSLDSTRHSGSHHGETSA-------HGESRSSTRGKKESRQEHSADTSRQSGTGRGRSTIESGAGRHGGSSVSQASDSEGQ
Tml_FLG  ----------------------------------------------------------------------------------------------------
 Dd_FLG  ----------------------------------------------------------------------------------------------------

        2701                                                                                              2800
 Hs_FLG  SEDSDTQSVSAHGQAGSHQQSHQESARGRSGETSGHSGSFLYQVSTHEQSESSHGWTGPSTRGRQGSRHEQAQDSSRHSASQDGQDTIRGHPGSSRGGRQ
 Em_FLG  SEDSHRQSGSASGH-------HLEFNHGQSRDSSRHSGYHQGHRATVPQSHSAHGHSDSTAIESQGPSYGQSGDSSRHSGSHQGETSAHGQSRSRTRGRQ
Tml_FLG  ----------------------------------------------------------------------------------------------------
 Dd_FLG  ----------------------------------------------------------------------------------------------------

        2801                                                                                              2900
 Hs_FLG  GYHHEHSVDSSGHSGSHHSHTTSQGRSDASRGQSGSRSASRTTRNEEQSGDGSRHSGSRHHEASTHADISRHSQAVQGQSEGSRRSRRQGSSVSQDSDSE
 Em_FLG  ESRQEHSADRSRHSGAGHAQSSLESGAVRHRGSSVSQASD----SEGQSEDSHRLSGY----ASTHYHGSTHGQA-RDSSRHSGSHQGHRADASQSHSAH
Tml_FLG  ----------------------------------------------------------------------------------------------------
 Dd_FLG  ----------------------------------------------------------------------------------------------------

        2901                                                                                              3000
 Hs_FLG  GHSEDSERWSGSASRNHHGSAQEQLRDGSRHPRSHQEDRAGHGHSADSSRQSGTRHTQTSSGGQAASSHEQARSSAGERHGSHHQQSADSSRHSGIGHGQ
 Em_FLG  GHSE-------STARGRQGSS--------------------HGYSVDSSRHSSSHQGETSA-------HGQPRSSTGGRQKSHQEHSADHSRQSGTGHGR
Tml_FLG  ----------------------------------------------------------------------------------------------------
 Dd_FLG  ----------------------------------------------------------------------------------------------------
```

```
         3001                                                                                             3100
 Hs_FLG  ASSAVRDSGHRGYSGSQASDNEGHSEDSDTQSVSAHGQAGSHQQSHQESARGRSGETSGHSGSFLYQVSTHEQSESSHGWTGPSTRGRQGSRHEQAQDSS
 Em_FLG  SPNESGAGRDRGSSVSQASDSEGQSEDS-------HRQSGYVSRHHQGSTHGQSRDYSRHSGSHQGHRDDGPHSHSAHGHSDSTARGRQGSSHGQSVDSS
Tml_FLG  ----------------------------------------------------------------------------------------------------
 Dd_FLG  ----------------------------------------------------------------------------------------------------

         3101                                                                                             3200
 Hs_FLG  RHSASQYGQDTIRGHPGSSRGGRQGYHHEHSVDSSGHSGSHHSHTTSQGRSDASRGQSGSRSASRTTRNEEQSGDSSRHSVSRHHEASTHADISRHSQAV
 Em_FLG  RHSDSHQGESSAHGQSRSSTKGRQESHQERSADRSRHSGAGHAQSSFESRAVRHRGPSVSRASDREGRSEDSHRQSG--SASTHYHGSTH----------
Tml_FLG  ----------------------------------------------------------------------------------------------------
 Dd_FLG  ----------------------------------------------------------------------------------------------------

         3201                                                                                             3300
 Hs_FLG  QGQSEGSRRSRRQGSSVSQDSDSEGHSEDSERWSGSASRNHRGSVQEQSRHGSRHPRSHHEDRAGHGHSADRSRQSGTRHAETSSGGQAASSHEQARSSP
 Em_FLG  ------------------------GQSRDSSRHSGSHQGHRADGPQSHSAHGHSDSTARGRQGLSHGQSADSSRHSDSHQGETST-------HGQSRSST
Tml_FLG  ----------------------------------------------------------------------------------------------------
 Dd_FLG  ----------------------------------------------------------------------------------------------------

         3301                                                                                             3400
 Hs_FLG  GERHGSRHQQSADSSRHSGIPRGQASSAVRDSRHWGSSGSQASDSEGHSEESDTQSVSGHGQAGPHQQSHQESARDRSGGRSGRSGSFLYQVSTHEQSES
 Em_FLG  RGRQESRQEHSADRSRHSGAGYAQSSLESGAVRHRGSSVSQASDSEGQSEDSHRQS--GYASTHYHGSTHGQS-RDSSR----HSGSHQGHRADASQSHS
Tml_FLG  ----------------------------------------------------------------------------------------------------
 Dd_FLG  ----------------------------------------------------------------------------------------------------

         3401                                                                                             3500
 Hs_FLG  AHGRTRTSTGRRQGSHHEQARDSSRHSASQEGQDTIRGHPGSSRRGRQGSHYEQSVDRSGHSGSHHSHTTSQGRSDASRGQSGSRSASRQTRNDEQSGDG
 Em_FLG  AHGHSESTARGRQGSSHGYSVDSSRHSSSHQGETSAHGQPRSSTGGRQKSHQEHSADHSRQSGTGHGRSPNESGAGRDRGSSVSQASDSEGQSEDSHRQS
Tml_FLG  ----------------------------------------------------------------------------------------------------
 Dd_FLG  ----------------------------------------------------------------------------------------------------

         3501                                                                                             3600
 Hs_FLG  SRHSWSHHHEASTQADSSRHSQSGQGQSAGPRTSRNQGSSVSQDSDSQGHSEDSERWSGSASRNHRGSAQEQSRDGSRHPTSHHEDRAGHGHSAESSRQS
 Em_FLG  GYAS--RHHQGSTHGQSRDYS-------------RHPGSHQGHRDDGP-HSHSAHGHSDSTARGRQGSSHGQSVDSSRHSDSHQGESSAHG---------
Tml_FLG  ----------------------------------------------------------------------------------------------------
 Dd_FLG  ----------------------------------------------------------------------------------------------------

         3601                                                                                             3700
 Hs_FLG  GTHHAENSSGGQAASSHEQARSSAGERHGSHHQQSADSSRHSGIGHGQASSAVRDSGHRGSSGSQASDSEGHSEDSDTQSVSAHGQAGPHQQSHQESTRG
 Em_FLG  ------------------QSRSSTRGRQESHQERSAERSRHSGAGHAQSSLESGAVRHRGSSVSQASDSKGQSEDSHRQS----GYASGHQL---ESNHG
Tml_FLG  ----------------------------------------------------------------------------------------------------
 Dd_FLG  ----------------------------------------------------------------------------------------------------

         3701                                                                                             3800
 Hs_FLG  RSAGRSGRSGSFLYQVSTHEQSESAHGRAGPSTGGRQGSRHEQARDSSRHSASQEGQDTIRGHPGSRRGGRQGSYHEQSVDRSGHSGSHHSHTTSQGRSD
 Em_FLG  QSRDSSRHSGYHRGHKATAPNSHSVHGHSDSTAKGSQGPSYRQSVDSSRHSASHQGETSTHGQSRSSTRGRQESHQEHSADRSRHSGAGHARSSIESGAG
Tml_FLG  ----------------------------------------------------------------------------------------------------
 Dd_FLG  ----------------------------------------------------------------------------------------------------

         3801                                                                                             3900
 Hs_FLG  ASHGQSGSRSASRETRNEEQSGDGSRHSGSRHHEASTQADSSRHSQSGQGESAGSRRSRRQGSSVSQDSDSEAYPEDSERRSESASRNHHGSSREQSRDG
 Em_FLG  RLRGSIVSQASGTEGQSEVSFGQSA----------------SNHESSGFG-----------------------------------SRNQPGSIHGQSGDS
Tml_FLG  ----------------------------------------------------------------------------------------------------
 Dd_FLG  ----------------------------------------------------------------------------------------------------

         3901                                                                                             4000
 Hs_FLG  SRHPGSSHRDTASHVQSSPVQSDSSTAKEHGHFSSLSQDSAYHSGIQSRGSPHSSSSYHYQSEGTERQKGQSGLVWRHGSYGSADYDYGESGFRHSQHGS
 Em_FLG  LRHSGSHSGQISTQRQSDSTHVHQSQKADHGQSDS--------NGRINQGS----SLCQFYSVNNDRQRHDAGHCWRHGSYGSSDYDYGQSRFGQS----
Tml_FLG  --------------------------------------------------------------VNNDRRSHGAGQCWRHGSYGSTDYDYGQSGFGQSQYGI
 Dd_FLG  ----------------------------------------------------------------------------------------------------

         4001                                                          4063
 Hs_FLG  VSYNSNPVVFKERSDICKASAFGKDHPRYYATYINKDPGLCGHSSDISKQLGFSQSQRYYYYE
 Em_FLG  ---------------------------------------------------------------
Tml_FLG  VRNDRSGTKQVSSIYEILYARQ-----------------------------------------
 Dd_FLG  ---------------------------------------------------------------
```

C

```
         1                                                                                                 100
 Hs_FLG2  MTDLLRSVVTVIDVFYKYTKQDGECGTLSKGELKELLEKELHPVLKNPDDPDTVDVIMHMLDRDHDRRLDFTEFLLMIFKLTMACNKVLSKEYCKASGSK
 Em_FLG2  MTDLLRSVVTIIDIFYKYTRQDGECGTLSKEELKELLEKEFHPILKNPDDPDTVDVIMHMLDRDHDRRLDFTEFLLMVFKLAMACNKVLSKEYCQASGSK
Tml_FLG2  MTDLLRSVVTVIDIFHKYTKQDGECATLSKDELKELLEKEFHPILKNPDDTDTVDVNMHMLDQDHDRQLDFTEFLLMVFKLAMACNKALSKEYCKASGSK
 Dd_FLG2  MTDLLRSVVTIIDIFYKYTKQDGECATLSKDELKELLEKEFHPILKNPDDADTVDVIMHMLHQDHDRXLDFTEFLLMVFKLAMACNKALSKEYCKASGSK

         101                                                                                               200
 Hs_FLG2  KHRRGHRHQEEESETEEDEEDTPGHKSGYRHSSWSEGEEHGYSSGHSRGTVKCRHGSNSRRLGRQGNLSSSGNQEGSQKRYHRSSCGHSWSGGKDRHGSS
 Em_FLG2  KHRHGHQHQEEESETEEEEEETLGQKSGHRYSSWSEREEHGYGSGGSSGTVKHRRGSKSRRLGRRDGLSSSENEEGSEKRSHGSHSGHFWSSGKERHGSR
Tml_FLG2  KCRHGHQHQEEESETEEEEEETQGWKSGYRYSSWNEREEHQYGSAGSRGTMKHRLRSNSRRLGRQGGLSSSENEEGSEKMCHGSSSGHSWSSGKERHGSS
 Dd_FLG2  KCRHGHQHQEEESETEEEEEETQGWKSGYRYSSWNEIEEHQYGSAGSRGTMKHRLRSNSRRLGRQGGLSSSENEEGSEKRHHGSSSGHLWSSGKERHGSS

         201                                                                                               300
 Hs_FLG2  SVELRERINKSHISPSRESGEEYESGSGSNSWERKGHGGLSCGLETSGHESNSTQSRIREQKLGSSCSGSGDSGRRSHACGYSNSSGCGRPQNASSSCQS
 Em_FLG2  SEELGERRNKSSVSPSRESGEEYEYGSGSKSRGRKAHGGLSHGLEASGHESSSTQSRSGGQSLRSSSGGSGDCGRQSHTCNSSNSEGYGRSQNASSSCQA
Tml_FLG2  SEELGERRNKLSVSSCGESGEEYECGSGSKNGGRKGHGGLSLELEASGHESSSIQSRSGGQRLGSIFGGSGDCGRQSHACNSSNSGGCGSPQNASSSCQA
 Dd_FLG2  SEELGERRNKLSVSSCGESGEEYECGSGSKSGGRKGHGGLSLELEASGHESSSIQSRSRGPRLGSIFGGSGDCGRQSHACNSSSSGGCGSPQNASSSCQA
```

```
          301                                                                                              400
 Hs_FLG2  HRFGGQGNQFSYIQSGCQSGIKGGQGHGCVSGGQPSGC-----GQPESNPCSQSYSQRGYGARENGQPQNCGGQWR-TGSSQSSCCGQYGSGGSQSCSNG
 Em_FLG2  GRFGGLGNQSSCTQSGYESGSSEGQGHEGISGGQSSGCQFSGYSQHNSGSSSQSSSHRGHESRARGQPQNSRGQQQGTGSSQSSCYEQYGSGVSQSSSYG
Tml_FLG2  GRFGGQGNQSSCTQSGYQSGSSGGQGHGCISGGQSSG-----YSQHNSGSYSQCSSHRGHGSRACGQPQNCRGQQQGTGSSQSSCCGQYGSGASQSSSYG
 Dd_FLG2  GRFGGQGNQSSCTQSGYQSGSSGGQGHGCISGGQSSG-----YSQHNSGSCSQSSSHRGHGSRACGQPQNCRGQQQGTGSSQSSCCEQYGSGASQSSSYG

          401                                                                                              500
 Hs_FLG2  QHEYGSCGRFSNSSSSNEFSKCDQYGSGSSQSTSFEQHGTGLSQSSGFEQHVCGSGQTCGQHESTSSQSLGYDQHGSSSGKTSGFGQHGSGSGQSSGFGQ
 Em_FLG2  QYEYGSCGSSSTSSQKR------------SGSNEFSKRG----------QLDPGSGHFSSQHRSSSSQSSG--------------QHGSGPSQSS-YGQ
Tml_FLG2  QQGYGSCGHSFTSSQKR------------SGSNMFSKCG----------QCGSGSGQSSSQHRSSSSQSSG------------FGQHGSGTGQSS--GQ
 Dd_FLG2  QQGYGSCGHSFTSSQKR------------SGSNVFSKCG----------QCGSGSGQSSSPHRSSSSQSSS------------FGQHGSGTGQSS--GQ

          501                                                                                              600
 Hs_FLG2  CGSGSGQSSGFGQHGSVSGQSSGFGQHGSVSGQSSGFGQHESRSRQSSYGQHGSGSSQSSGYGQYGSRETSGFGQHGLGSGQSTGFGQYGSGSGQSSGFG
 Em_FLG2  HGSGSHQSSG--QHGSFTGQSSS--QHRSSSSQSSS--QHGSGTAQFS-GQHGSGSSQSS------------FGQHGSGSGQSSGFGQHGSGLSQSS-YG
Tml_FLG2  HGSGSSQFSG--QHGSGTGQSSG--QHGSGSSQFS--------------GQHGSGTG-------------------------------------------
 Dd_FLG2  HGSGSSQSSG--QHGSGSSQSSGFGQHELGLHQSS----HS--------GQHSSGSGQSSS-----------FEPHGSSSGKSSSTSQHGSSVSRKGSCG

          601                                                                                              700
 Hs_FLG2  QHGSGSGQSSGFGQHESRSGQS-SYGQHSSGSSQSSGYGQHGSRQTSGFGQHGSGSSQSTGFGQYGSGSGQSSGFGQHVSGSGQSSGFGQHESRSGHSSY
 Em_FLG2  QHGTSSSQSSG--QLGSCTGQS-S-SQHRSSSSQSSG--QHGSGTGQFSGQQGSGSSQSS-FGQHGSGSGQSSG--QHVSGSSQSS-------------Y
Tml_FLG2  ----------------------------------------------------------------------------------------------------
 Dd_FLG2  ATSGKNYPKSGRAISDSSHSQTRGSSQERTTQSDSEKCSGHSISRSESKGHYSIHGDRQQRQGSSEEGRALGRTGRQETRSSQSSDSEGQSHNRERYPAY

          701                                                                                              800
 Hs_FLG2  GQHGFGSS-QSSGYGQHGSSSGQTSGFGQHELSSGQSSSFGQHGSGSGQSSGFGQHGSGSGQSSGFGQHESRSGQSSYGQHSSGSSQSSGYGQHGSRQTS
 Em_FLG2  SHHGSGSS-QSSG--QHLSGSSQ--------------SSYGQHGSGSSQSSG--QHGSCTGQSSS--------------RHRSSSSQSSG----------
Tml_FLG2  ----------------------------------------------------------------------------------------------------
 Dd_FLG2  SQRRFRKDHEESGVLSHEQSE----------------SCKRRQVSSHRQSSDSERHTGETNTHSGC-------------THGHSESQHG-----------

          801                                                                                              900
 Hs_FLG2  GFGQHGSGSSQSTGFGQYGSGSGQSAGFGQHGSGSGQSSGFGQHESRSHQSSYGQHGSGSSQSSG--YGQHGSSSGQTSGFGQHRSSSGQYSGFGQHG-S
 Em_FLG2  ---QHGSGSSQSSGFGQNGSSSSQSSG--QHGYGIGQSS-YGQHGSQSSQFSE-QHGSGSGQFSG--FGQHGSGSGQSSGFKQYESRSSQSSG--QHR-S
Tml_FLG2  ----------------------------------------------------------------------------------------------------
 Dd_FLG2  ---ELGFSRRQGSSHGQLGNTTRHTDSGPGKAIKTESRTIRRRGSSHGESSDSETHSGVSQRHSGSTHGQSGSPHGESETSRRQGSSHGQSGDTTRHAQT

          901                                                                                             1000
 Hs_FLG2  GSGQSSGFGQHGTGSGQYSGFGQHESRSHQSSYGQHGSGSSQSSGYGQH-GSSSGQTFGFGQHRSGSGQSSGFGQHGSGSGQSSGFGQHESGSGKSSGFG
 Em_FLG2  GSCQSSR--QQGYGSSQ-SSYGQHGCRSSQSS-GQHGPGISQSS-YGQH-RSSSSQSSG--QHGYGSSQSSGFSQHGSGSGQTSGFGQHELGSRQSS---
Tml_FLG2  ----------------------------------------------------------------------------------------------------
 Dd_FLG2  GHGQSTQSGSMTTGRRD-SSHSQSSDSKQHSGVSQRHSGSTQGQSGSQHVESGSGEDRDLLMDNPETSQDMPRPARDNPQGQ--GPGQPEEGILATVSPV

          1001                                                                                            1100
 Hs_FLG2  QHESRSSQSNYGQHGSGSSQSSGYGQHGSSSGQTTGFGQHRSSSGQYSGFGQHGSGSDQSSGFGQHGTGSGQSSGFGQYESRSRQSSYGQHGSGSSQSSG
 Em_FLG2  ---------HSGQHSSGSGQSPSFEPQGSSSGKSSSSGQHRSS---ISGKGSCGPIS------GKNYPKSGRTISDSSHSQARSSSQERTKQGDSERSQG
Tml_FLG2  ----------------------------------------------------------------------------------------------------
 Dd_FLG2  TVNSIQETPEDTQHLLMDNLSLPSNIQ-------------------------------------------------------------------------

          1101                                                                                            1200
 Hs_FLG2  YGQHGSNSGQTSGFGQHRPGSGQSSGFGQYGSGSGQSSGFGQHGSGTGKSSGFAQHEYRSGQSSYGQHGTGSSQSSGCGQHESGSGPTTSFGQHVSGSDN
 Em_FLG2  HSISGSESEDHDSTHQHRQQRQGSNEEGRVPGR--QETRYSQTSDSEGQSDNWERYSASPHTSSRIDHEVPSHEKSGSSRRQQ-----------------
Tml_FLG2  ----------------------------------------------------------------------------------------------------
 Dd_FLG2  ----------------------------------------------------------------------------------------------------

          1201                                                                                            1300
 Hs_FLG2  FSSSGQHISDSGQSTGFGQYGSGSGQSTGLGQGESQQVESGSTVHGRQETTHGQTINTTRHSQSGQGQSTQTGSRVTRRRRSSQSENSDSEVHSKVSHRH
 Em_FLG2  --SSQRQSSDSEQHSGSSQRHSGSTH----GQSGSQHGESGSS--RRKGSSHGHSGDTTRHDQSGHGQLTQSGSRTTERRDSSHSQSSDSEWQSEDSQRH
Tml_FLG2  ----------------------------------------------------------------------------------------------------
 Dd_FLG2  ----------------------------------------------------------------------------------------------------

          1301                                                                                            1400
 Hs_FLG2  SEHIHTQAGSHYPKSGSTVRRRQGTTHGQRGDTTRHGHSGHGQSTQTGSRTSGRQRFSHSDATDSEVHSGVSHRPHSQEQTHSQAGSQHGESESTVHERH
 Em_FLG2  SGSTHGQSGSQQGESGSS--RRQGTSHEQSGDITRHASSGHGQLTESVSGTTGRRGSSHSQSSDSEQHSGDSQR-HS-ESTHGQSGSQHGESGSS--RRQ
Tml_FLG2  ----------------------------------------------------------------------------------------------------
 Dd_FLG2  ----------------------------------------------------------------------------------------------------

          1401                                                                                            1500
 Hs_FLG2  ETTYGQTGEATGHGHSGHGQSTQRGSRTTGRRGSGHSESSDSEVHSGGSHRPQSQEQTHGQAGSQHGESGSTVHGRHGTTHGQTGDTTRHAHYHHGKSTQ
 Em_FLG2  GSSHGHSGETTRHDQSGHEQLTQSGSRTTERRDSSHSQSSDSEWQSEDSQRHSGS--THGQSGSQQGESGSS--RRQGTSHEQSGDITRHASSGHGQLTE
Tml_FLG2  ----------------------------------------------------------------------------------------------------
 Dd_FLG2  ----------------------------------------------------------------------------------------------------

          1501                                                                                            1600
 Hs_FLG2  RGSSTTGRRGSGHSE-SSDSEVHSGGSHTHSGHTHGQSGSQHGESESIIHDRHRITHGQTGDTTRHSYSGHEQTTQTGSRTTGRQRTSHSESTDSEVHSG
 Em_FLG2  SGSGTTGRRGSSHSQQSSDSEQHSGDSQRHSESTHGRSRSQHGESGS--SRRQGSSHGQSGETTRHANSGHGQLTQSGSRTTGRRDSSHSQSSDIEQHSG
Tml_FLG2  ----------------------------------------------------------------------------------------------------
 Dd_FLG2  ----------------------------------------------------------------------------------------------------

          1601                                                                                            1700
 Hs_FLG2  GSHRPHSREHTYGQAGSQHEEPEFTVHERHGTTHGQIGDTTGHSHSGHGQSTQRGSRTTGRQRSSHSESSDSEVHSGVSHTHTGHTHGQAGSQHGQSESI
 Em_FLG2  NSYR-HSRSG-HDQSGSQHGESGPS--RRQENFHGQSGDTTKQAQSSHGQRTESGSRTAERRDSSHSQSNDSEWHSGVSERHTASTYGQSGSSSKQSVSN
Tml_FLG2  ----------------------------------------------------------------------------------------------------
 Dd_FLG2  ----------------------------------------------------------------------------------------------------
```

```
          1701                                                                                          1800
  Hs_FLG2 VPERHGTTHGQTGDTTRHAHYHHGLTT---QTGSRTTGRRGSGHSEYSDSEGYSGVSHTHSGHTHGQARSQHGESESIVHERHGTIHGQTGDTTRHAHSG
  Em_FLG2 STRTYESSNEHSEDIPKHSVSHQRQTSPYGQPGYRTSGRQGSPHVQRGDRSTHSGSIHGQSG-SRTTGRQGSTQSHDIHRQSRDSIRTQSSSSHHQPGAN
 Tml_FLG2 ----------------------------------------------------------------------------------------------------
  Dd_FLG2 ----------------------------------------------------------------------------------------------------

          1801                                                                                          1900
  Hs_FLG2 HGQSTQTGSRTTGRRSSGHSEYSDSEGHSGFSQRPHSRGHTHGQAGSQHGESESIVDERHGTTHGQTGDTSGHSQSGHGQSTQSGSSTTGRRRSGHSESS
  Em_FLG2 H---------------------------------------------------------------------------------------------------
 Tml_FLG2 ----------------------------------------------------------------------------------------------------
  Dd_FLG2 ----------------------------------------------------------------------------------------------------

          1901                                                                                          2000
  Hs_FLG2 DSEVHSGGSHTHSGHTHSQARSQHGESESTVHKRHQTTHGQTGDTTEHGHPSHGQTIQTGSRTTGRRGSGHSEYSDSEGPSGVSHTHSGHTHGQAGSHYP
  Em_FLG2 ----------------------------------------------------------------------------------------------------
 Tml_FLG2 ----------------------------------------------------------------------------------------------------
  Dd_FLG2 ----------------------------------------------------------------------------------------------------

          2001                                                                                          2100
  Hs_FLG2 ESGSSVHERHGTTHGQTADTTRHGHSGHGQSTQRGSRTTGRRASGHSEYSDSEGHSGVSHTHSGHAHGQAGSQHGESGSSVHERHGTTHGQTGDTTRHAH
  Em_FLG2 ----------------------------------------------------------------------------------------------------
 Tml_FLG2 ----------------------------------------------------------------------------------------------------
  Dd_FLG2 ----------------------------------------------------------------------------------------------------

          2101                                                                                          2200
  Hs_FLG2 SGHGQSTQRGSRTAGRRGSGHSESSDSEVHSGVSHTHSGHTYGQARSQHGESGSAIHGRQGTIHGQTGDTTRHGQSGHGQSTQTGSRTTGRQRSSHSESS
  Em_FLG2 ----------------------------------------------------------------------------------------------------
 Tml_FLG2 ----------------------------------------------------------------------------------------------------
  Dd_FLG2 ----------------------------------------------------------------------------------------------------

          2201                                                                                          2300
  Hs_FLG2 DSEVHSEASPTHSGHTHSQAGSRHGQSGSSGHGRQGTTHGQTGDTTRHAHYGYGQSTQRGSRTTGRRGSGHSESSDSEVHSWGSHTHSGHIQGQAGSQQR
  Em_FLG2 ----------------------------------------------------------------------------------------------------
 Tml_FLG2 ----------------------------------------------------------------------------------------------------
  Dd_FLG2 ----------------------------------------------------------------------------------------------------

          2301                                                                                          2400
  Hs_FLG2 QPGSTVHGRLETTHGQTGDTTRHGHSGYGQSTQTGSRSSRASHFQSHSSERQRHGSSQVWKHGSYGPAEYDYGHTGYGPSGGSRKSISNSHLSWSTDSTA
  Em_FLG2 -------------------------------------------SQPWSSQRQRHGSGQGWKHGSYGRAAYDYGESGYGPSGGSRASNHNSNQEQRCYYFD
 Tml_FLG2 ----------------------------------------------------------------------------------------------------
  Dd_FLG2 ----------------------------------------------------------------------------------------------------

          2401
  Hs_FLG2 NKQLSRH
  Em_FLG2 -------
 Tml_FLG2 -------
  Dd_FLG2 -------
```

D

```
          1                                                                                              100
  Hs_HRNR MPKLLQGVITVIDVFYQYATQHGEYDTLNKAELKELLENEFHQILKNPNDPDTVDIILQSLDRDHNKKVDFTEYLLMIFKLVQARNKIIGKDYCQVSGSK
  Em_HRNR MPKLLPSIVSVIEIYYQYATEEGECNSLNRAELKELLENEFRQILKNPDDPDTVDIIMQSLDRDHNKKVDFTEYLVMIFKLAQACNKIISKDYRQTSGSK
 Tml_HRNR MPKLLSSIVSVIEIYYQYATQDGEWDLLNKAELKELLENEFRHILKNPDDPDTVDVIMQSLDQDRNRKVDFTEYLMMIFKLARACDKIIGKDYRQASGSK
  Dd_HRNR MPKLLSSIVSVIEIYYQYATQDGEWDLLNKAELKELLENEFHHILKNPDDPDTVDVIMQSLDQDRNRKVDFTEYLMMIFKLARACDKIIGKDYRLASGSE

          101                                                                                            200
  Hs_HRNR LRDDTHQHQEEQEETEKEENKRQESSFSHSSWSAGE-NDSYSRNVRGSLKPGTESISRRLSFQRDFSGQHNSYSGQSSSYGEQNSDSHQSSGRGQCGSGS
  Em_HRNR QRHHSYQHQEEQSETEEEE-KGQESSSSHSSWSTGEENDSYSRDSR-SIKHKTGSSSRRLGYQGGLSSSEH----RQSSGERRESSSGYSKGRGKNKHGS
 Tml_HRNR QRNHSYWHQEEQSETEEEQ-KGQESSSSQSSWSTGVENDSSSRGSR-RIHYKTGLSSRRLGNQGGLSSSEH----KQSSGERRKSSSGYSKGRGKSKHGS
  Dd_HRNR QRNHSRWHQEEQSETEEEQ-KGQESSSSQSSWSTGAENDSSSRGSR-CIQYETGFSSRRLGNQGGLSSSEH----RESSGERRKSSLGYSKGRGKSKHAS

          201                                                                                            300
  Hs_HRNR GQSPNYGQHGSGSGQSSSNDTHGSGSGQSSGFSQHKSSSGQSSGYSQHGSGSGHSSGYGQHGSRSGQSSRGERHRSSSGSSSSYGQHGSGSRQSLGHGRQ
  Em_HRNR YQP-------EGSESEEVGSTHSSNHRKRSNSANKSDSCGEQEHISY---SEDQSFSFDQHWSDSNESLGNRQHRRRSDKFPSKQHHGFSS-SSCGGQDS
 Tml_HRNR YQP-------ERSGSEEVGYTHSSNYRKRSNSANESDSCGQQR---------------------------------------------------------
  Dd_HRNR YQP-------GRSGSEEVGYTHSSNYRKRSNSANESDSCGQQR---------------------------------------------------------

          301                                                                                            400
  Hs_HRNR GSGSRQSPSHVRHGSGSGHSSSHGQHGSGSSYSYSRGHYESGSGQTSGFGQHESGSGQSSGYSKHGSGSGHSSSQGQHGSTSGQASSSGQHGSSSRQSSS
  Em_HRNR WTNSNESTDYGQLGFGSGQPSQQRQHEPNAG---------SQSGKCEEQGYHSSSS-ESSSYGKHRSSSGQPSSQKKRGS-----SSSGQSGNWRKE---
 Tml_HRNR --------------FGSGQPSQQRWHEPNTR---------SQSGNCEEQGYHPSSS-EFSSYKKHRSSSGQPSSQRKHGS-----SSSGHSGSWRKE---
  Dd_HRNR --------------FGSGPPSQQRWHEPNAG---------SQSGNCEEQGYHPSSS-EFSSYKKHRSSSGQPSSQRKHGS-----SSSGHSGSWRKE---

          401                                                                                            500
  Hs_HRNR YGQHESASRHSSGRGQHSSGSGQSPGHGQRGSGSGQSPSSGQHGTGFGRSSSSGPYVSGSGYSSGFGHHESSSEHSSGYTQHGSGSGHSSGHGQHGSRSG
  Em_HRNR ---------------KHGSGSGKSSSFEQYGSSSGQSSNCGKHGSSINHSSSQEQHKSSSSGQSG---------HKGQQKQSGFQSGQSSSYEECKSESG
 Tml_HRNR ---------------KHRSGSGNCSSFEQYGSSSGQSPNYGKHGSSTNHSSSQRQDKSSSSSQLG---------HNRQQKQSGFESRESSSYKEYKSESG
  Dd_HRNR ---------------RHRSGSGNCSSFEQYGSSSGQSPNYGKHGSSTNHSSSQKQDKSSSSGQLG---------HNRQQKQSGFESRESSSYKEYKSESG

          501                                                                                            600
  Hs_HRNR QS-SRGERQGSSAGSSSSYGQHGSGSRQSLGHSRHGSGSGQSPSPSRGRHESGSRQSSSYGPHGYGSGRSSSRGPYESGSGHSSGLGHQESRSGQSSGYG
  Em_HRNR QSLGSGKNQPSSFQSSTQRKQSNSSGRQSESCGRQKHGSGSVQSSGCGQQGSGTKQSSSYGHHGSGSGHSSNHAQYETGSGQSSSHSHQKCGSGESSGFG
 Tml_HRNR QS-------------STQRKQSNSGGCQSESCGRQKHKSGSVESPGYGQGGSDSEQSSSYGHHGSGSGHSSSHGQYGTGSGQSSSHIHQEYGSRESSGYR
  Dd_HRNR QS-------------STQRKQSNSFGCQSQSCGRQKHESGSVESPGYGQGGSDSKQPSSYDHHGSGSDHSSSHGQYGTGSGQSSSHIHQEYGSRESSGYR
```

```
         601                                                                                              700
Hs_HRNR  QHGSSSGHSSTHGQHGSTSG-QSSSCGQ--HGATSGQSSSHGQHGSGSSQSSRYGQQGSGSGQSPSRGRHGSDFGHSSSYGQHGSGSGWSSSNGPHGSVS
Em_HRNR  QYGSSSSHSSRLKHHEYNSGGHSGSCGRQKHGSGSSQSPNHGKYGSASNQSS--SQHGFGSGQSSSYEQQRSGSQQSSSYRQHRFGSGQSSSCSQHGSGL
Tml_HRNR QYGSDSSHSSRQKRHEYNSGGHSGICGRQKYRSGSSQSSNYGKYESGSNQSS--SQHGFGSGQSSSYGQHGFGPGQSSSHSQYGSGSGQSSSFEQHKSGS
Dd_HRNR  QYGSGSSHSSRQKRHEYNSGGHSGICGRQKYGSGSSQSSNYGKYESGSNQSS--SQHGFGSGQSSSYGQHGFGPGQSSSHSQYGSGSGQSSSYEQHKSGS

         701                                                                                              800
Hs_HRNR  GQSSGFG-HKSGSGQSSGYSQHGSGSSHSSGYRKHGSRSGQSSRSEQHGSSSGLSSSYGQHGSGSHQSSGHGRQGSGSGHSPSRVRHGSSSGHSSSHGQH
Em_HRNR  GQSSSCGQHGSGSGQTLSHSRHGSGSGQSSSYGQHESCSGQSSSFGQHGSGLGQSSGCSQHRSGSGQSSSYEKHWSGLGQCSSHSKHGSSSGQSSGFGQH
Tml_HRNR GQSSSYGNHGSGSGQFSSHSQHGSGSGQSSGCDQQGSSSGQSSSYGQLGSGSCQSSSQGQHGSGSGQCSSYS-HGSGSGQYSNSEQYGSGSCPSSCSGQY
Dd_HRNR  GQSTSYGNHGSGSGQSSSHSQHGSGSGQSSGCDQQGSSSGQSSSYGQLGSGSCQSSSQGQHGSGSGQCSSYS-HGSGSRQYSNSEQYGSGSYPSSCSGQY

         801                                                                                              900
Hs_HRNR  GSGTSCSSSCGHY-ESGSGQASGFGQHESGSGQG--YSQHGSASGHFSSQGRHGSTSGQSSSSGQHDSSSGQSSSYGQHESASHHASGRGRHGSGSGQSP
Em_HRNR  GSGSVQSSSYGQH-RSGSGQSSSYGQHGSGSGQSSSHSRHGSGSRQLSNCGQQGSGSSQSSSYGQDGSGSGQSSSYGQHESGSCQSSSYSQHGSGLGHSS
Tml_HRNR GSGSGQHSGYEQCGQVESSSGCGFGQFTPYYGQP--RSNTINELSICKEVYRQGGNS-------------------------------------------
Dd_HRNR  GSGSGQHSGYEQCGQVESSSGCGSGQFTPSYGQP--RSNTINKLSICKEVYRQGGNS-------------------------------------------

         901                                                                                             1000
Hs_HRNR  GHGQRGSGSGQSPSYGRHGSGSGRSSSSGRHGSGSGQSSGFGHKSSSGQSSGYTQHGSGSGHSSSYEQHGSRSGQSSRSEQHGSSSGSSSSYGQHGSGSR
Em_HRNR  SHGQNGSSSGQCSSYSQHGSGSGSGSGSG---SGSGSGSGSGSGSGSGQYSNSEQYGSGSCLSSCSEQYDFGSGQHSSYEQRGQVESSSGC---------
Tml_HRNR ----------------------------------------------------------------------------------------------------
Dd_HRNR  ----------------------------------------------------------------------------------------------------

         1001                                                                                            1100
Hs_HRNR  QSLGHGQHGSGSGQSPSPSRGRHGSGSGQSSSYGPYRSGSGWSSSRGPYESGSGHSSGLGHRESRSGQSSGYGQHGSSSGHSSTHGQHGSTSGQSSSCGQ
Em_HRNR  ---GFGQFTTSYGQS------RFNTANKLSICNEVYRQ-SGNCYQRGS----------------------------------------------------
Tml_HRNR ----------------------------------------------------------------------------------------------------
Dd_HRNR  ----------------------------------------------------------------------------------------------------

         1101                                                                                            1200
Hs_HRNR  HGASSGQSSSHGQHGSGSSQSSGYGRQGSGSGQSPGHGQRGSGSRQSPSYGRHGSGSGRSSSSGQHGSGLGESSGFGHHESSSGQSSSYSQHGSGSGHSS
Em_HRNR  ----------------------------------------------------------------------------------------------------
Tml_HRNR ----------------------------------------------------------------------------------------------------
Dd_HRNR  ----------------------------------------------------------------------------------------------------

         1201                                                                                            1300
Hs_HRNR  GYGQHGSRSGQSSRGERHGSSSGSSSHYGQHGSGSRQSSGHGRQGSGSGHSPSRGRHGSGLGHSSSHGQHGSGSGRSSSRGPYESRSGHSSVFGQHESGS
Em_HRNR  ----------------------------------------------------------------------------------------------------
Tml_HRNR ----------------------------------------------------------------------------------------------------
Dd_HRNR  ----------------------------------------------------------------------------------------------------

         1301                                                                                            1400
Hs_HRNR  GHSSAYSQHGSGSGHFCSQGQHGSTSGQSSTFDQEGSSTGQSSSYGHRGSGSSQSSGYGRHGAGSGQSPSRGRHGSGSGHSSSYGQHGSGSGWSSSSGRH
Em_HRNR  ----------------------------------------------------------------------------------------------------
Tml_HRNR ----------------------------------------------------------------------------------------------------
Dd_HRNR  ----------------------------------------------------------------------------------------------------

         1401                                                                                            1500
Hs_HRNR  GSGSGQSSGFGHHESSSWQSSGCTQHGSGSGHSSSYEQHGSRSGQSSRGERHGSSSGSSSSYGQHGSGSRQSLGHGQHGSGSGQSPSPSRGRHGSGSGQS
Em_HRNR  ----------------------------------------------------------------------------------------------------
Tml_HRNR ----------------------------------------------------------------------------------------------------
Dd_HRNR  ----------------------------------------------------------------------------------------------------

         1501                                                                                            1600
Hs_HRNR  SSYSPYGSGSGWSSSRGPYESGSSHSSGLGHRESRSGQSSGYGQHGSSSGHSSTHGQHGSTSGQSSSCGQHGASSGQSSSHGQHGSGSSQSSGYGRQGSG
Em_HRNR  ----------------------------------------------------------------------------------------------------
Tml_HRNR ----------------------------------------------------------------------------------------------------
Dd_HRNR  ----------------------------------------------------------------------------------------------------

         1601                                                                                            1700
Hs_HRNR  SGQSPGHGQRGSGSRQSPSYGRHGSGSGRSSSSGQHGSGLGESSGFGHHESSSGQSSSYSQHGSGSGHSSGYGQHGSRSGQSSRGERHGSSSRSSSRYGQ
Em_HRNR  ----------------------------------------------------------------------------------------------------
Tml_HRNR ----------------------------------------------------------------------------------------------------
Dd_HRNR  ----------------------------------------------------------------------------------------------------

         1701                                                                                            1800
Hs_HRNR  HGSGSRQSSGHGRQGSGSGQSPSRGRHGSGLGHSSSHGQHGSGSGRSSSRGPYESRSGHSSVFGQHESGSGHSSAYSQHGSGSGHFCSQGQHGSTSGQSS
Em_HRNR  ----------------------------------------------------------------------------------------------------
Tml_HRNR ----------------------------------------------------------------------------------------------------
Dd_HRNR  ----------------------------------------------------------------------------------------------------

         1801                                                                                            1900
Hs_HRNR  TFDQEGSSTGQSSSHGQHGSGSSQSSSYGQQGSGSGQSPSRGRHGSGSGHSSSYGQHGSGSGWSSSSGRHGSGSGQSSGFGHHESSSWQSSGYTQHGSGS
Em_HRNR  ----------------------------------------------------------------------------------------------------
Tml_HRNR ----------------------------------------------------------------------------------------------------
Dd_HRNR  ----------------------------------------------------------------------------------------------------

         1901                                                                                            2000
Hs_HRNR  GHSSSYEQHGSRSGQSSRGEQHGSSSGSSSSYGQHGSGSRQSLGHGQHGSGSGQSPSPSRGRHGSGSGQSSSYGPYGSGSGWSSSRGPYESGSGHSSGLG
Em_HRNR  ----------------------------------------------------------------------------------------------------
Tml_HRNR ----------------------------------------------------------------------------------------------------
Dd_HRNR  ----------------------------------------------------------------------------------------------------
```

```
          2001                                                                                               2100
  Hs_HRNR HRESRSGQSSGYGQHGSSSGHSSTHGQHGSASGQSSSCGQHGASSGQSSSHGQHGSGSSQSSGYGRQGSGSGQSPGHGQRGSGSRQSPSYGRHGSGSGRS
  Em_HRNR ----------------------------------------------------------------------------------------------------
 Tml_HRNR ----------------------------------------------------------------------------------------------------
  Dd_HRNR ----------------------------------------------------------------------------------------------------

          2101                                                                                               2200
  Hs_HRNR SSSGQHGPGLGESSGFGHHESSSGQSSSYSQHGSGSGHSSGYGQHGSRSGQSSRGERHGSSSGSSSRYGQHGSGSRQSSGHGRQGSGSGHSPSRGRHGSG
  Em_HRNR ----------------------------------------------------------------------------------------------------
 Tml_HRNR ----------------------------------------------------------------------------------------------------
  Dd_HRNR ----------------------------------------------------------------------------------------------------

          2201                                                                                               2300
  Hs_HRNR SGHSSSHGQHGSGSGRSSSRGPYESRSGHSSVFGQHESGSGHSSAYSQHGSGSGHFCSQGQHGSTSGQSSTFDQEGSSTGQSSSHGQHGSGSSQSSSYGQ
  Em_HRNR ----------------------------------------------------------------------------------------------------
 Tml_HRNR ----------------------------------------------------------------------------------------------------
  Dd_HRNR ----------------------------------------------------------------------------------------------------

          2301                                                                                               2400
  Hs_HRNR QGSGSGQSPSRGRHGSGSGHSSSYGQHGSGSGWSSSSGRHGSGSGQSSGFGHHESSSWQSSGYTQHGSGSGHSSSYEQHGSRSGQSSRGERHGSSSGSSS
  Em_HRNR ----------------------------------------------------------------------------------------------------
 Tml_HRNR ----------------------------------------------------------------------------------------------------
  Dd_HRNR ----------------------------------------------------------------------------------------------------

          2401                                                                                               2500
  Hs_HRNR SYGQHGSGSRQSLGHGQHGSGSGQSPSPSRGRHGSGSGQSSSYSPYGSGSGWSSSRGPYESGSGHSSGLGHRESRSGQSSGYGQHGSSSGHSSTHGQHGS
  Em_HRNR ----------------------------------------------------------------------------------------------------
 Tml_HRNR ----------------------------------------------------------------------------------------------------
  Dd_HRNR ----------------------------------------------------------------------------------------------------

          2501                                                                                               2600
  Hs_HRNR TSGQSSSCGQHGASSGQSSSHGQHGSGSSQSSGYGRQGSGSGQSPGHGQRGSGSRQSPSYGRHGSGSGRSSSSGQHGSGLGESSGFGHHESSSGQSSSYS
  Em_HRNR ----------------------------------------------------------------------------------------------------
 Tml_HRNR ----------------------------------------------------------------------------------------------------
  Dd_HRNR ----------------------------------------------------------------------------------------------------

          2601                                                                                               2700
  Hs_HRNR QHGSGSGHSSGYGQHGSRSGQSSRGERHGSSSGSSSHYGQHGSGSRQSSGHGRQGSGSGQSPSRGRHGSGLGHSSSHGQHGSGSGRSSSRGPYESRLGHS
  Em_HRNR ----------------------------------------------------------------------------------------------------
 Tml_HRNR ----------------------------------------------------------------------------------------------------
  Dd_HRNR ----------------------------------------------------------------------------------------------------

          2701                                                                                               2800
  Hs_HRNR SVFGQHESGSGHSSAYSQHGSGSGHFCSQGQHGSTSGQSSTFDQEGSSTGQSSSYGHRGSGSSQSSGYGRHGAGSGQSLSHGRHGSGSGQSSSYGQHGSG
  Em_HRNR ----------------------------------------------------------------------------------------------------
 Tml_HRNR ----------------------------------------------------------------------------------------------------
  Dd_HRNR ----------------------------------------------------------------------------------------------------

          2801                                                      2859
  Hs_HRNR SGQSSGYSQHGSGSGQDGYSYCKGGSNHDGGSSGSYFLSFPSSTSPYEYVQEQRCYFYQ
  Em_HRNR --------------------------NCGRGSTDSISHSFCSSTPLYEYVQERNNKQK-
 Tml_HRNR ---------------------FLRGGNCRRGSTDSIFHSFCSSTPLYAYVKEQRHYF--
  Dd_HRNR ---------------------FLRGGNCRRGSTDTISHSFCSSTPLYAYVKEQRHYF--
```

E

```
          1                                                                                                  100
  Hs_RPTN MAQLLNSILSVIDVFHKYAKGNGDCALLCKEELKQLLLAEFGDILQRPNDPETVETILNLLDQDRDGHIDFHEYLLLVFQLVQACYHKLDNKSHGGRTSQ
  Em_RPTN MAQLLSSILTVIKVFQKHASENGDCTSLCKKELKQLLLAEFGDILWRPNDPETVETILTILDRDSNGHIDFHEYLLLVFQLAQACYHKLDIQSCGDRTSQ
 Tml_RPTN MAQLLNSILTVIKVFQKHAKENGDCASLCKKELKQLLLAEFGDILRRPNDPETVETILSILDRDRNGRVDFHEYLLLVFQLVQACYRKLDIESYGDRTSR
  Dd_RPTN MAQLLNSILIVIKVFQEHAKENGDCASLCKKELKQLLLAEFGDILRRPNDPETVETILSILDRDRNGRVDFHEYLLLVFQLVQACYRELDIESCGDRTSQ

          101                                                                                                200
  Hs_RPTN QERGQEGAQDCKFPGNTGRQHRQRHEEERQNSHHSQPERQDGDSHHGQPERQDRDSHHGQSEKQDRDSHHSQPERQDRDSHHNQSERQDKDFSFDQSERQ
  Em_RPTN QEEEQEGVQDHTFPRNRGRQHRQRHEEERQHSHHGHSERQDRDSHHSGSERQERDSSYGQSERQDQDSYYDQSERQERDSHYGQSERQGWDSRHGQSERY
 Tml_RPTN QEEGQEGAQDHKFPRNRDRQHRHRQEEERQDSHHGQSERQDRDSCHDQSEKQDRNSRHGQSEGQDRDSHHGQSERQDQDFHHGQSERQDRDSSLNQSERQ
  Dd_RPTN QEEGQEGAQDHKFPRNRGRQHRHRQEEERQDSHHGQSERQDRDSCHDQSERQDRNSRHGQSERQDQDFCHGQSEGQDRDSSLNQSERQVQDSSYGQ----

          201                                                                                                300
  Hs_RPTN SQDSSSGKKVSHKSTSGQAKWQGHIFALNRCEKPIQDSHYGQSERHTQQSETLGQASHFNQTNQQKSGSYCGQSERLGQELGCGQTDRQGQSSHYGQTDR
  Em_RPTN NLDSHYS----------QPERQDRDSSLNQSERQGQDSSYGQKLSHKSRQ---GYLFALNQCEKPVQDSHHNQSESLGQRSSCGQSRRLGQDSCSSHTEQ
 Tml_RPTN GQDSSYG----------Q--------------KLSHKSSNGQP----KRQ---GYLFAVNQCEKPVQDSHHNQSERLGLRSSCGQTRRLGQDTCSSRTEQ
  Dd_RPTN --------------------------------KLSRKSSNGQP----KRQ---GYLFAVNQCEKPFQDSHHNQSEKLGQRSSCGQCRRLGQDSCSSHTEQ

          301                                                                                                400
  Hs_RPTN QDQSYHYGQTDRQGQSSHYSQTDRQGQSSHYSQPDRQGQSSHYGQMDRKGQCYHYDQTNRQGQGSHYSQPNRQGQSSHYGQPDTQDQSSHYGQTDRQDQS
  Em_RPTN QQTGCIYGQSGRLDQESGCGQKHRQGLDSEY------------------------------------------------------------GQTDRQDQS
 Tml_RPTN QESGSIYGQSGRLAQESGCGQRDRQGLDSQY------------------------------------------------------------GQTDRQ---
  Dd_RPTN QESGSICGQSGRLAQESGCGQRDRQGLDSHY------------------------------------------------------------GQTDRQ---

          401                                                                                                500
  Hs_RPTN SHYGQTERQGQSSHYSQMDRQGQGSHYGQTDRQGQSSHYGQPDRQGQNSHYGQTDRQGQSSHYGQTDRQGQSSHYSQPDKQGQSSHYGKIDRQDQSYHYG
  Em_RPTN PHYCQTGRQGQSSHYGQTDRPGQSSHYGQTDRQDQSSRYGQADRRGQSSHYGQTDRQGQSSDYGQTDRQGQSSCYGQRDRQGHSSRYGQADRRGQSSHDG
 Tml_RPTN ----------------------------------------------------------------------------------------------------
  Dd_RPTN ---------------------------------------------GQSSHYEQIDRQGQSSLHGQTDKQGLSS---------------------------
```

```
           501                                                                                              600
  Hs_RPTN  QPDGQGQSSHYGQTDRQGQSFHYGQPDRQGQSSHYSQMDRQGQSSHYGQTDRQGQSSHYGQTDRQGQSYHYGQTDRQGQSSHYIQSQTGEIQGQNKYFQG
  Em_RPTN  QTDRPGQSSHYGQTDRQDQSSHYGQTDRQGQSSHDGQTDRQGQSSHDGQTDKEVHSSHYGQTET-------GET---------------EIQGQDRHFQG
 Tml_RPTN  -----------------------------GQGYH------------------------YGQRET-------GET---------------EIQGQNRYFQG
  Dd_RPTN  ---------HYGQTDRQGQSSHYGQTDRQGQGYH------------------------YGQRET-------RET---------------EIQGQNRYFQG

           601                                                                                              700
  Hs_RPTN  TEGTRKASYVEQSGRSGRLSQQTPGQEGYQNQGQGFQSRDSQQNGHQVWEPE-EDSQHHQHKLLAQIQQERPLCHKGRDWQSCSSEQGHRQAQTRQSHGE
  Em_RPTN  TEGTRRDTYVEQSGRSGRLSQQTQGQEVNRNQRQGSQNR------QQAWKRQLEDTQHHQHKLLAQIQQERSLCHKGRDWQSSGSEQGHRQAETQQSHSQ
 Tml_RPTN  SEGIRRDTHVEQSGRSRRLNQQTQGQEVNPNQRQGSQKR------LQAWERQLEDTQHHQHNLLAEIQQERSLCHKRRDWQSSGSEQDHRQAQTQQSHSE
  Dd_RPTN  SEGTRRDTHVEQSGRSGRLNQQTQGQEVNPNQRQGSQNR------LQAWERQLEDTQHHQHNLLAQIQQERSLCHKRRDWQSSGSEQGHRQAQTQQSHSE

           701                                                                                              800
  Hs_RPTN  GLSHWAEEEQGHQTWDRHSHESQEGPCGTQDRRTHKDEQNHQRRDRQTHEHEQSHQRRDRQTHEDKQNRQRRDRQTHEDEQNHQR---------------
  Em_RPTN  -----TEERQGHQSWGIHSHEGQESPREARDRQTHKEEQSCQTRDRQSHEDEQNHQRQDRQTHEDEQNHQRQDRQTHEDEQNHQREDWQTHEGEQDHQRQ
 Tml_RPTN  GKSHRTEERQSHQSWGRQSHEGQESPCEAQDGQTREEEQSCQTRDKQTREDKQNHQRQDRQTHEDEQNRQRQIKQI------------------------
  Dd_RPTN  GKSHRTEDRQSHQSWGRQSHEGQKSPCEAQDGQTHEEEQSCQTRDKQTREDEQNHQRQDKQTHEDEQNRQRQIKQTREDKQN------------------

           801                                                                                              900
  Hs_RPTN  ----------------------------------------------------------------------------------------------------
  Em_RPTN  DRQTQEDEQNRQRQDRQTHEGEQNHQRQDWRTHEGEQNRQRQDRQTHEDEQNHQRQDRQTHEDEQNHQRQDWQTHEGEQDHQRQDRQTHEDEQNRQRQDR
 Tml_RPTN  ----------------------------------------------------------------------------------------------------
  Dd_RPTN  --------------------------------------RQRQDRQTHENKQNCQRQDRQTHEDEQKRQRQIRQTREVEQNQQRQDKQTHEDKKTRQKQDR

           901                                                                                             1000
  Hs_RPTN  ----------------------------------------------------------------------------------------------------
  Em_RPTN  QTHEGEQNHQRQDWRTHEGEQNRQRQDRQTHEGEQNRQRQDRQTHEGEQNRQRQDRQTHEGEQNRQRQDRQTHEGEQNRQRGNRQIHEEDQNCWQQQNTQ
 Tml_RPTN  --------------------------------------------HEDEQNRQTQDRQTHENKQNCQRQDRQTHEDEQNHQRRDRQTDEEDQNLRRQQNTQ
  Dd_RPTN  QIHENEQNHQKQD----------------------------RQTHEDEQNRQRQDRQTHENKQNCQKQDRQTHEDEQNHQRRERQTHEEDQNLRRQQNTQ

           1001                                                                                   1090
  Hs_RPTN  ------------------------------------------------------------------------------------------
  Em_RPTN  THEGEERYDRSQNQQFNVTQQGCSNRETFHLNEGGQSRVSQGRRSDQAFHSTQGNGRPQRREQDRDHPTKAAISSNPLYDYVQEQRSHQY
 Tml_RPTN  TYDGEERYERSQNQQSHGTQQGCSNRETFHMNEGGLSRGSQGRRSDPALHPTQGNGRPQRREQGGGHPTKAAISSNPLYDYVQEQRSHQY
  Dd_RPTN  TYDREERYERSQNQQSHGTQQGCSNRETFHMNEGGLSRGSQGRRSDPALHPTQGNGKPQRREQGGGHPTKAAISSNPLYDYVQEQRSHQY
```

F

```
           1                                                                                                100
  Hs_TCHH  MSPLLRSICDITEIFNQYVSHDCDGAALTKKDLKNLLEREFGAVLRRPHDPKTVDLILELLDLDSNGRVDFNEFLLFIFKVAQACYYALGQATGLDEEKR
  Em_TCHH  MPALLRSICDITEIFNQYASNDCDRAALCKKDLKELLEREFGDVLRRPHDPETVDLVLELLDRDHNGLIDFHEYLLLIFRMAQACYYALSQATGLDEEKG
 Tml_TCHH  MSALLRSIFDITEIFNQCASNDCNGAGLHKKDLKELLEREFGD----PHDPETVALALELLDRDFNGLIDFHEYLLLIFRIAQACYYALGQATGLEEEKG
  Dd_TCHH  MSALLRRIFDITEIFNQCASNDCNGAGLHKKDLKELLEREFGDVLRRPHDPETVALALELLDRDWNGLIDFHEYLLLIFRIAQACYYSLGQATGLEEQKG

           101                                                                                              200
  Hs_TCHH  ARCDGKESLLQDRRQEEDQRRFEPRDRQLEEEPGQRRRQKRQEQERELAEGEEQSEKQERLEQRDRQRRDEELWRQRQEWQEREE-RRAEEEQLQSCKGH
  Em_TCHH  AKYEGKRNPLNDRRQE-DQRRFERRQRLVEEE------RKQNGQERELVEEELQRERQQRREQRDPQRRDEG---QRLQRREGLE-RREEKGLLQRRQGR
 Tml_TCHH  AKYEGKGNPLNDRRQE-DQRRFERRDRPVEEE----RRHKRQERARELVEEELQ---------TDKQRRDEAQRLQRLEWLEREEERREEIEQLPRRKGR
  Dd_TCHH  AKYEGKGNPMNDRRQE-DQRRFERRDRPVEEE----RRRKRQEQARELVEEELQ---------RDKQRRDKAQSLQRLEWLEREEERREEIEQLQRRKGR

           201                                                                                              300
  Hs_TCHH  ETEEFPDEEQLRRREL---LELRRKGREEKQQQRRERQDRVFQEEEEKEWRKRETVLRKEEEKLQEEEPQRQRELQEEEEQLRKLERQELRRERQEEEQQ
  Em_TCHH  EPAEFPEEEQLQRRER---LEQRERQVEEEQLQR---------------------------ERQVEEERRQRRERLEQELQLPRRGRDEPRAQRERREQE
 Tml_TCHH  E----PEDEQLQRRER---QGQREPLRLEEELQRRREPLERQEEEEQLQRRER----RELQRERQDEEEQLQRERQQEAERRQRREGNELRLEEQLQRRE
  Dd_TCHH  E----PEDEQLQRRERRERQEQREPLRREEELQRRREPLERQEEEEQLQRRER----RELQRERQDEEEQLQRERQQEAERRQRREGNELRLEEQLQRRE

           301                                                                                              400
  Hs_TCHH  QQRLRREQQLRRKQEEERREQQEERREQQERREQQEERREQQLRREQEERREQQLRREQEEERREQQLRREQEEERREQQLRREQQLRREQQLRREQQLR
  Em_TCHH  ----RREQELAEEEQQELGESRASRWQWQP--ESEADARHSKVRSRPGRQEEQRRRLEQEEESRRCQ--------EPEQQLRRELEDDARRQAR-PGQLR
 Tml_TCHH  Q-RERREQELAEQEEPELAESRTPRWQWQL--ESEADARQSKAYSRPRGQEEQRRRLEQEEEQRRRQ--------ERERQLRRE-QDDAQRQAQ-PGQLR
  Dd_TCHH  Q-RERREQELAEQKEPELAESGTPRWQWQL--ESEADARQSKAYSRPRGQEEQRRRLEQEEEQRPRQ--------ERERQLRRE-QDDAQRQAR-PAQLR

           401                                                                                              500
  Hs_TCHH  REQQLRREQQLRREQQLRREQQLRREQQLRREQEEERHEQKHEQERREQRLKREQEERRDWLKREEETERHEQERRKQQLKRDQEEERRERWLKLEEEER
  Em_TCHH  EERERRDFQWQLEEEREKRRQRLSARSSLK--EQRERQARAKEGQERDGRLGEEEDQRRD----REELQFLEEEEQLQRRRRAGQFQ--------EEGNL
 Tml_TCHH  QE-QRRDFQWQQEEERQRRRQRLSTRPSLK--GQRKRELRADESQKRELLL-REEEQRCQRRQRAQEPQFREEEEQLQRQERDRQD--------LEEELL
  Dd_TCHH  EE-QRRDFQWQLEEERERPRQRLSARPSLK--GQREREWRADERQKRELLL-REAELRCQRRQRAQEPQFREEEEQLQRQERDRQD--------LEEELL

           501                                                                                              600
  Hs_TCHH  REQQERREQQLRREQEERREQRLKRQEEEERLQQR-LRSEQQLRREQEERREQLLKREEEKRLEQERREQRLKREQEERRDQLLKREEERRQQRLKREQE
  Em_TCHH  QEDQEIR----RRQEDQGRDQKWKWQLEEESQRRRHLLYSKSAKQEQLQRQARDRQYGEEDLLQQE--EEQLQREQREKR----RRQEPERQYR----EE
 Tml_TCHH  QREEREK----RRLQERERQYREEQQLEQEEERL------QREERE--------RQYREEEQLEQE--EERLQREERE------------RQYR----EE
  Dd_TCHH  QREEREK----RRRQEQVRQYRDEEQLEQEEERL------QREEREKRRHQEQVRQYRDEEQLEQE--EERLQREEREKR----RRQEQVRQYR----DE

           601                                                                                              700
  Hs_TCHH  ERLEQRLKREEVERLEQEERREQRLKREEPEEERRQQLLKSEEQEERRQQQLRREQQERREQRLKREEEEERLEQRLKREHEEERREQELAEEEQEQARE
  Em_TCHH  EQLQQGEEQLQRERRLQERDRKYR-EEEEPQRQERKQQFRDEDQCRDLKRQPEKENEVRKNRENEEKARQLEESQVLERQFQQDRRPQQDELEEELSLQE
 Tml_TCHH  EQLEQEEERLQRE----------------RGRQERERQYREDEELQLEEEWLQREERKKRRRQEQM---------------RQYREKQQLEQEEE-----
  Dd_TCHH  EQLEQEEERLQRE------------EREKRRRQEQVRQYRDEEQLEQEEERLQREERERQYREEQQLEQEEERLQREERE-RQYREEQQLEQEEE-----
```

```
         701                                                                                              800
Hs_TCHH  RIKSRIPKWQWQLESEADARQSKVYSRPRKQEGQRRRQEQEEKRRRRESELQWQEEERAHRQQQEEEQRRDFTWQWQAEEKSERGRQRLSARPPLREQRE
Em_TCHH  R--------------EQERRRWLQRERQFPTEVPLRGEEQEKAKRL---DGKFREEEQLLREGTEEKRRRQEGDRRFPEESFQQSRDR-------RFQEE
Tml_TCHH -----------------------------------------------------------------------------------------------RPQRE
Dd_TCHH  -----------------------------------------------------------------------------------------------RMQRE

         801                                                                                              900
Hs_TCHH  RQLRAEERQQREQRFLPEEEEKEQRRRQRREREKELQFLEEEEQLQRRERAQQLQEEEDGLQEDQERRRSQEQRRDQKWRWQLEEERKRRRHTLYAKPAL
Em_TCHH  EKLR---RQERDRKFREEEQRLQER---------------EEEQVRRQLRDRQFREEEQQLDQELEEERLRRQQRDRNFREEGQMRLERE----------
Tml_TCHH RR-----RQERGRQYR------------------------EEEELQRQDRKRQFRDEDQRRDLKRQLGKENEAGKNRVYSKSGKNEEKAR----------
Dd_TCHH  RR-----RQERGRQYR------------------------EEEELQRQDRKRQFRDEDQRGNLKRQLGKENEAGKNRVYSKSGKNEEKAR----------

         901                                                                                             1000
Hs_TCHH  QEQLRKEQQLLQEEEEELQREEREKR--RRQEQERQYREEEQLQQEEEQLLREEREKRRRQERERQYRKDKKLQQKEEQLLGEEPEKRRRQEREKKYREE
Em_TCHH  EQQLRRQQQDRNFPEEEQLRQERENQQLRRQQRDRKFLEDEQLSQERED------EQLRRQERDRKFRED-------EQLRRQEREQQLPQERDRKFLEE
Tml_TCHH -------------QSEDSQVRERQFQQDRRSQQDE--LEEERSFQEREE------ERRRRQQRDRQLQTA-------DPLEGEEQEEAKRP--DGMFREE
Dd_TCHH  -------------QSEDSQVRERQFQQDRRPEQDE--LEEERSLQEREQ------ERRRRQQRDRQLQTA-------DPLEGEEQEEAKRP--DGMFREE

         1001                                                                                            1100
Hs_TCHH  EELQQE--EEQLLREEREKRRRQEWERQYRKKDELQQEEEQLLREEREKRRLQERERQYREEEELQQEEEQLLGEERETRRRQELERQYRKEEELQQEEE
Em_TCHH  EELRQEREEQQLRRQEREQQLRQERDRQFR-------EVEELRRQQREQQRRQERDRQFREVKELRQERED------EPLRRQQRDRKFREDEQLRQERE
Tml_TCHH EQLLREVREDRRRRQEGDRRFPEESFQQAR----------DRRFQEEKQLRRQERDRKFREEEQA---------------RRPLRDRQFRQEEQHLGQEL
Dd_TCHH  EQLLREVREDRRRRQEGDRRFPEESFQQAR----------DRRFQEEEQLRRQERERKFREEEQV---------------HRPLRDRQFREEEQ------

         1101                                                                                            1200
Hs_TCHH  QLLREEPEKRRRQERERQCREEEELQQEEEQLLREEREKRRRQELERQYREEEEVQQE--EEQLLREEPEKRRRQELERQYREEEELQQEEEQLLREEQE
Em_TCHH  -------EQQLRQERDRQFREVEELRQERED------EQLNRQQRDRKFREDEQLLQEREEQQLRRQEQEQQRRQELDRQFREVEELRQE-----REERQ
Tml_TCHH -----EEEQLRRQERDRNLRGEQQLR---------------RQQRDRKFHEDDQL----------RSQEREQLRQERDRKSREVEEPRQQ------RKDE
Dd_TCHH  -----------------------QLR---------------RQQRDRKFHEDDQL----------RSQER-------DRKSREVEELRQE------RKDD

         1201                                                                                            1300
Hs_TCHH  KRRQERERQYREEEELQRQKRKQRYRDEDQRSDLKWQWEPEKENAVRDNKVYCKGRENEQFRQLEDSQLRDRQSQQDLQHLLGEQQERDREQERRRWQQR
Em_TCHH  LRRQEREQQLRRE-------RDRQFRDVEE---LRQEREDEQLNRQQRDR---KFREDEQLLQERE-----------------EQQLRRQEQEQQRRQER
Tml_TCHH LLRQKRDRQVREDEQLRRQERDRKFR----------------------------------------------------------------EEEELRLQQR
Dd_TCHH  LLRQERDRQVRE-------------------------------------------------------------------------------EEELRLQQR

         1301                                                                                            1400
Hs_TCHH  DRHFPEEEQLEREEQKEAKRRDRKSQEEKQLLREEREEKRRRQETDRKFREEEQLLQEREEQPLRRQERDRKFREEELRHQEQGRKFLEEEQRLRRQERE
Em_TCHH  DRQFREVEELRQE------------REERQLRRQEREQQLP-QERDRKFLEEEELRQEREEQQLRRREREQQLR------QERDRQFREVEE-LRRQQRE
Tml_TCHH DRQLREDE---------------------QLRRQEREQQLR----------QGRDRKSREDEQLRRQERDRQFRED------------------------
Dd_TCHH  DRQLREDE---------------------QLRRQER----------------------------------------------------------------

         1401                                                                                            1500
Hs_TCHH  RKFLKEEQQLRCQEREQQLRQDRDRKFREEEQQLSRQERDRKFREEEQ-QVRRQERERKFLEEEQQLRQERHRKFREEEQLLQEREEQQLHRQERDRKFL
Em_TCHH  --------QQRRQERDRQFREVKELRQEREDEPLRRQQRDRKFREDEQLRQEREEQQLRRQEQEQQRRQERDRQFREVEELRQEREERQLRRQER-----
Tml_TCHH --------QELRQERKDELLRQERDRKSREDEQLRRQERDRKLLEEEELRRERDEQQLRGQECEQQLRQERDRTFREVDELRLEHEDKQLRRQER-----
Dd_TCHH  ---------------------------------------DRKLREEEELRQERDEQQLR--------RQERDRQFREVEELR------------------

         1501                                                                                            1600
Hs_TCHH  EEEQQLRRQERDRKFREQELRSQEPERKFLEEEQQLHRQQRQRKFLQEEQQLRRQERGQQRRQDRDRKFREEEQLRQEREEQQLSRQERDRKFRLEEQKV
Em_TCHH  --EQQLRR-ERDRQFREVEELRQE------REDEQLRRQQRDRKFREDEQLLQEREEQQLRRQEREQQLRQERD-RRFREVEELRRQQRDRKFR-EDEQL
Tml_TCHH -----------DRKLSEEEELRQE------REEQQLRRQERDRQFRQVEEL---------RLQQRDTQLRED---------EQLRRQERDRRFR-EEEEL
Dd_TCHH  ---------------------RQE------REQQL--RQERDRQFREDEQL---------RRQERDRQFREE---------EELRRQQRDRQLR-EEEEL

         1601                                                                                            1700
Hs_TCHH  RRQEQERKFMEDEQQLRRQEGQQQLRQERDRKFREDEQLLQEREEQQLHRQERDRKFLEEEPQLRRQEREQQLRHDRDRKFREEEQLLQEGEEQQLRRQE
Em_TCHH  RRER-------DDEQLRRREREQQYRQERDRKFREEEELRQEREEQQFRRQE------------------QQLRQERDRKFREDEQLRQEREKEQLHRQE
Tml_TCHH RL------------------------QQRDTQLREDEQLRLEREEQQLRRQER----------------EQQLRQERDRRFREEEELRQEREEQQLRGQE
Dd_TCHH  RR------------------------QQRDRQLREDKQLRLEREEQQLRRQER----------------EQQLRQERDRQFREDKELRQEREEQQLRGQE

         1701                                                                                            1800
Hs_TCHH  RDRKFREEEQQLRRQERERKFLQEEQQLRRQELERKFREEEQLRQETEQEQLRRQERYRKILEEEQLRPEREEQQLRRQERDRKFREEEQL-RQEREEQQ
Em_TCHH  REQQLRQERDRNLREVEELRQEQEDEQVRRQQRDRRFREEDELRQEQEELQLSRQERDRRFREEQQLRQEGEDAQLRRQELDAAFSQDEQLNRAEQEEEQ
Tml_TCHH REQQLRQER------------------------DRRFREVEELRLERAEQQL-REERDRQLREEQQLRQERKEEKLRRQELDGAFSQDEQLNRAEQEEEQ
Dd_TCHH  R--------------------------------DRQFREVEELRLEREEQQL-REERDRQLREEQQLRQERKEEKLRCRELDGAFSQDEQLNRAEQEEEQ

         1801                                                                                            1900
Hs_TCHH  LRSQESDRKFREEEQ-LRQEREEQQLRPQQRDGKYRWEEEQLQLEEQ-EQRLRQERDRQYRAEEQFATQEKSRREEQELWQEEEQKRRQERERKLREEHI
Em_TCHH  RRWRQKGSKFLEEEESLQQEREEEKRRRREQDRKFRQEEEQLRREEQQEQRHRQERDRQYRAEEQFA-RDKRRRQEQELRQEEEQRRRQERERKLREE--
Tml_TCHH RRWRQRGSKFLEEEERLHQEREEEKRRRREQDRQFLQEEEQLRREEQEELRRRQERDRQYRAEEQFA-RDTRRQQEQEPRQEEEQRRRQERERKLRGE--
Dd_TCHH  RRWRQRGSKFLEEEERLLQEREEEKRRRREQDRQFLQEEEPLRREEQEELRRRQERDRQYPAEEQFA-RDTRRQQEQELRQEEAQRRRQERERKLRGE--

         1901                                                     1958
Hs_TCHH  RRQQKEEQRHRQVGEIKSQEGKGHGRLLEPGTHQFASVPVRSSPLYEYIQEQRSQYRP
Em_TCHH  ----------------------------DQGKRQFVNPPVRSSPLYEYIQEQRSQYRP
Tml_TCHH ----------------------------DKGKRQFVDPVVRSSPLYEYIQEQRSQYRP
Dd_TCHH  ----------------------------DKGKRQFVDPVVRSSPLYEYIQEQRSQYRP
```

G

```
              1                                                                                                  100
 Hs_TCHHL1    MPQLLRNVLCVIETFHKYASEDSNGATLTGRELKQLIQGEFGDFFQPCVLHAVEKNSNLLNIDSNGIISFDEFVLAIFNLLNLCYLDIKSLLSSELRQVT
 Em_TCHHL1    MPRLLRRVLCVIETFHKYAREDGDGVTLTHRELKQLLQGEFGDILQPHVMHAVEKNVNLLDIGSDGTIRFDQFVLATCNLLNHCYLDIQSL-NSEPRQAP
Tml_TCHHL1    MPRLLRSVLCVIETFHKYAREDGNGVTLTCRELKQLLQGEFGDILQPHVIHAMEKNVNLLDIGSDGTISFDEFVLATCNLLNHCYLDIQSL-NSEPRQVS
 Dd_TCHHL1    MPRLLRSVLCVIETFHKYAREDGNGVTLTCRGLKQLPQGESGGILQPHVIHALEKNVNLLDIGSDGTISFDEFVLATCNLLNHCYLDIQSL-NSEPRQVS

              101                                                                                                200
 Hs_TCHHL1    KPEKEKLDDVDVQATTGDGQWTVGTSPTQEKRMLPSGMASSSQLIPEESGAVGNNRVDPWREAKTHNFPGEASEHNDPKNKHLEGDEQSQEVAQDIQTTE
 Em_TCHHL1    KPEKENPGDVDPQATSRSGQLTEETPPTQDKVVLPSGMAPSSQLNPEEREPVEHNRVDPQEDSKTHNLPGEASEHNDSKNQHLEGDEQIQEVAQHVQTAG
Tml_TCHHL1    KPERKNPDDVDPQATIRNVQLTEETPPTQDKVVLPSGMAQSSQLNPEEKKRVEHNRVDPQEDFKTHNLPREASEHNDSENQHLEGDEQIQEVAQDVQAAG
 Dd_TCHHL1    KPEKKNPDDMDPQATSRNVQLTEETPPTQDKAVLPSGMAQSSQLNPEEKKRVEHNRVDPQEDFKTHNLPREASEHNDSKNQHLEGDEQIQEVAQDVQAAG

              201                                                                                                300
 Hs_TCHHL1    DNEGQLKTNKPMAGSKKTSSPTERKGQDKEISQEGDEPAREQSVSKIRDQFGEQEGNLATQSSPPKEATQRPCEDQEVRTEK--EKHSNIQEPPLQREDE
 Em_TCHHL1    DNGAQLETNKEMVTSKQTSSPTEVEGQDKEIPREAEKPAGEQSHTKARDQLGEQEGNLGTQSSPAEETAQRPSKDHKVATEKGIKEHSKTQEPSLQAENE
Tml_TCHHL1    DNGAQLEPNKAMTTSEETSSPTKGEGQDKEIPREAEKPAWEQKGTKTRAQLGQQGGNLGTQSSPAEETVQRPYEDHKVATEKSVQEHSKIQGHPCKQKTS
 Dd_TCHHL1    DNGAQLETNKAMTTSEETSSPTKG----------------------------------------------------------------------------

              301                                                                                                400
 Hs_TCHHL1    PSSQHADLPEQAAARSPSQTQKSTDSKDVCRMFDTQEPGKDA------------------------------DQTPAKTKNLGEPEDYGRTSETQE---K
 Em_TCHHL1    SSSEQADLPEKGAEGKQSPTQKLTDPEDEGRISETQEPGKDANRESYESEKLTESVIDSRVPEVQEPGKGDADRTPPETKITAEPGDDGRISETQELPAQ
Tml_TCHHL1    PVQSMLTCQNKLLKVNHLRRRN------------------------------------------------------------------------------
 Dd_TCHHL1    ----------------------------------------------------------------------------------------------------

              401                                                                                                500
 Hs_TCHHL1    ECETKDLPVQYGSRNGSETSDMRDERKERRGPEAHGTAGQKERDRKTRPLVLETQTQDGKYQELQGLSKSKDAEKGSETQYLSSEGGDQTHPELEGTAVS
 Em_TCHHL1    EQETKDMPVQGDGKNISETHYVTTERKLGRGPETHGTTGQKGSKRKTQTPALEVQTQEGMYQELQGPSKERDTAKGTETQDLGSEGTDQSHTEIEGAAAL
Tml_TCHHL1    ----------------------------------------------------------------------------------------------------
 Dd_TCHHL1    ----------------------------------------------------------------------------------------------------

              501                                                                                                600
 Hs_TCHHL1    GEEAEHTKEGTAEAFVNSKNAPAAERTLGARERTQDLAPLEKQSVGENTRVTKTHDQPVEEEDGYQGEDPESPFTQSDEGSSETPNSLASEEGNSSSETG
 Em_TCHHL1    GD-VRYIEKGREEALVGSKNAPVAEGTAGARERTQESAPLESQSVGKKRRITKTQDKSTKEDDSYQGEDPVPPTTQNDEGFFKTPNNLAPEEGDSSLETG
Tml_TCHHL1    ----------------------------------------------------------------------------------------------------
 Dd_TCHHL1    ----------------------------------------------------------------------------------------------------

              601                                                                                                700
 Hs_TCHHL1    ELPVQGDSQSQGDQHGESVQGGHNNNPDTQRQGTPGEKNRALEAVVPAVRGEDVQLTEDQEQPARGEHKNQGPGTKGPGAAVEPNGHPEAQESTAGDENR
 Em_TCHHL1    DLPAQRDSQSQVDPPGESVQGGCNNDPDAQKQAESGEKYRAQEAVVLAVRGEAEQLTDEQEQFLRGEHKSWGSGTKGPDPDVEVNGLPEAQQSTAGSDNG
Tml_TCHHL1
 Dd_TCHHL1

              701                                                                                                800
 Hs_TCHHL1    KSLEIEITGALDEDFTDQLSLMQLPGKGDSRNELKVQGPSSKEEKGRATEAQNTLLESLDEDNSASLKIQLETKEPVTSEEEDESPQELAGEGGDQKSPA
 Em_TCHHL1    KSVKAEIPGALDADCNDQPSVMQQPAKGDSRKKLKVQGPGTKGEKGGESKTQETPLKSLNEDNSASSETHIE--EPATLKEEDENPQELA-EGDDQQHPT
Tml_TCHHL1    ----------------------------------------------------------------------------------------------------
 Dd_TCHHL1    ----------------------------------------------------------------------------------------------------

              801                                                                                                900
 Hs_TCHHL1    KKEHNSSVPWSSLEKQMQRDQEPCSVERGAVYSSPLYQYLQEKILQQTNVTQEEHQKQVQIAQASGPELCS----VSLTSEISDCSVFFNYSQASQPYTR
 Em_TCHHL1    KKGGYSSAPLPGLEERMQRDQQPYTVEKDLVHPSPSYTYLQEKIPQQIDITHEERQNQAQPAQASGPELSTDQSRASLTSEISNCLTIFYHSQALRRYTR
Tml_TCHHL1    ----------------------------------------------------------------------------------------------------
 Dd_TCHHL1    ----------------------------------------------------------------------------------------------------

              901                                       943
 Hs_TCHHL1    GLPLDESPAGAQETPAPQALEDKQGHPQRERLVLQREASTTKQ
 Em_TCHHL1    ELSPDEAPADPQQTSAL--------------------------
Tml_TCHHL1    -------------------------------------------
 Dd_TCHHL1    -------------------------------------------
```

Supplementary Figure S7. Alignment of amino acid sequences of SFTPs. Amino acid sequence alignment of **(A)** *CRNN*, **(B)** *FLG*, **(C)** *FLG2*, **(D)** *HRNR*, **(E)** *RPTN*, **(F)** *TCHH* and **(G)** *TCHHL1* of human, elephant, manatee and dugong. Amino acid residues and highly similar residues (D/N, D/E/Q, L/M, I/V, F/Y) conserved in all species are colored in red. Amino acid residues conserved in >50% of all investigated species are colored in blue. Residues of the evolutionarily ancestral C-termial motif with the core sequence: SPLY(E/D)Y, which is hypothesized to contribute to the interaction of SFTPs with keratin filaments (Takase and Hirai 2012; Mlitz et al. 2014; Mlitz et al. 2017) are shaded in green. Note that dugong FLG2 is truncated by a premature stop codon, leading to termination of the protein at the position indicated by an X on yellow background. The following amino acid sequence is obtained by translation of the downstream nucleotide sequence to show the similarity to other FLG2 proteins. Species: Human (*Homo sapiens*), elephant (*Elephas maximus indicus*), dugong (*Dugong dugon*), manatee (*Trichechus manatus latirostris*).

```
                  1                                                                                                  100
Human    ASPRV1 MAGSGARSEEGRRQHAFVPEPFDGANVVPNLWLHSFEVINDLNHWDHITKLRFLKESLRGEALGVYNRLSPQDQGDYGTVKEALLKAFGVPGAAPSHLPK
Elephant ASPRV1 MAGSGHRSQEGHRGQAFVPEPFDGANLAPHLWLHRLEVINYFNDWDHVTKLRYLKESLRGDALEVYRGLSPEDQGDYGVVKETLMKNFGGPSAAHSHLPK
Manatee  ASPRV1 MAGNGARSQEGHREHAFVPGPFDGANLAPHLWLHRFEVINDLNNWDHVTKLRFLKESLRGDALEVYRGLSPEDQGDYKVVKEILMKTFGVPSAAPSHLPK
Dugong   ASPRV1 MAGSGARSQEGHQEHAFVPGPFDGANLAPHLWLYRFEVINDLNNWDHVTQLRFLKDSLRGDALEVYRGLSPEDQGDYKVVKETLMKTFGDPSDAPSHLPK

                  101                                                                                                200
Human    ASPRV1 EIVFANSMGKGYYLKGKIGKVPVRFLVDSGAQVSVVHPNLWEEVTDGDLDTLQPFENVVKVANGAEMKILGVWDTAVSLGKLKLKAQFLVANASAEEAII
Elephant ASPRV1 EIVFANSMGKGYYLKGKIDKVPVRFLVDSGAQVSVVHPSLWEEVTDGDLDTLRPFENVVKVANGAEMKILGVWDTAVSLGKLKLKAEFLVANASAEEAII
Manatee  ASPRV1 EIIFANSMGKGYYLKGKIGKVPVRFLVDSGAQVSVVHPSLWEEVTDGDLDTLRPFKNVVKVANGAEMKILGVWNTVMSLGKLNLKAEFLVADTSAEEAII
Dugong   ASPRV1 EIIFANSMGKGYYLKGKIGNVPVRFLVDSGAQVSVVHPSLWEEVTDGDLDTLRPFKNVIKVANGAEMKILGVWNTVMSLDKLKLKAEFLVADTSAEEAII

                  201                                                       259
Human    ASPRV1 GTDVLQDHNAILDFEHRTCTLKGKKFRLLPVGGSLEDEFDLELIEEDPSSEEGRQELSH
Elephant ASPRV1 GTDVLQDHNAVLDFQHRTCTLKGKKFRLLPVGGSLEDEFDLELIEEEPSSQEGQEQLSY
Manatee  ASPRV1 GTDVLQDHNAILDFKHRICTLKGNNFRLLPVGGSLEDEFDLELIEEEPSSQEGQ-----
Dugong   ASPRV1 GTDVLQDHNAILDFKHRTCTLKGKKFRLLPVGGSLEDEFDLELIEEELSSQEGQ-----
```

Supplementary Figure S8. Amino acid sequence alignment of ASPRV1 in sirenians, elephant and humans. Amino acid sequence alignment of ASPRV1 proteins. Amino acid residues and highly similar residues (D/N, D/E/Q, L/M, I/V, F/Y) conserved in all species are colored in red. Amino acid residues conserved in >50% of all investigated species are colored in blue. Species: Human (*Homo sapiens*), elephant (*Elephas maximus indicus*), manatee (*Trichechus manatus latirostris*), dugong (*Dugong dugon*). GenBank accession numbers: Human ASPRV1, NP_690005.3; elephant ASPRV1, XP_049712741.1; manatee ASPRV1, XP_004391422.2. ASPRV1, the open reading frames of the ASPRV1 gene of the dugong was derived by translation of the nucleotide sequence of the gene (GenBank accession number: JASCZL010000012.1, nucleotides 106203165 – 106203929).

```
         1                                                                                                  100
mDugDug1 ATGGACAATGCTCAGCCGTTGGAGGAGGTGGGCTGGGTGCAGTTTGGGAGGGTAGGGCCATTTAGGAGTGGAGGTGAGGAGCTTAATATTCAGAGACAGT
CAJQER01 ATGGACAATGCTCAGCCGTTGGAGGAGGTGGGCTGGGTGCAGTTTGGGAGGGTAGGGCCATTTAGGAGTGGAGGTGAGGAGCTTAATATTCAGAGACAGT
  BMBL01 ATGGACAATGCTCAGCCGTTGGAGGAGGTGGGCTGGGTGCAGTTTGGGAGGGTAGGGCCATTTAGGAGTGGAGGTGAGGAGCTTAATATTCAGAGACAGT

         101                                                                                                200
mDugDug1 AGAGGGAATGAAAAATCAGGGTCTGAACTCTGAACTGGAAGGTACTTTGTGACCATGAAGACGGGGAAACAGACTCAGAGATAGAACCCCAGTAAGGGTG
CAJQER01 AGAGGGAATGAAAAATCAGGGTCTGAACTCTGAACTGGAAGGTACTTTGTGACCATGAAGACGGGGAAACAGACTCAGAGATAGAACCCCAGTAAGGGTG
  BMBL01 AGAGGGAATGAAAAATCAGGGTCTGAACTCTGAACTGGAAGGTACTTTGTGACCATGAAGACGGGGAAACAGACTCAGAGATAGAACCCCAGTAAGGGTG

         201                                                                                                300
mDugDug1 TGGGTGTGGGGGACAGATATATCTGCTTCCAATGATTGAGTTCTTATCACTATGATATTCAATACCCTGCTTCTCTTCTAAAAAGAAATTTTGATCCCAA
CAJQER01 TGGGTGTGGGGGACAGATATATCTGCTTCCAATGATTGAGTTCTTATCACTATGATATTCAATACCCTGCTTCTCTTCTAAAAAGAAATTTTGATCCCAA
  BMBL01 TGGGTGTGGGGGACAGATATATCTGCTTCCAATGATTGAGTTCTTATCACTATGATATTCAATACCCTGCTTCTCTTCTAAAAAGAAATTTTGATCCCAA

         301                                                                                                400
mDugDug1 TATCCATCCTCATATGGGATGAGATAGGATCTTAGTATTCGCTTCCGAAATTCTGAGACCTCTGCAAGTGAGGAAGCAATGGAGGAATGCTCAAAACACT
CAJQER01 TATCCATCCTCATATGGGATGAGATAGGATCTTAGTATTCGCTTCCGAAATTCTGAGACCTCTGCAAGTGAGGAAGCAATGGAGGAATGCTCAAAACACT
  BMBL01 TATCCATCCTCATATGGGATGAGATAGGATCTTAGTATTCGCTTCCGAAATTCTGAGACCTCTGCAAGTGAGGAAGCAATGGAGGAATGCTCAAAACACT

         401                                                                                                500
mDugDug1 ATTAGAAAAGATGGAGAGAGAGGCCAAGCCACCAAATGTGACAACTGGAAGGGACCTTCAAAATTCCCCATACATTCGGTGTCTGTATTTGGAAACTGCG
CAJQER01 ATTAGAAAAGATGGAGAGAGAGGCCAAGCCACCAAATGTGACAACTGGAAGGGACCTTCAAAATTCCCCATACATTCGGTGTCTGTATTTGGAAACTGCG
  BMBL01 ATTAGAAAAGATGGAGAGAGAGGCCAAGCCACCAAATGTGACAACTGGAAGGGACCTTCAAAATTCCCCATACATTCGGTGTCTGTATTTGGAAACTGCG

         501                                                                                                600
mDugDug1 GCTCAGATAGGAAAAATGACTTGCTCAAGTTCACACAGGGGCTGCAACAGAGCCATACCCCAGATCTGGTTGCCAGATTTGGCAAATAAAAATACAGAAT
CAJQER01 GCTCAGATAGGAAAAATGACTTGCTCAAGTTCACACAGGGGCTGCAACAGAGCCATACCCCAGATCTGGTTGCCAGATTTGGCAAATAAAAATACAGAAT
  BMBL01 GCTCAGATAGGAAAAATGACTTGCTCAAGTTCACACAGGGGCTGCAACAGAGCCATACCCCAGATCTGGTTGCCAGATTTGGCAAATAAAAATACAGAAT

         601                                                                                                700
mDugDug1 GTCCAGTTAAATTGAATTTTAGACAAACATCAAACAATTTTATAGTATAGGTATGTCCCATACAATATTGGGACATACTTATACTCAATTACCAGTTGTT
CAJQER01 GTCCAGTTAAATTGAATTTTAGACAAACATCAAACAATTTTATAGTATAGGTATGTCCCATACAATATTGGGACATACTTATACTCAATTACCAGTTGTT
  BMBL01 GTCCAGTTAAATTGAATTTTAGACAAACATCAAACAATTTTATAGTATAGGTATGTCCCATACAATATTGGGACATACTTATACTCAATTACCAGTTGTT

         701                                                                                                800
mDugDug1 TTTACCAGTTGCCATTGAGTTTATACCAACTCATGGCGACCCCATGTGCCATGTGTCAGAGTAGAACTGTGCTCCATAGTTTTCAATGGTTGGTTTTCTT
CAJQER01 TTTACCAGTTGCCATTGAGTTTATACCAACTCATGGCGACCCCATGTGCCATGTGTCAGAGTAGAACTGTGCTCCATAGTTTTCAATGGTTGGTTTTTTT
  BMBL01 TTTACCAGTTGCCATTGAGTTTATACCAACTCATGGCGACCCCATGTGCCATGTGTCAGAGTAGAACTGTGCTCCATAGTTTTCAATGGTTGGTTTTTTT

         801                                                                                                900
mDugDug1 TGGAAGCAGATCACCAGATCTTTCTTCCAAGGTACCTCTAGGTGGACTCGAACCTCCAACCTTTTGGTGAGCAGCTGAGCAGTTAATCGTTTGCACCACC
CAJQER01 TGGAAGCAGATCACCAGATCTTTCTTCCAAGGTACCTCTAGGTGGACTCGAACCTCCAACCTTTTGGTGAGCAGCTGAGCAGTTAATCGTTTGCACCACC
  BMBL01 TGGAAGCAGATCACCAGATCTTTCTTCCAAGGTACCTCTAGGTGGACTCGAACCTCCAACCTTTTGGTGAGCAGCTGAGCAGTTAATCGTTTGCACCACC

         901                                                                                               1000
mDugDug1 CAGTGATATTTGGGATATACCTGTACTCAGTCATTGTTTTTGTTAGTTGCCTTTGAGTCAGCTCAGATTCATGGTGACCTTTTGTATAACAACAAAACAT
CAJQER01 CAGTGATATTTGGGATATACCTGTACTCAGTCATTGTTTTTGTTAGTTGCCTTTGAGTCAGCTCAGATTCATGGTGACCTTTTGTATAACAACAAAACAT
  BMBL01 CAGTGATATTTGGGATATACCTGTACTCAGTCATTGTTTTTGTTAGTTGCCTTTGAGTCAGCTCAGATTCATGGTGACCTTTTGTATAACAACAAAACAT

         1001                                                                                              1100
mDugDug1 TGCCTGGTCCTGCACCATCTTTATGATCGTTTGTATGCTTGAGTCAATTGTTTTGGCTATTGTGTCAATACATCTCATTGAAGGTTTCTTTCATTTCCAC
CAJQER01 TGCCTGGTCCTGCACCATCTTTATGATCGTTTGTATGCTTGAGTCAATTGTTTTGGCTATTGTGTCAATACATCTCATTGAAGGTTTCTTTCATTTCCAC
  BMBL01 TGCCTGGTCCTGCACCATCTTTATGATCGTTTGTATGCTTGAGTCAATTGTTTTGGCTATTGTGTCAATACATCTCATTGAAGGTTTCTTTCATTTCCAC

         1101                                                                                              1200
mDugDug1 TGACCCTCTACTTTACCAACCATGATGTCCTTTTCTAGCAACTGGGTACCCAATCATAGCTGTACTTAAATTCAAGTTTAACTGGGTATTCTGTATTGTA
CAJQER01 TGACCCTCTACTTTACCAACCATGATGTCCTTTTCTAGCAACTGGGTACCCAATCATAGCTGTACTTAAATTCAAGTTTAACTGGGTATTCTGTATTGTA
  BMBL01 TGACCCTCTACTTTACCAACCATGATGTCCTTTTCTAGCAACTGGGTACCCAATCATAGCTGTACTTAAATTCAAGTTTAACTGGGTATTCTGTATTGTA

         1201                                                                                              1300
                                                                                                      E  V
mDugDug1 TCTTGCAATCCTATTCCCAGGCCTCTCTTGATCACATCCCCTACTTCTGCTTGATCTTTCTCCCTTTGCTGACTTCTGTCATCTCTTGGCTCAGGAGGTA
CAJQER01 TCTTGCAATCCTATTCCCAGGCCTCTCTTGATCACATCCCCTACTTCTGCTTGATCTTTCTCCCTTTGCTGACTTCTGTCATCTCTTGGCTCAGGAGGTA
  BMBL01 TCTTGCAATCCTATTCCCAGGCCTCTCTTGATCACATCCCCTACTTCTGCTTGATCTTTCTCCCTTTGCTGACTTCTGTCATCTCTTGGCTCAGGAGGTG
                                                                                                      E  V

         1301                                                                                              1400
          Y  D  M  S  G  A  H  L  A  L  I  L  C  V  T  K  A  R  E  V  S  E  A  D  L  A  A  L  E  H  M  F  Q
mDugDug1 TATGATATGTCGGGTGCCCACCTGGCCCTGATACTCTGTGTCACCAAAGCCCGGGAAGTTTCTGAAGCAGACCTGGCTGCCCTGGAACATATGTTCCAGA
CAJQER01 TATGATATGTCGGGTGCCCACCTGGCCCTGATACTCTGTGTCACCAAAGCCCGGGAAGTTTCTGAAGCAGACCTGGCTGCCCTGGAACATATGTTCCAGA
  BMBL01 TATGATATGTCGGGTGCCCACCTGGCCCTGATACTCTGTGTCACCAAAGCCCAGGAAGTTTCTGAAGCAGACCTGGCTGCCCTGGAACATATGTTCCAGA
          Y  D  M  S  G  A  H  L  A  L  I  L  C  V  T  K  A  Q  E  V  S  E  A  D  L  A  A  L  E  H  M  F  Q

         1401                                                                                              1500
         K  L  G  F  E  S  T  M  K  R  D  P  T  A  Q
mDugDug1 AGCTGGGATTTGAGAGCACCATGAAGAGAGATCCCACTGCCCAGGTATTGTGCTGCCAGCTCCAGACAAGGGAGGCTAGGGGCTGGACCAAAGGGTGGGC
CAJQER01 AGCTGGGATTTGAGAGCACCATGAAGAGAGATCCCACTGCCCAGGTATTGTGCTGCCAGCTCCAGACAAGGGAGGCTAGGGGCTGGACCAAAGGGTGGGC
  BMBL01 AGCTGGGATTTGAGAGCACCATGAAGAGAGATCCCACTGCCCAGGTATTGTGCTGCCAGCTCCAGACAAGGGAGGCTAGGGGCTGGACCAAAGGGTGGGC
         K  L  G  F  E  S  T  M  K  R  D  P  T  A  Q

         1501                                                                                              1600
mDugDug1 TTTGGGGTCCCGCTTGAGTCTGCCATTCCTCTCCCTCCAGCAATTCCCGGAAGAGTTGGATTAATTCCGGGAGGCCATGGAAGCCCGGACAGACCCCATC
CAJQER01 TTTGGGGTCCCGCTTGAGTCTGCCATTCCTCTCCCTTCAGCAATTCCCGGAAGAGTTGGATTAATTCCGGGAGGCCATGGAAGCCCGGACAGACCCCATC
  BMBL01 TTTGGGGTCCCGCTTGAGTCTGCCATTCCTCTCCCTTCAGCAATTCCCGGAAGAGTTGGATTAATTCCGGGAGGCCATGGAAGCCCGGACAGACCCCATC

         1601                                                                                              1700
mDugDug1 AGCTGTGCCTTTGTGGTGCTCATGGCGTATGGGTCAGAAAGCCTTCTCAAGGGTGAGGATGAGCAGATGGTCGAGCTGGATGACCTCTCTGAGGTCTTGA
CAJQER01 AGCTGTGCCTTTGTGGTGCTCATGGCGTATGGGTCAGAAAGCCTTCTCAAGGGTGAGGATGAGCAGATGGTCGAGCTGGATGACCTCTCTGAGGTCTTGA
  BMBL01 AGCTGTGCCTTTGTGGTGCTCATGGCGTATGGGTCAGAAAGCCTTCTCAAGGGTGAGGATGAGCAGATGGTCGAGCTGGATGACCTCTCTGAGGTCTTGA

         1701                                                                                              1800
mDugDug1 ACAACAAGAAATGCTGGGCCCTGAGAGCCAAACCCAAAGTGTACATCGTGCAGGCCTGTCGAGGAGGTGAGGACAGAGTCAAGAACATAGAACCCGGAGC
CAJQER01 ACAACAAGAAATGCTGGGCCCTGAGAGCCAAACCCAAAGTGTACATCGTGCAGGCCTGTCGAGGAGGTGAGGACAGAGTCAAGAACATAGAACCCGGAGC
  BMBL01 ACAACAAGAAATGCTGGGCCCTGAGAGCCAAACCCAAAGTGTACATCGTGCAGGCCTGTCGAGGAGGTGAGGACAGAGTCAAGAACATAGAACCCGGAGC

         1801                                                                                              1900
mDugDug1 ATGGAGCCCAGAGTCACCCATAACCAACACAAATCCCAGATCCAAGACCCAAGCCCCAGCTAAGCCTTGTGCATTCTTCCTTGCTCTACCAGACTGCCCT
CAJQER01 ATGGAGCCCAGAGTCACCCATAACCAACACAAATCCCAGATCCAAGACCCAAGCCCCAGCTAAGCCTTGTGCATTCTTCCTTGCTCTACCAGACTGCCCT
  BMBL01 ATGGAGCCCAGAGTCACCCATAACCAACACAAATCCCAGATCCAAGACCCAAGCCCCAGCTAAGCCTTGTGCATTCTTCCTTGCTCTACCAGACTGCCCT

         1901                                                                                              2000
mDugDug1 ATGGCCTTCTTTTCTCCCCATCTTTCTCTTCTCTGACTTTGCCTTCTCTCCTTCGTGTTTCAGAACAAAGGGACCCTGGAGAAACAATAAGTGGAGATGA
CAJQER01 ATGGCCTTCTTTTCTCCCCATCTTTCTCTTCTCTGACTTTGCCTTCTCTCCTTCGTGTTTCAGAACAAAGGGACCCTGGAGAAACAATAAGTGGAGATGA
  BMBL01 ATGGCCTTCTTTTCTCCCCATCTTTCTCTTCTCTGACTTTGCCTTCTCTCCTTCGTGTTTCAGAACAAAGGGACCCTGGAGAAACAATAAGTGGAGATGA
```

```
          2001                                                                                              2100
mDugDug1  GATCAGGATGATCACAAAAGAGAGCCCCAAAACCATCCCAACCTACACAGATGCCCTACACATCTACTCCACTGTAGAGGGTATGAGCTACCAGCCTACC
CAJQER01  GATCAGGATGATCACAAAAGAGAGCCCCAAAACCATCCCAACCTACACAGATGCCCTACACATCTACTCCACTGTAGAGGGTATGAGCTACCAGCCTACC
  BMBL01  GATCAGGATGATCACAAAAGAGAGCCCCAAAACCATCCCAACCTACACAGATGCCCTACACATCTACTCCACTGTAGAGGGTATGAGCTACCAGCCTACC

          2101                                                                                              2200
mDugDug1  CAGGCACAAACTGCAGCCTGGCCTCTGCTCCTCCTTTTCCTCTCACCCAATGCACCTGGCCCTGAGCCTCTCCTCTAGGATCTCCCTCAGTAGTAACCCC
CAJQER01  CAGGCACAAACTGCAGCCTGGCCTCTGCTCCTCCTTTTCCTCTCACCCAATGCACCTGGCCCTGAGCCTCTCCTCTAGGATCTCCCTCAGTAGTAACCCC
  BMBL01  CAGGCACAAACTGCAGCCTGGCCTCTGCTCCTCCTTTTCCTCTCACCCAATGCACCTGGCCCTGAGCCTCTCCTCTAGGATCTCCCTCAGTAGTAACCCC

          2201                                                                                              2300
mDugDug1  TCTCAGAGGCCATCCAACCATTCATTCACACACTCACCCATCCATTCACTTATCCATTCAACCCCCTATTCATCCATCCATCCACTTATCTACCCATCCA
CAJQER01  TCTCAGAGGCCATCCAACCATTCATTCACACACTCACCCATCCATTCACTTATCCATTCAACCCCCTATTCATCCATCCATCCACTTATCTACCCATCCA
  BMBL01  TCTCAGAGGCCATCCAACCATTCATTCACACACTCACCCATCCATTCACTTATCCATTCAACCCCCTATTCATCCATCCATCCACTTATCTACCCATCCA

          2301                                                                                              2400
mDugDug1  CCCATCCACCCACCTATCCATCAGCCCATCCATTCACCCACCGATCCATCCATCCGTTCACTGATGCACCACTTCCCCCCGCCACACTGCCGCAGCTTCC
CAJQER01  CCCATCCACCCACCTATCCATCAGCCCATCCATTCACCCACCGATCCATCCATCCGTTCACTGATGCACCACTTCCCCCCGCCACACTGCCGCAGCTTCC
  BMBL01  CCCATCCACCCACCTATCCATCAGCCCATCCATTCACCCACCGATCCATCCATCCGTTCACTGATGCACCACTTCCCCCCGCCACACTGCCGCAGCTTCC

          2401                                                                                              2500
mDugDug1  CTGAGCCCACCCCCTCATGATCTCTCTGGAATCCCCAGGGTACATTTCCTACAGACATGACAAGAATGGCTCCTATTTCATCCAGACCCTGGTAGATGTA
CAJQER01  CTGAGCCCACCCCCTCATGATCTCTCTGGAATCCCCAGGGTACATTTCCTACAGACATGACAAGAATGGCTCCTATTTCATCCAGACCCTGGTAGATGTA
  BMBL01  CTGAGCCCACCCCCTCATGATCTCTCTGGAATCCCCAGGGTACATTTCCTACAGACATGACAAGAATGGCTCCTATTTCATCCAGACCCTGGTAGATGTA

          2501                                                                                              2600
mDugDug1  GTCACTGAGTTGAAAGGACCCATCTTGAAGCTTCTGACAGAGGTGAGTCGAGAGGAGGTAACTGGAAGCACAGCCCAACCTGAGCAGGGAGAAAGCAGTA
CAJQER01  GTCACTGAGTTGAAAGGACCCATCTTGAAGCTTCTGACAGAGGTGAGTCGAGAGGAGGTAACTGGAAGCACAGCCCAACCTGAGCAGGGAGAAAGCAGTA
  BMBL01  GTCACTGAGTTGAAAGGACCCATCTTGAAGCTTCTGACAGAGGTGAGTCGAGAGGAGGTAACTGGAAGCACAGCCCAACCTGAGCAGGGAGAAAGCAGTA

          2601                                                                                              2700
mDugDug1  GAAGCTGAGTCTTTTCAAACCTTTCTCCATGTTCCCCTCCCATGCCTCCAGTCCCAAATCCAACCTGGGTCACAACACAGACACTAGGATGCCATCAACC
CAJQER01  GAAGCTGAGTCTTTTCAAACCTTTCTCCATGTTCCCCTCCCATGCCTCCAGTCCCAAATCCAACCTGGGTCACAACACAGACACTAGGATGCCATCAACC
  BMBL01  GAAGCTGAGTCTTTTCAAACCTTTCTCCATGTTCCCCTCCCATGCCTCCAGTCCCAAATCCAACCTGGGTCACAACACAGACACTAGGATGCCATCAACC

          2701                                                                                              2800
mDugDug1  AGCAATGATTTTCTCTAATGCCAAGATGTCCAAAGTCCTCAGTCTGTGAGATAATTTAGAGGAAATACAGACCCACTTCTATAAACAAATATCTATATAA
CAJQER01  AGCAATGATTTTCTCTAATGCCAAGATGTCCAAAGTCCTCAGTCTGTGAGATAATTTAGAGGAAATACAGACCCACTTCTATAAACAAATATCTATATAA
  BMBL01  AGCAATGATTTTCTCTAATGCCAAGATGTCCAAAGTCCTCAGTCTGTGAGATAATTTAGAGGAAATACAGACCCACTTCTATAAACAAATATCTATATAA

          2801                                                                                              2900
mDugDug1  TCACCCTTTGAATTTAACAAATGTTTGGACAATTTTTTTCTAATGATTGTGTGTTTTATTGTGGATTAAAAAAAATATGTAACAAGAATGTCCAACCAAT
CAJQER01  TCACCCTTTGAATTTAACAAATGTTTGGACAATTTTTTTCTAATGATTGTGTGTTTTATTGTGGATTAAAAAAAATATGTAACAAGAATGTCCAACCAAT
  BMBL01  TCACCCTTTGAATTTAACAAATGTTTGGACAATTTTTTTCTAATGATTGTGTGTTTTATTGTGGATTAAAAAAAATATGTAACAAGAATGTCCAACAAAT

          2901                                                                                              3000
mDugDug1  GATTTTATAGATATGATAATATAGGATGAGGCAATAAAGGGTCTCTCATCTTAGGACAAAG-TACCCACCTGCCCTGTTCTCTAGCCTTCTCCCTATAAG
CAJQER01  GATTTTATAGATATGATAATATAGGATGAGGCAATAAAGGGTCTCTCATCTTAGGACAAAG-TACCCACCTGCCCTGTTCTCTAGCCTTCTCCCTATAAG
  BMBL01  GATTTTATAGATATGATAATATAGGATGAGGCAATAAAGGGTCTCTCATCTTAGGACAAAGGTACCCACCTGCCCTGTTCTCTAGCCTTCTCCCTATAAG

          3001                                                                                              3100
mDugDug1  CCTATTACATCAACTCTCCAAGGTGGCCTAGCCCCAAGCTGACCACCCCTGTCATTGCAGGTGACACGGTGGATAGCAGGAGAGATAGTTCAGGAAGGGA
CAJQER01  CCTATTACATCAACTCTCCAAGGTGGCCTAGCCCCAAGCTGACCACCCCTGTCATTGCAGGTGACACGGTGGATAGCAGGAGAGATAGTTCAGGAAGGGA
  BMBL01  CCTATTACATCAACTCTCCAAGGTGGCCTAGCCCCAAGCTGACCACCCCTGTCATTGCAGGTGACACGGTGGATAGCAGGAGAGATAGTTCAGGAAGGGA

          3101                                                         3161
mDugDug1  AAGAAGAAAAGTGAATCCAGAAATCCAAAGCACCCTTCGGAAACAGCTCTATCTGCAGTAG
CAJQER01  AAGAAGAAAAGTGAATCCAGAAATCCAAAGCACCCTTCGGAAACAGCTCTATCTGCAGTAG
  BMBL01  AAGAAGAAAAGTGAATCCAGAAATCCAAAGCACCCTTCGGAAACAGCTCTATCTGCAGTAG
```

Supplementary Figure S9. Nucleotide sequence of *CASP14* in three dugong genome sequence assemblies. Nucleotide sequence alignment of the coding sequence and the intervening introns of the *CASP14* gene in the genome of the dugong, as determined by three sequencing projects. Nucleotides that are unique for only one of the 3 sequence assemblies are highlighted by blue shading. In the mDugDug1 sequence, the start and stop codons are highlighted by green and red shading, respectively, and the rest of the coding sequence is highlighted by yellow shading. Splicing signals at the ends of introns (GT and AG) are underlined. Note that the non-coding exon 1 is not included. The amino acid sequence encoded by exon 3, which is the second coding exon, is shown with red fonts. Sequences: mDugDug1, GenBank accession number: JASCZL010000003.1, nucleotides 19499292-19502451; CAJQER01, GenBank accession number: CAJQER010001325.1, nucleotides 195450-198610; BMBL01, GenBank accession number: BMBL01082170.1, nucleotides 6999-10158.
